# New Fusarochromanone Derivatives from the Marine Fungus *Fusarium equiseti* UBOCC-A-117302

**DOI:** 10.3390/md22100444

**Published:** 2024-09-28

**Authors:** Giang Nam Pham, Béatrice Josselin, Arnaud Cousseau, Blandine Baratte, Marie Dayras, Christophe Le Meur, Stella Debaets, Amélie Weill, Thomas Robert, Gaëtan Burgaud, Ian Probert, Fatouma Mohamed Abdoul-Latif, Laurent Boyer, Stéphane Bach, Mohamed Mehiri

**Affiliations:** 1 Marine Natural Products Team, Institut de Chimie de Nice, Université Côte d’Azur, CNRS, UMR 7272, 06108 Nice, France; giangnampham94@gmail.com (G.N.P.); arnaud.cousseau@outlook.com (A.C.);; 2 Integrative Biology of Marine Models Laboratory (LBI2M), Station Biologique de Roscoff, Sorbonne Université, CNRS, UMR 8227, 29680 Roscoff, Francebaratte@sb-roscoff.fr (B.B.);; 3 Plateforme de Criblage KISSf (Kinase Inhibitor Specialized Screening Facility), Station Biologique de Roscoff, Sorbonne Université, CNRS, FR2424, 29680 Roscoff, France; 4 Laboratoire Universitaire de Biodiversité et Écologie Microbienne, Université de Brest, INRAE, 29280 Plouzané, France; 5 Roscoff Culture Collection, Station Biologique de Roscoff, Sorbonne Université, CNRS, FR2424, 29680 Roscoff, France; 6 Medicinal Research Institute, Center for Studies and Research of Djibouti, IRM-CERD, Route de l’Aéroport, Haramous, Djibouti City P.O. Box 486, Djibouti; fatouma_abdoulatif@yahoo.fr; 7 INSERM U1065, Centre Méditerranéen de Médecine Moléculaire (C3M), Bâtiment Universitaire ARCHIMED, 151 Route de Saint Antoine de Ginestière BP, 23194 Nice, France

**Keywords:** *Fusarium equiseti*, fusarochromanone, cytotoxicity, antimicrobial activity, protein kinase inhibitors

## Abstract

Two new fusarochromanone derivatives, deacetylfusarochromene (**1**) and deacetamidofusarochrom-2′,3-diene (**2**), along with the previously reported metabolites fusarochromanone TDP-2 (**3**), fusarochromene (**4**), 2,2-dimethyl-5-amino-6-(2′*E*-ene-4′-hydroxylbutyryl)-4-chromone (**5**), fusarochromanone (**6**), (−)-chrysogine (**7**), and equisetin (**8**), were isolated from the marine fungus *Fusarium equiseti* UBOCC-A-117302. The structures of the compounds were determined by extensive spectrometric (HRMS) and spectroscopic (1D and 2D NMR) analyses, as well as specific rotation. Among them, **2** and **5** showed inhibition of three protein kinases with IC_50_ values ranging from 1.42 to 25.48 μM. Cytotoxicity and antimicrobial activity of all isolated compounds were also evaluated. Six fusarochromanone derivatives (**1**–**6**) exhibited diverse activities against three cell lines, RPE-1, HCT-116, and U2OS (IC_50_ values ranging from 0.058 to 84.380 μM). Equisetin (**8**) showed bactericidal activities against *Bacillus cereus* and *Listeria monocytogenes* (MBC values of 7.8 and 31.25 µM, respectively), and bacteriostatic activity against *Enterococcus faecalis* (MIC value of 31.25 µM). Compounds **2** and **4** showed bacteriostatic activities against *Listeria monocytogenes* (MIC of 125 µM).

## 1. Introduction

Marine fungi represent a non-trivial source of biologically active metabolites due to their extensive chemodiversity, which offers structural and functional uniqueness [1]. Specialized metabolites from marine fungi have been reported from several genera, the most represented being *Penicillium*, *Aspergillus*, *Cladosporium*, *Fusarium*, *Trichoderma*, *Acremonium*, *Phoma*, and *Chaetomium* [2]. Mycotoxins are toxic specialized metabolites produced by numerous filamentous fungi, of which representatives of the genera *Fusarium*, *Aspergillus*, and *Penicillium* are the main producers. Several studies have reported a wide range of metabolites from both terrestrial and marine *F. equiseti* strains, including fusarochromanone derivatives [3,4], tetramic acid compounds like equisetin and 5′-*epi*equisetin [5,6], fusarisetins A-D [7,8], fusaketide A [8], pestalotiollides A and B [8], decalintetracid A [9], and various polyketides such as decalintetracid B [10], fusaequisin A [10], neofusapyrone [10], deoxyneofusapyrone [10], and indole alkaloids [11,12], notably fusarindoles A–E [12]. The structure of fusarochromanone is unique due to the presence of two geminal methyl groups at C-2 and the alternating β-keto-amine groups. Fusarochromanone exhibits potent antiangiogenic and antitumor activity [13]. Recent studies demonstrated that fusarochromanone exhibits significant in vitro growth inhibitory effects against glioblastomas and melanomas through induction of apoptosis and different pathways [13,14,15,16]. Moreover, fusarochromanone induces cell death by activating the JNK pathway, which is triggered by the production of reactive oxygen species (ROS) [14]. Fusarochromanone-induced ROS inhibits protein phosphatases 2A (PP2A) and 5 (PP5), leading to JNK pathway activation [14]. The amine group at C-3′ is important for its biological activities, since acetylated metabolites exhibited fewer biological effects [17]. Therefore, fusarochromanone is considered as an excellent lead candidate [18], and discovering additional derivatives would provide valuable insights into its structure–activity relationships.

In this context, we investigated the metabolites produced by the marine fungus *Fusarium equiseti* UBOCC-A-117302. A combination of UHPLC-MS/MS analyses with a feature-based molecular networking–Global Natural Products Social (FBMN-GNPS) approach allowed us to detect the presence of putative new fusarochromanone derivatives in the crude organic extract. Herein, we report the isolation of two new fusarochromanone derivatives, deacetylfusarochromene (**1**) and deacetamidofusarochrom-2′,3-diene (**2**), along with previously reported metabolites, fusarochromanone TDP-2 (**3**), fusarochromene (**4**), 2,2-dimethyl-5-amino-6-(2′*E*-ene-4′-hydroxylbutyryl)-4-chromone (**5**), fusarochromanone (**6**), (−)-chrysogine (**7**), and equisetin (**8**). All the isolated compounds were evaluated for their capacity to inhibit disease-relevant protein kinases, their cytotoxicity, and their antimicrobial properties.

## 2. Results and Discussion

### 2.1. FBMN Analysis and Metabolite Annotation

**Figure 1:**
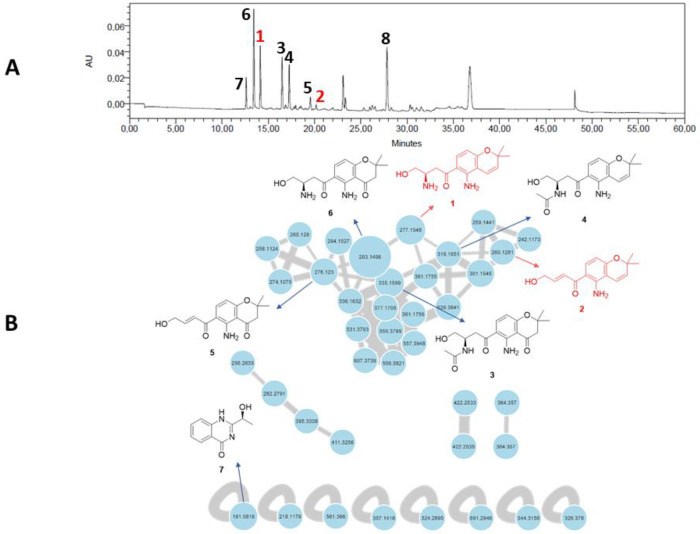
(**A**) HPLC–UV chromatogram at 280 nm of the crude organic extract of *Fusarium equiseti* UBOCC-A-117302 (column: NUCLEODUR Sphinx RP 250 × 4.6 mm; mobile phase: H_2_O-acetonitrile, containing 0.1% formic acid: 90:10 for 5 min, 90:10 to 0:100 over 35 min, then 90:10 for 10 min, with reconditioning until 60 min; flow rate: 1 mL/min; injection volume: 20 µL). (**B**) Feature-based molecular networking of the crude culture organic extract of *F. equiseti* UBOCC-A-117302 by ultra-high-performance liquid chromatography coupled to high-resolution tandem mass spectrometry (UHPLC-HRMS/MS) in positive ionization mode (common fragment number: 5; similarity score: 0.5). Node size represents semiquantitative differences in metabolite concentrations in MS1 scan. Edge thickness indicates cosine score similarity between nodes. New molecules are in red, known molecules are in black.

A feature-based molecular networking (FBMN) analysis was performed to highlight chromone derivatives produced by *F. equiseti* in Czapek–Dox medium and guide their isolation. The FBMN approach was used to distinguish possible isomers in the network based on their retention time [19]. The graphical representation of the molecular network allowed us to highlight 39 nodes, of which 31 are linked together (Figure 1). The main cluster, dedicated to chromone derivatives, is constituted by 31 nodes, among which 4 nodes have been manually annotated as putatively fusarochromanone TDP-1 (**6**), fusarochromanone TDP-2 (**3**), fusarochromene (**4**), and 2,2-dimethyl-5-amino-6-(2′*E*-ene-4′-hydroxylbutyryl)-4-chromone (**5**), which are already known to be produced by fungi affiliated to the *Fusarium* genus. This cluster includes several spectral nodes with yet unknown molecular ion features that we set out to isolate in order to evaluate their biological activities.

### 2.2. Structure Elucidation

**Figure 2:**
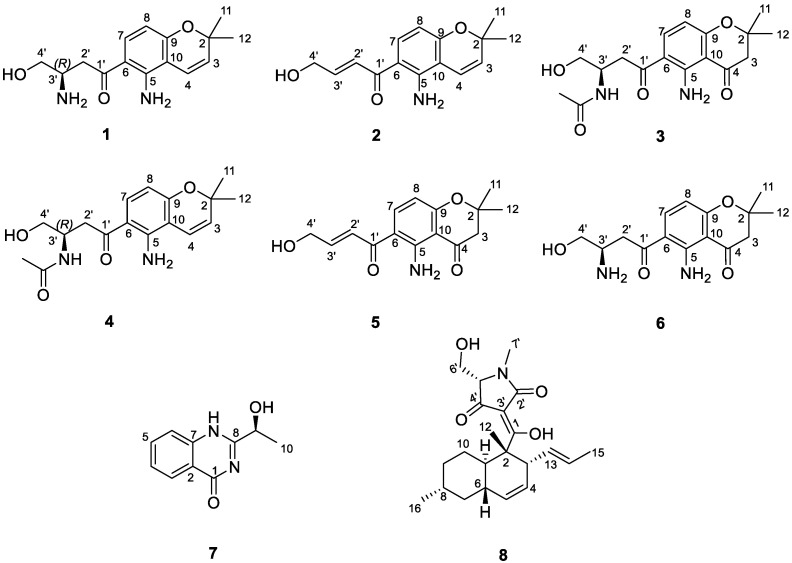
Structure of isolated compounds (**1**−**8**) from the fungus *Fusarium equiseti* UBOCC-A-117302.

**Figure 3:**
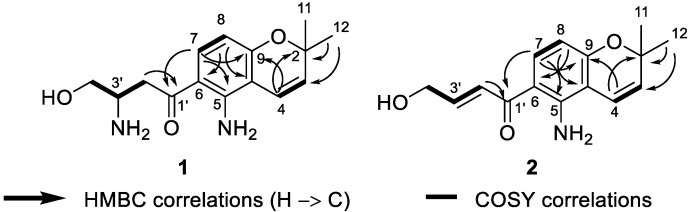
Key ^1^H-^1^H COSY and ^1^H-^13^C HMBC correlations of **1** and **2**.

Deacetylfusarochromene (**1**) was obtained as a yellow gum. Its molecular formula, C_15_H_20_N_2_O_3_, was deduced from the HRESI(+)MS analysis which showed a pseudo-molecular ion peak at *m*/*z* 277.1535 [M + H]^+^ (calcd for C_15_H_21_N_2_O_3_^+^ 277.1547, Δ = 4.33 ppm) (Figure S2). The resonance signals in the ^1^H NMR spectrum and their ^1^H-^13^C HSQC correlations exhibited two methyl groups at *δ*_H_ 1.40 (6H, s, H-11, H-12), two methylenes, of which one was neighbor to a ketone group at *δ*_H_ 3.36 (1H, m, H-2′a), 3.21 (1H, dd, *J* = 18.0 Hz, 8.3 Hz, H-2′b) and the other was oxygenated methylene at *δ*_H_ 3.82 (1H, m, H-4′a), 3.67 (1H, dd, *J* = 10.7 Hz, 5.5 Hz, H-4′b), five methines including one pair of ortho-coupled aromatic protons at *δ*_H_ 7.61 (1H, d, *J* = 9.0 Hz, H-7), 6.12 (1H, d, *J* = 9.0 Hz, H-8), one pair of cis-coupled olefinic protons at *δ*_H_ 6.58 (1H, d, *J* = 10.0 Hz, H-4), 5.65 (1H, d, *J* = 10.0 Hz, H-3), and one nitrogen-containing methine at *δ*_H_ 3.77 (1H, m, H-3′) (Table 1, Figures S2 and S5). The ^13^C spectrum revealed fifteen carbon signals, including one ketone at *δ*_C_ 198.0 (C-1′), six aromatic carbons at *δ*_C_ 150.2 (C-5), 112.8 (C-6), 133.9 (C-7), 106.9 (C-8), 159.9 (C-9), and 107.6 (C-10), two olefinic at *δ*_C_ 128.9 (C-3) and 117.1 (C-4), three hetero-bearing aliphatic carbons at *δ*_C_ 77.6 (C-2), 51.2 (C-3′), and 62.7 (C-4′), one further methylene at *δ*_C_ 38.5 (C-2′), and one methyl signal which stemmed from the two methyl carbons at *δ*_C_ 28.0 (C-11, C-12) (Figure S3). The 1D and 2D NMR data of **1** closely resembled those of fusarochromene (**4**) [20], except for the lack of signals of an acetyl group. The ^1^H-^13^C HMBC correlations of H-4 to C-2, C-9; H-11 and H-12 to C-2, C-3; H-7 to C-5, C-9; and H-8 to C-6, C-10 (Figure 2 and Figure S12) confirmed the presence of a 5-amino-chromene moiety. The continuous spin system H_2_-2′/H-3′/H_2_-4′ in the ^1^H-^1^H COSY spectrum (Figure 2 and Figure S5), and the ^1^H-^13^C HMBC correlations of H_2_-2′ to C-1′ suggested the arrangement of the side chain (Figure 3 and Figure S7). The ^1^H-^13^C HMBC correlation of H-7 to C-1′ revealed the connection between the chromene moiety and the side chain. In order to assign the absolute configuration of **1**, we first tried the Mosher’s method. Our attempts to purify the products of Mosher’s reaction were unsuccessful due to the limited amount available. The specific rotation of **1** was negative ([α]_D_^20^ −30.0 (*c* 0.3, MeOH)), which was the same sign as the reported value for fusarochromene (**4**) ([α]_D_^20^ −14.0 (*c* 0.1, MeOH)) [20]. Based on this observation, the absolute configuration of C-3′ is likely *R*. This assumption is further supported by the biosynthetic pathway of fusarochromanone (**6**) [20]. Therefore, the structure of **1** was fully elucidated, as shown in Figure 1.

Deacetamidofusarochrom-2′,3-diene (**2**) was isolated as a yellow gum. Its molecular formula, C_15_H_17_NO_3_, was determined from the HRESI(+)MS analysis which showed a pseudo-molecular ion peak at *m*/*z* 260.1271 [M + H]^+^ (calcd for C_15_H_18_NO_3_^+^ 260.1281, Δ = 3.84 ppm) (Figure S9). Compared to **1**, the molecular formula of **2** revealed the disappearance of the amine group on the side chain, and the resonances of the hetero-bearing methine had been replaced by trans-coupled olefinic protons at *δ*_H_ 7.23 (1H, dt, *J* = 15.2 Hz, 2.0 Hz, H-2′), 6.90 (1H, dt, *J* = 15.2 Hz, 4.1 Hz, H-3′) in the ^1^H NMR spectrum (Table 1, Figure S10). The downfield resonance of oxygenated methylene at *δ*_H_ 4.33 (2H, dd, *J* = 4.1 Hz, 2.2 Hz, H-4′) and the ^1^H-^1^H COSY cross-peaks H_2_-2′/H-3′/H_2_-4′ also confirmed the arrangement of a side-chain moiety (Figure S12). The chromene ring of **2** resembled that of **1**, which was confirmed by the ^1^H-^1^H COSY and ^1^H-^13^C HMBC correlations (Figure 3). Consequently, the structure of **2** was fully elucidated, as shown in Figure 1.

An extensive examination of the NMR, HRMS, and optical rotation data of **3**–**8** and comparison with previously published data [3,4,5,20,21,22] led to their identification as fusarochromanone TDP-2 (**3**), fusarochromene (**4**), 2,2-dimethyl-5-amino-6-(2′*E*-ene-4′-hydroxylbutyryl)-4-chromone (**5**), fusarochromanone TDP-1 (**6**), (−)-chrysogine (**7**), and equisetin (**8**), respectively.

### 2.3. Biological Assays

Compounds **1**–**8** were evaluated for (i) their capacity to inhibit disease-relevant protein kinases, (ii) their impact on the viability of cancerous and non-cancerous cell lines, and (iii) their antimicrobial properties.

**Figure 4:**
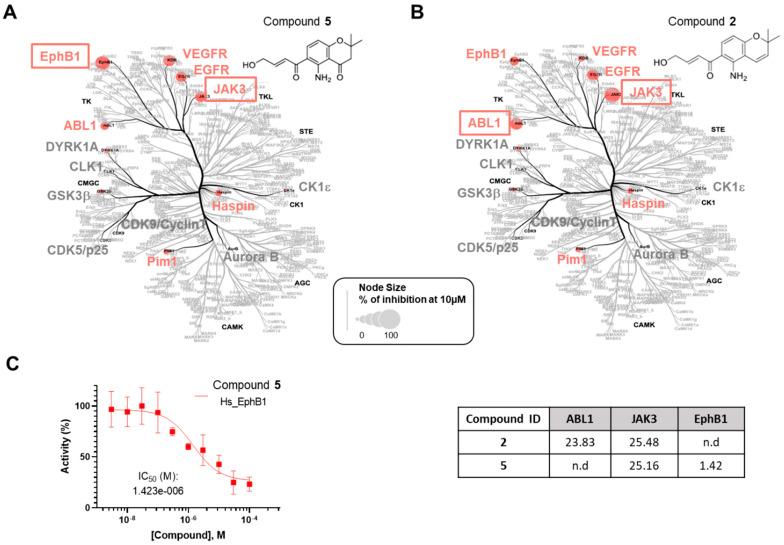
Selectivity analysis of compounds **2** and **5**. The compounds **5** (**A**) and **2** (**B**) were tested in a primary screening against a panel of 14 disease-related protein kinases. Only the results obtained for 10 µM are reported on an artistic representation of the human kinome phylogenetic tree. This visualization of the human kinome was generated using Coral [27], a user-friendly web application (web application available at http://phanstiel-lab.med.unc.edu/CORAL/, accessed on 20 July 2024). Quantitative data extracted from Table S1 were encoded in node size as mentioned in the legend of the figure. The codes reported in black on this figure indicate the subclasses of protein kinases: CMGC for CDKs, MAP kinases, and GSK and CDK-like kinases; AGC for protein kinase A, G, and C families (PKA, PKC, PKG); CAMK for Ca^2+^/calmodulin-dependent protein kinases; CK1, casein kinase 1; STE, STE kinases (homologs of yeast STErile kinases); TKL, tyrosine kinase-like; TK, tyrosine kinases. Each kinase tested in the assay panel is written in large font; kinases colored in gray are not affected by the tested compound and kinases colored in red (“hit kinases”) are inhibited by the tested compounds. (**C**) IC_50_ (µM) for compounds **2** and **5** against the selected protein kinases. Only the curve showing the inhibition of EphB1 tyrosine kinase by compound **5** is depicted in the figure. Kinase activities reported on the graph are expressed in % of maximal activity, i.e., measured in the absence of compound **5**.

All the compounds were first evaluated for their inhibitory activities against a panel of 14 disease-related protein kinases, namely CDK5/p25, CDK9/Cyclin T, HASPIN, DYRK1A, AURKB, GSK3β, EGFR, ABL1, JAK3, EphB1, VEGFR2, Pim1, CK1ε, and CLK1, at preliminary concentrations of 1 and 10 µM (Table S1). Among them, only deacetamidofusarochrom-2′,3-diene (**2**) and 2,2-dimethyl-5-amino-6-(2′*E*-ene-4′-hydroxylbutyryl)-4-chromone (**5**), both featuring a Δ^2′,3′^ double bond, exhibited activity against ABL1 and JAK3, and JAK3 and EphB1, respectively. The IC_50_ values (µM) were determined for compounds **2** and **5** against the selected protein kinases (Figure 4 and Figure S51). Deacetamidofusarochrom-2′,3-diene (**2**) showed moderate activities against both ABL1 and JAK3 (IC_50_ values of 23.83 and 25.48 µM, respectively). ABL1 has been identified as an oncogene associated with chromosome translocations in human leukemias [23]. JAK3 is associated with the immune response, is mainly expressed in hematopoietic tissue cells, and is a promising target for the treatment of autoimmune disease [24]. Both ABL1 and JAK3 kinases are primary targets of FDA-approved drugs, such as Imatinib for BCR/ABL-1 oncogenic protein and ruxolitinib for JAK3 [25]. Compound **5** was found to be a low micromolar inhibitor of EphB1 (IC_50_ of 1.42 µM) and showed a moderate activity against JAK3 (IC_50_ of 25.16 µM). EphB1 is a receptor tyrosine kinase that plays an important role in many biological processes, including angiogenesis, nervous system development, and the formation and maturation of neural synapses [26]. This is the first time that fusarochromanone derivatives have demonstrated their ability to inhibit disease-relevant protein kinases, which is of value for the development of new drugs.

Compounds **1**–**8** were also evaluated for their capacity to affect the cell viability of two human cancerous cell lines, U-2 OS (osteosarcoma) and HCT-116 (colorectal cancer), and a non-cancerous cell line, hTERT RPE-1 (retinal pigmented epithelial cells immortalized with hTERT) (Table S2). Chrysogine (**7**) and equisetin (**8**) showed no cytotoxicity at 25 µM. The EC_50_ (half-maximal effective concentration) values (µM) were determined for compounds **1**–**6** based on the percentage of cell viability (<80%) (Table 2). Deacetamidofusarochrom-2′,3′-dienes (**2**) and 2,2-dimethyl-5-amino-6-(2′*E*-ene-4′-hydroxylbutyryl)-4-chromone (**5**) showed moderate activities (EC_50_ from 5.22 µM to 13.73 µM). Fusarochromanone TDP-1 (**6**) exhibited the highest cytotoxicity, with EC_50_ values of 0.058 µM (RPE-1), 0.170 µM (HCT-116), and 0.232 µM (U2OS). In the literature, fusarochromanone TDP-1 (**6**) has been reported to exhibit strong cytotoxic activities against several cell lines [13,14,15,16,28,29]. Fusarochromanone TDP-2 (**3**), the 3′-acetylated derivative of fusarochromanone TDP-1 (**6**), was approximately 300-fold less active compared to (**6**) on average, with EC_50_ values of 23.140 µM (RPE-1), 62.950 µM (HCT-116), and 35.090 µM (U2OS). Similarly, deacetylfusarochromene (**1**) exhibited approximately 370-fold higher cytotoxicity compared to fusarochromene (**4**), with EC_50_ values of 0.176 µM (RPE-1), 0.087 µM (HCT-116), and 0.896 µM (U2OS). Our results clearly suggest that in terms of structure–activity relationships, acetylation of the 3′-amino group reduces drastically the cytotoxicity of both chromone and chromene derivatives, which is consistent with previously reported observations for fusarochromanone TDP-2 (**3**) and fusarochromanone TDP-1 (**6**) [17]. The side chain at C-6 was unique in nature [4,30], and appears to be essential for the biological activities. Chromone and chromene derivatives featuring a Δ^2′,3′^ double bond are, on average, 100-fold less cytotoxic compared to their counterparts with a 3′-amino group. Additionally, derivatives featuring a chromone scaffold exhibit 2- to 10-fold higher cytotoxicity compared to those with a chromene scaffold. We notice that there is no correlation between the cell viability and kinase assay results, indicating that the tested kinases were probably not involved in the observed cellular phenotype.

All the compounds were finally evaluated for their antimicrobial activities against a panel of microorganisms, such as Gram-negative bacteria (*Escherichia coli* ATCC 25922, *Salmonella enterica* CIP 8297, and *Pseudomonas aeruginosa* ATCC 27853), Gram-positive bacteria (*Staphylococcus aureus* ATCC25923, *Enterococcus faecalis* CIP A 186, *Bacillus cereus* ATCC 6464, and *Listeria monocytogenes* SOR 100), and the yeast *Candida albicans* ATCC 90028. Only compounds **2**, **4**, and **8** exhibited moderate bactericidal or bacteriostatic activity against a few strains (Table 3). 

Equisetin (**8**) showed the highest antibacterial activities against *B. cereus* (MBC of 7.8 µM) and *L. monocytogenes* (MBC of 31.25 µM), respectively, as well as moderate bacteriostatic activity against *E. faecalis* (MIC of 31.25 µM), which is consistent with previous studies that highlighted equisetin (**8**) as exclusively active against Gram-positive bacteria [31], including multi-drug-resistant Gram-positive bacteria. Surprisingly, although equisetin (**8**) has been identified as an efficient molecule against methicillin-resistant *S. aureus* (MRSA), even more effective than vancomycin [32], no activity against *S. aureus* ATCC25923 was detected for equisetin (**8**) in our study. While antibacterial activities of equisetin (**8**) against *Enterococcus* and *Bacillus* representatives have already been assessed [32], the detection of a bactericidal activity against *Listeria monocytogenes* appears original and calls for further studies to delve deeper into the mode of action of such a compound on *L. monocytogenes*, as well as compounds **2** and **4**, which showed specific bacteriostatic activities against the same bacterial target.

In terms of valorization potential, equisetin (**8**) definitely ticks many boxes since (i) it is active against a wide panel of Gram-positive bacteria, including MRSA and vancomycin-resistant *Enterococcus faecalis*, without detectable resistance [32], (ii) its combination with colistin potentiates colistin activity against colistin-resistant Gram-negative bacteria [31], and (iii) its mode of action relies on the inhibition of bacterial acetyl-CoA carboxylase [33] but also intracellular elimination by potentiating host autophagy and ROS generation [34]. This last result can guide further research studies on the effects of equisetin (**8**), deacetamidofusarochrom-2′,3-diene (**2**), and fusarochromene (**4**) on *L. monocytogenes*, given that this bacterium is particularly sensitive to ROS in terms of biofilm formation [35] or invasion [36].

## 3. Materials and Methods

### 3.1. General Experimental Procedure

HPLC-grade solvents and reagents were purchased from Sigma-Aldrich (Merck KGaA, Saint-Louis, MO, USA). Medium-Pressure Liquid Chromatography (MPLC) was performed on an Interchim PuriFlash^®^ chromatography system using a PF-30DIOL/120G cartridge. HPLC analyses and semi-preparative purifications were performed using a Waters Alliance e2695 HPLC system (Waters Corporation, Milford, MA, USA) coupled with a Waters 2998 photodiode array (PDA) detector and a Waters 2424 ELS detector. Analyses were performed with a bifunctional Macherey-Nagel NUCLEODUR Sphynx RP column (250 × 4.6 mm, 5 µm) consisting of a balanced ratio of propylphenyl and C18 ligands. Purifications were performed with a bifunctional Macherey-Nagel NUCLEODUR Sphynx RP (250 × 10 mm, 5 µm). NMR spectra were recorded with a 400 MHz Bruker Avance NMR spectrometer (Bruker Corporation, Billerica, MA, USA). High-resolution mass spectra (HRMS) were acquired with a Thermo Q-Exactive Focus (UPLC-HRMS) Orbitrap (Thermo Fisher Scientific, Waltham, MA, USA) using a Thermo Fisher Scientific Hypersil GOLD (150 × 2.1 mm, 1.9 μm) column and a mobile phase A H_2_O + 0.1% formic acid (UPLC/MS grade) and B ACN + 0.1% formic acid (UPLC/MS grade), pumped at a rate of 0.2 mL/min with the following gradient: 0–5 min, 10% B; 5–30 min, 10% to 98% B; 30–35 min, 98% B; and a column reconditioning phase to 10% B for 10 min. MS parameters were set as follows: spray voltage at 3.7 kV (positive mode) or 2.7 kV (negative mode), capillary temperature at 320 °C, a sheath gas rate at 60 units N_2_ (ca. 200 mL/min), and an auxiliary gas rate at 15 units N_2_ (ca. 50 mL/min). The *m*/*z* range for data-dependent acquisition was set between 100 and 1200 amu. Data were analyzed using Thermo Xcalibur software 2.2.44. Optical rotations were recorded on an Anton Paar MCP 150 polarimeter (Anton Paar, Graz, Austria).

### 3.2. Isolation and Identification of the Fungus

The isolate of *F. equiseti* examined in this study was initially derived from a seawater sample collected in the coastal region of Chile (south-east Pacific), and co-cultured with the cosmopolitan coccolithophore *Emiliania huxleyi*. Seawater samples were collected during a 2011 expedition near Tongoy Bay. It is important to note that Chile is not a party to the Nagoya Protocol, so no Access and Benefit Sharing authorization is required. This isolate was cryopreserved in the UBO Culture Collection (https://www.univ-brest.fr/ubocc/fr, accessed on 26 August 2024) under the accession number UBOCC-A-117302. Following isolation, *F. equiseti* CHC155 was further cultured on PDA supplemented with 3% sea salts to obtain biomass from which DNA was extracted using the FastDNA™ Spin Kit (MP Biomedicals, Illkirch-Graffenstaden, France) following manufacturer recommendations. The translation elongation factor 1-alpha gene (TEF1-α) was amplified and sequenced using the EF1-EF2 primers [37] and the following PCR conditions: initial denaturation at 94 °C for 5 min, followed by 35 cycles of 95 °C for 30 s, 57 °C for 1 min, 72 °C for 1 min, and a final extension at 72 °C for 7 min. The amplified products were then sequenced in both directions (Eurofins). The sequence of EF1-alpha was analyzed with BLAST, the GenBank database (https://blast.ncbi.nlm.nih.gov, accessed on 26 August 2024), and allowed to identify *F. equiseti* CHC155 with a percentage of identification of 100%. The sequence was deposited in the NCBI database under the accession number OQ290813.

### 3.3. Fermentation

The marine fungus *F. equiseti* UBOCC-A-117302 was fermented in Czapek–Dox media, following an adaptation of the method previously described [38]. 

### 3.4. Global Natural Products Social Molecular Networking

Data were processed by using the FBMN method [19]. The data files were transformed to mzXML format using the MSConvert package of the software ProteoWizard package 3.0. Subsequently, all mzXML values were processed using MZmine 2.53 [39]. Mass detection was performed at noise levels of 2 × 10^7^ and 2 × 10^4^ for MS^1^ and MS^2^, respectively. Chromatogram building was realized using the ADAP Chromatogram Builder Module with a minimum group size of 5, a group intensity threshold of 3x10^7^, a minimum highest intensity of 9 × 10^7^, and *m*/*z* tolerance of 0.01 (or 10 ppm). For chromatogram deconvolution, the baseline cut-off algorithm was selected with the following settings: minimum peak height of 3 × 10^8^, peak duration range of 0.1–2 min, baseline level of 1 × 10^8^, and an auto *m*/*z* center calculation. The *m*/*z* and RT tolerance range were set at 0.02 Da and 0.1 min, respectively, for MS/MS scan paring. Isotopologues were grouped using the isotopic peak grouper algorithm with an RT tolerance of 0.2 min and an *m*/*z* tolerance of 0.01 (or 10 ppm). After deisotoping, the feature list rows were filtered, keeping only peaks with MS^2^ scans. Peak alignment was performed using the join aligner with an *m*/*z* tolerance of 0.01 (or 10 ppm), a weight for *m*/*z* at 75%, a RT tolerance of 0.2 min, and a weight for RT at 25%. The generation of the MGF file and metadata was performed through the export/submit to GNPS option [40]. Molecular network calculation and visualization were executed using Cytoscape 3.8.1 software [41]. The parent mass and the MS^2^ fragment ion tolerances were both 0.02 Da. Edges were filtered to have a cosine score above 0.5 and more than 5 matched peaks.

### 3.5. Extraction and Purification

The cultured Czapek–Dox medium was extracted with dichloromethane/ethyl acetate (1:1, *v*/*v*). The total extract (2.35 g) was fractionated by flash chromatography using a PF-30DIOL/120G cartridge, eluting with cyclohexane/ethyl acetate (100:0 → 0:1), then ethyl acetate/methanol (100:0 → 0:1) to yield eight fractions. Fraction 8 (57.8 mg) was purified by semi-preparative HPLC (column: NUCLEODUR Sphinx RP 250 × 10 mm), using acetonitrile/water (10:90 → 100:0) containing 0.1% formic acid in 30 min, to yield **1** (3.2 mg; RT = 14.32 min) and **6** (5.1 mg; RT = 13.63 min). By the same method, **3** (5.0 mg; RT = 16.53 min), **4** (3.9 mg; RT = 17.30 min), and **8** (4.5 mg; RT = 27.97 min) were obtained from fraction 7 (77.2 mg). Fraction 4 (63.8 mg) was subjected to semi-preparative HPLC, using a gradient of acetonitrile/water (15:85 → 45:55 in 25 min), containing 0.1% formic acid, to yield **2** (1.6 mg; RT = 20.26 min), **5** (5.8 mg; RT = 19.63 min), and **7** (4.0 mg; RT = 12.54 min). 

Compound **1** (deacetylfusarochromene): Yellow gum. [α]_D_^20^ −30.0 (*c* 0.3, MeOH). Molecular formula: C_15_H_20_N_2_O_3_. For ^1^H and ^13^C NMR data, see Table 1; HRESIMS *m*/*z* 277.1535 [M + H]^+^ (calcd for C_15_H_21_N_2_O_3_^+^ 277.1547).

Compound **2** (deacetamidofusarochrom-2′,3-diene): Yellow gum. Molecular formula: C_15_H_17_NO_3_. For ^1^H and ^13^C NMR data, see Table 1; HRESIMS *m*/*z* 260.1271 [M + H]^+^ (calcd for C_15_H_18_NO_3_^+^ 260.1281).

### 3.6. Kinase Inhibition Assays

The inhibitions of the kinase enzymatic activities were measured in 384-well plates using the ADP-GloTM assay kit (Promega, Madison, WI, USA) according to the recommendations of the manufacturer. This luminescent ADP detection assay is described in Zegzouti et al. [42]. The protocols used to measure the enzymatic activity of kinases analyzed in this study are described in Mokhtari Brikci-Nigassa et al. [43] except for human EGFR and EphB1 (both are recombinant, expressed by baculovirus in Sf9 insect cells). These kinases were assayed in kinase buffer “A” with 0.17 μg/μL of poly(L-glutamic acid-L-tyrosine) sodium salt as substrate. Kinase buffer “A”: 10 mM MgCl_2_, 1 mM EGTA, 1 mM DTT, 25 mM Tris-HCl pH 7.5, 50 μg.mL^−1^ heparin. Kinase reactions were carried out with either protein or peptide as substrate in the presence of 10 µM ATP. Peptide substrates were obtained from Proteogenix (Schiltigheim, France). The transmitted signal was measured using the Envision (PerkinElmer, Waltham, MA, USA) microplate luminometer and expressed in Relative Light Units (RLUs). To determine the half-maximal inhibitory concentration (IC_50_), the assays were performed in duplicate with increasing doses of the tested compounds. Kinase activities are expressed in % of maximal activity, i.e., measured without the tested compound but with a similar dose of DMSO. To validate each kinase assay, the following model inhibitors were used under the same conditions as the tested compounds: Barasertib (AZD1152-HQPA, #S1147, Selleckchem) for Aurora kinase B (AURKB); Staurosporine from *Streptomyces* sp. (#S5921, purity 95%, Sigma-Aldrich) for EphB1 and CK1ε; Indirubin-3′-oxime (#I0404, Sigma-Aldrich) for CDK5/p25, CDK9/CyclinT, GSK3β, *Rn*DYRK1A, and CLK1; CHR-6494 (#SML0648, Sigma-Aldrich) for HASPIN; Tofacitinib (CP-690550, #S2789, Selleckchem) for JAK3; Imatinib mesylate (STI571, #S1026, Selleckchem) for ABL1; SGI-1776 (#S2198, Selleckchem) for Pim1; Lenvatinib (#HY-10981, MedChemExpress) for VEGFR2; and Erlotinib (#HY-50896, MedChemExpress) for EGFR.

### 3.7. Cytotoxic Assay

#### 3.7.1. Cell Culture

HCT116, U-2 OS, and hTERT RPE-1 cells were cultured in Dulbecco’s modified Eagle’s medium (DMEM) supplemented with 10% fetal calf serum. Cells were cultured at 37 °C in a 5% CO_2_ humidified atmosphere.

#### 3.7.2. Cell Viability

Cells were grown in 96-well plates in the presence of a fixed concentration of 25 μM of each compound (for cell viability primary assessment) or increasing concentrations of each compound (from 50 to 0.01 µM) for 48 h (for EC_50_ determination). Cell viability was then assessed using the CellTiter 96^®^ AQueous One Solution Cell Proliferation Assay from Promega according to the manufacturer’s instructions. This colorimetric method used the MTS tetrazolium compound (3-(4,5-dimethylthiazol-2-yl)-5-(3-carboxymethoxyphenyl)-2-(4-sulfophenyl)-2H-tetrazolium) as a reagent to detect viable cells. Viability primary assessment was performed in duplicate, and EC_50_ experiments were performed in triplicate. EC_50_ values were determined from the dose–response curves using Prism GraphPad 9.5 software. A cell viability of 100% was calculated from a positive control (cells treated with 0.5% *v*/*v* DMSO). Staurosporine, an inducer of cell death, was used as a control compound. 

### 3.8. Antimicrobial Assay

MBC/MIC values of compounds **1** to **8** were determined using micro-broth dilution methods on *E. coli* ATCC25922, *S. enterica* CIP8297, *P. aeruginosa* ATCC27853, *S. aureus* ATCC25923, *E. faecalis* CIPA186, *B. cereus* ATCC6464, *L. monocytogenes* SOR100, and *C. albicans* ATCC2092. Experiments were performed as described in CLSI standard M07-A9: methods for dilution antimicrobial susceptibility tests for bacteria that grow aerobically. Briefly, wells of a 96-well microplate containing 1.0 × 10^6^ CFU/mL of each bacterial target were supplemented with a 2-fold serial dilution of each pure compound ranging from 250 μM to 0.12 μM. To define the bactericidal and bacteriostatic activities, 50 μL of every well that showed no growth was used to inoculate 3 mL of medium and incubated overnight at 37 °C under agitation. Each analysis was processed in triplicate.

## 4. Conclusions

Chemical investigation of the marine fungus *Fusarium equiseti* UBOCC-A-117302 led to the isolation of two new fusarochromanone derivatives, deacetylfusarochromene (**1**) and deacetamidofusarochrom-2′,3-diene (**2**), along with the previously reported metabolites fusarochromanone TDP-2 (**3**), fusarochromene (**4**), 2,2-dimethyl-5-amino-6-(2′*E*-ene-4′-hydroxylbutyryl)-4-chromone (**5**), fusarochromanone (**6**), (−)-chrysogine (**7**), and equisetin (**8**). All isolated compounds were evaluated for their protein kinase inhibitory activity, antibacterial properties, and cytotoxicity. Among them, **2** and **5** showed inhibition of three protein kinases, ABL1, JAK3, and EphB1, with IC_50_ values ranging from 1.42 to 25.48 μM. The unique side chain at C-6 appears to be crucial for the biological activities observed.

## Figures and Tables

**Table 1:** ^1^H (400 MHz) and ^13^C NMR (100 MHz) data of compounds **1** and **2** in CD_3_OD.

Position	1		2	
	^1^H [*δ*, mult. (*J* in Hz)]	^13^C (*δ*)	^1^H [*δ*, mult. (*J* in Hz)]	^13^C (*δ*)
2	-	77.6	-	77.5
3	5.65 d (10.0)	128.9	5.64 d (10.0)	128.8
4	6.58 d (10.0)	117.1	6.59 d (10.0)	117.3
5	-	150.2	-	150.6
6	-	112.8	-	113.9
7	7.61 d (9.0)	133.9	7.70 d (9.0)	134.5
8	6.12 d (9.0)	106.9	6.11 d (9.0)	106.6
9	-	159.9	-	159.7
10	-	107.6	-	107.6
11	1.40 s	28.0	1.40 s	28.0
12	1.40 s	28.0	1.41 s	28.0
1′	-	198.0	-	192.2
2′	3.36 m 3.21 dd (18.0, 8.3)	38.5	7.23 dt (15.2, 2.0)	126.1
3′	3.77 m	51.2	6.90 dt (15.2, 4.1)	145.6
4′	3.82 m 3.67 dd (10.7, 5.5)	62.7	4.33 dd (4.1, 2.2)	62.6

**Table 2:** Effects of compounds **1**–**8** on cell viability. Cells were incubated with increasing doses of each compound (up to 50 µM). Cell viability was measured after 48 h incubation by an MTS reduction assay as mentioned in the Materials and Methods. EC_50_ values (µM) were calculated from the dose–response curves.

Compound		EC_50_ (µM)	
	RPE-1	HCT-116	U2OS
**1**	0.176	0.087	0.896
**2**	10.030	13.730	13.180
**3**	23.140	62.950	35.090
**4**	16.700	84.380	39.790
**5**	5.222	8.036	7.351
**6**	0.058	0.170	0.232
**7**	>25	>25	>25
**8**	>25	>25	>25
Staurosporine	1.900	25.700	9.900

**Table 3:** Antimicrobial activity of compounds **2**, **4**, and **8**. Minimal inhibitory concentrations (MICs) and minimal bactericidal concentrations (MBCs) were determined according to the clinical and laboratory standards Institute (CLSI) guidelines M7-A09. * and ** indicate that a bactericidal or a bacteriostatic mode of action was highlighted, respectively. (−) indicates that no activity was visualized at the highest concentration (250 µM). The MIC values of erythromycin, used as a control, for *Listeria monocytogenes*, *Enterococcus faecalis*, and *Bacillus cereus* were 1.36 μM, 5.44 μM, and 5.45 μM, respectively.

	MBC/MIC (µM)			
Compound	2	4	8	Erythromycin
*L. monocytogenes* SOR 100	125*	125 *	31.25 **	1.36
*E. faecalis* CIP A 186	(−)	(−)	31.25 *	5.44
*B. cereus* ATCC 6464	(−)	(−)	7.8 **	5.45

## Data Availability

The original data presented in the study are included in the article/Supplementary Material; further inquiries can be directed to the corresponding author.

## Supplementary Materials

The following supporting information can be downloaded at https://www.mdpi.com/article/10.3390/md22100444/s1: Figure S1. UV spectra of **1**; Figure S2. HRESI(+)MS of **1**; Figure S3. ^1^H NMR (400 MHz) spectrum of **1** in CD_3_OD; Figure S4. ^13^C NMR (100 MHz) spectrum of **1** in CD_3_OD; Figure S5. ^1^H-^1^H COSY spectrum of **1** in CD_3_OD; Figure S6. ^1^H-^13^C HSQC spectrum of **1** in CD_3_OD; Figure S7. ^1^H-^13^C HMBC spectrum of **1** in CD_3_OD; Figure S8. UV spectra of **2**; Figure S9. HRESI(+)MS of **2**; Figure S10. ^1^H NMR (400 MHz) spectrum of **2** in CD_3_OD; Figure S11. ^13^C NMR (100 MHz) spectrum of **2** in CD_3_OD; Figure S12. ^1^H-^1^H COSY spectrum of **2** in CD_3_OD; Figure S13. ^1^H-^13^C HSQC spectrum of **2** in CD_3_OD; Figure S14. ^1^H-^13^C HMBC spectrum of **2** in CD_3_OD; Figure S15. HRESI(+)MS of **3**; Figure S16. ^1^H NMR (400 MHz) spectrum of **3** in CD_3_OD; Figure S17. ^13^C NMR (100 MHz) spectrum of **3** in CD_3_OD; Figure S18. ^1^H-^1^H COSY spectrum of **3** in CD_3_OD; Figure S19. ^1^H-^13^C HSQC spectrum of **3** in CD_3_OD; Figure S20. ^1^H-^13^C HMBC spectrum of **3** in CD_3_OD; Figure S21. HRESI(+)MS of **4**; Figure S22. ^1^H NMR (400 MHz) spectrum of **4** in CD_3_OD; Figure S23. ^13^C NMR (100 MHz) spectrum of **4** in CD_3_OD; Figure S24. ^1^H-^1^H COSY spectrum of **4** in CD_3_OD; Figure S25. ^1^H-^13^C HSQC spectrum of **4** in CD_3_OD; Figure S26. ^1^H-^13^C HMBC spectrum of **4** in CD_3_OD; Figure S27. HRESI(+)MS of **5**; Figure S28. ^1^H NMR (400 MHz) spectrum of **5** in CD_3_OD; Figure S29. ^13^C NMR (100 MHz) spectrum of **5** in CD_3_OD; Figure S30. ^1^H-^1^H COSY spectrum of **5** in CD_3_OD; Figure S31. ^1^H-^13^C HSQC spectrum of **5** in CD_3_OD; Figure S32. ^1^H-^13^C HMBC spectrum of **5** in CD_3_OD; Figure S33. HRESI(+)MS of **6**; Figure S34. ^1^H NMR (400 MHz) spectrum of **6** in CD_3_OD; Figure S35. ^13^C NMR (100 MHz) spectrum of 6 in CD_3_OD; Figure S36. ^1^H-^1^H COSY spectrum of **6** in CD_3_OD; Figure S37. ^1^H-^13^C HSQC spectrum of **6** in CD_3_OD; Figure S38. ^1^H-^13^C HMBC spectrum of **6** in CD_3_OD; Figure S39. HRESI(+)MS of **7**; Figure S40. ^1^H NMR (400 MHz) spectrum of **7** in CD_3_OD; Figure S41. ^13^C NMR (100 MHz) spectrum of **7** in CD_3_OD; Figure S42. ^1^H-^13^C HSQC spectrum of **7** in CD_3_OD; Figure S43. ^1^H-^13^C HMBC spectrum of **7** in CD_3_OD; Figure S44. HRESI(+)MS of **8**; Figure S45. ^1^H NMR (400 MHz) spectrum of **8** in CDCl_3_; Figure S46. ^13^C NMR (100 MHz) spectrum of **8** in CDCl_3_; Figure S47. ^1^H-^13^C HSQC spectrum of **8** in CDCl_3_; Figure S48. ^1^H-^13^C HMBC spectrum of **8** in CDCl_3_; Figure S49. ^1^H-^1^H COSY spectrum of **8** in CDCl_3_; Figure S50. ^1^H-^1^H NOESY spectrum of **8** in CDCl_3_; Table S1. Primary screening of compounds **1**–**8** against a set of 14 disease-related protein kinases. The % of remaining kinase activities are reported in the table; Figure S51. IC_5_₀ (µM) for compounds **2** and **5** against the selected protein kinases; Table S2. Primary screening of compounds **1**–**8** against RPE-1, HCT-116, and U2OS cell lines.
