# Supplementary material for: New Fusarochromanone Derivatives from the Marine Fungus Fusarium equiseti UBOCC-A-117302
Source: Mar Drugs. 2024 Sep 28;22(10):444. doi: 10.3390/md22100444 (PMC11509758; doi:10.3390/md22100444)
Supplement: Supplementary file 1 [file marinedrugs-22-00444-s001.zip › marinedrugs-3203455-supplementary.pdf]

Supporting Information

New Fusarochromanone Derivatives from the Marine Fungus *Fusarium equiseti* UBOCC-A-117302

Giang Nam Pham [1], Béatrice Josselin [2,3], Arnaud Cousseau [1,2], Blandine Baratte [2,3], Marie Dayras [1], Christophe Le Meur [4], Stella Debaets [4], Amélie Weill [4], Thomas Robert [2,3], Gaëtan Burgaud [4], Ian Probert [5], Fatouma Mohamed Abdoul-Latif [6], Laurent Boyer [7], Stéphane Bach [2,3]* and Mohamed Mehiri [1,]*

[1] Marine Natural Products Team, Institut de Chimie de Nice, Université Côte d'Azur, CNRS, UMR 7272, 06108 Nice, France; giangnampham94@gmail.com (G.N.P.); arnaud.cousseau@outlook.com (A.C.)

[2] Integrative Biology of Marine Models Laboratory (LBI2M), Station Biologique de Roscoff, Sorbonne Université, CNRS, UMR 8227, 29680 Roscoff, France; baratte@sb-roscoff.fr (B.B.)

[3] Plateforme de Criblage KISSf (Kinase Inhibitor Specialized Screening Facility), Station Biologique de Roscoff, Sorbonne Université, CNRS, FR2424, 29680 Roscoff, France

[4] Laboratoire Universitaire de Biodiversité et Écologie Microbienne, Université de Brest, INRAE, 29280 Plouzané, France

[5] Roscoff Culture Collection, Station Biologique de Roscoff, Sorbonne Université, CNRS, FR2424, 29680 Roscoff, France

[6] Medicinal Research Institute, Center for Studies and Research of Djibouti, IRM-CERD, Route de l'Aéroport, Haramous, Djibouti City P.O. Box 486, Djibouti; fatouma_abdoulatif@yahoo.fr

[7] INSERM U1065, Centre Méditerranéen de Médecine Moléculaire (C3M), Bâtiment Universitaire ARCHIMED, 151 Route de Saint Antoine de Ginestière BP, 23194 Nice, France

* Correspondence: bach@sb-roscoff.fr (S.B.); mohamed.mehiri@univ-cotedazur.fr (M.M.)

Contents

Figure S1. UV spectra of **1** 4

Figure S2. HRESI(+)MS of **1** 5

Figure S3. ^{1}H NMR (400 MHz) spectrum of **1** in CD_3OD 6

Figure S4. ^{13}C NMR (100 MHz) spectrum of **1** in CD_3OD 7

Figure S5. ^{1}H-^{1}H COSY spectrum of **1** in CD_3OD 8

Figure S6. ^{1}H-^{13}C HSQC spectrum of **1** in CD_3OD 9

Figure S7. ^{1}H-^{13}C HMBC spectrum of **1** in CD_3OD 10

Figure S8. UV spectra of **2** 11

Figure S9. HRESI(+)MS of **2** 12

Figure S10. ^{1}H NMR (400 MHz) spectrum of **2** in CD_3OD 13

Figure S11. ^{13}C NMR (100 MHz) spectrum of **2** in CD_3OD 14

Figure S12. ^{1}H-^{1}H COSY spectrum of **2** in CD_3OD 15
Figure S13. ^{1}H-^{13}C HSQC spectrum of **2** in CD_3OD 16
Figure S14. ^{1}H-^{13}C HMBC spectrum of **2** in CD_3OD 17
Figure S15. HRESI(+)MS of **3** 19
Figure S16. ^{1}H NMR (400 MHz) spectrum of **3** in CD_3OD 20
Figure S17. ^{13}C NMR (100 MHz) spectrum of **3** in CD_3OD 21
Figure S18. ^{1}H-^{1}H COSY spectrum of **3** in CD_3OD 22
Figure S19. ^{1}H-^{13}C HSQC spectrum of **3** in CD_3OD 23
Figure S20. ^{1}H-^{13}C HMBC spectrum of **3** in CD_3OD 24
Figure S21. HRESI(+)MS of **4** 25
Figure S22. ^{1}H NMR (400 MHz) spectrum of **4** in CD_3OD 26
Figure S23. ^{13}C NMR (100 MHz) spectrum of **4** in CD_3OD 27
Figure S24. ^{1}H-^{1}H COSY spectrum of **4** in CD_3OD 28
Figure S25. ^{1}H-^{13}C HSQC spectrum of **4** in CD_3OD 29
Figure S26. ^{1}H-^{13}C HMBC spectrum of **4** in CD_3OD 30
Figure S27. HRESI(+)MS of **5** 31
Figure S28. ^{1}H NMR (400 MHz) spectrum of **5** in CD_3OD 32
Figure S29. ^{13}C NMR (100 MHz) spectrum of **5** in CD_3OD 33
Figure S30. ^{1}H-^{1}H COSY spectrum of **5** in CD_3OD 34
Figure S31. ^{1}H-^{13}C HSQC spectrum of **5** in CD_3OD 35
Figure S32. ^{1}H-^{13}C HMBC spectrum of **5** in CD_3OD 36
Figure S33. HRESI(+)MS of **6** 37
Figure S34. ^{1}H NMR (400 MHz) spectrum of **6** in CD_3OD 38
Figure S35. ^{13}C NMR (100 MHz) spectrum of **6** in CD_3OD 39
Figure S36. ^{1}H-^{1}H COSY spectrum of **6** in CD_3OD 40
Figure S37. ^{1}H-^{13}C HSQC spectrum of **6** in CD_3OD 41
Figure S38. ^{1}H-^{13}C HMBC spectrum of **6** in CD_3OD 42

Figure S39. HRESI(+)MS of **7** 43

Figure S40. ^{1}H NMR (400 MHz) spectrum of **7** in CD_3OD 44

Figure S41. ^{13}C NMR (100 MHz) spectrum of **7** in CD_3OD 45

Figure S42. ^{1}H-^{13}C HSQC spectrum of **7** in CD_3OD 46

Figure S43. ^{1}H-^{13}C HMBC spectrum of **7** in CD_3OD 47

Figure S44. HRESI(+)MS of **8** 48

Figure S45. ^{1}H NMR (400 MHz) spectrum of **8** in $CDCl_3$ 49

Figure S46. ^{13}C NMR (100 MHz) spectrum of **8** in $CDCl_3$ 50

Figure S47. ^{1}H-^{13}C HSQC spectrum of **8** in $CDCl_3$ 51

Figure S48. ^{1}H-^{13}C HMBC spectrum of **8** in $CDCl_3$ 52

Figure S49. ^{1}H-^{1}H COSY spectrum of **8** in $CDCl_3$ 53

Figure S50. ^{1}H-^{1}H NOESY spectrum of **8** in $CDCl_3$ 54

Table S1. Primary screening of compounds **1**-**8** against a set of 14 disease-related protein kinases. The % of remaining kinase activities are reported on the table. 55

Figure S51. IC_{50} (μM) for compounds **2** and **5** against the selected protein kinases 57

Table S2. Primary screening of compounds **1**-**8** against RPE-1, HCT-116, and U2OS cell lines 58

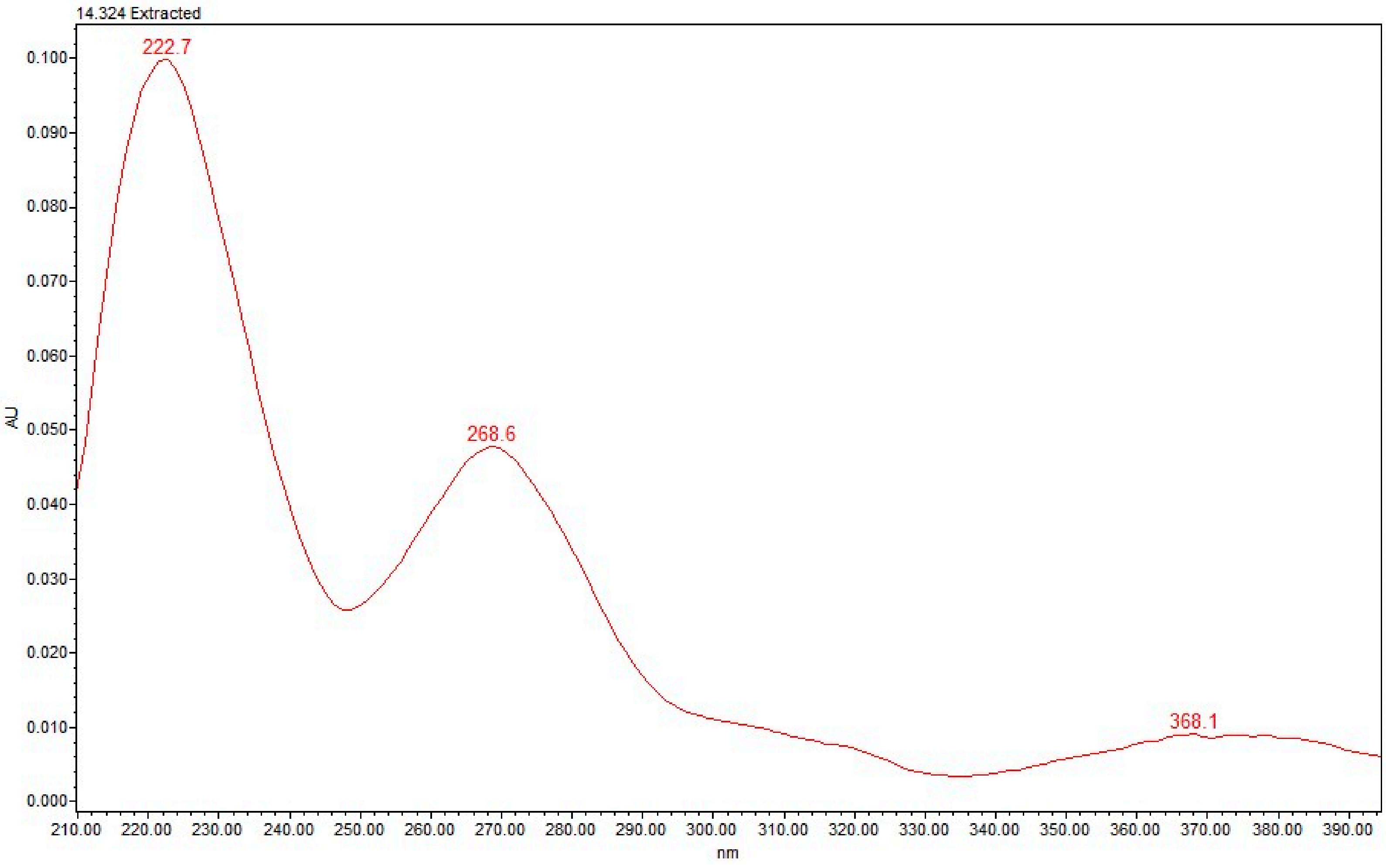

Figure S1. UV spectra of 1

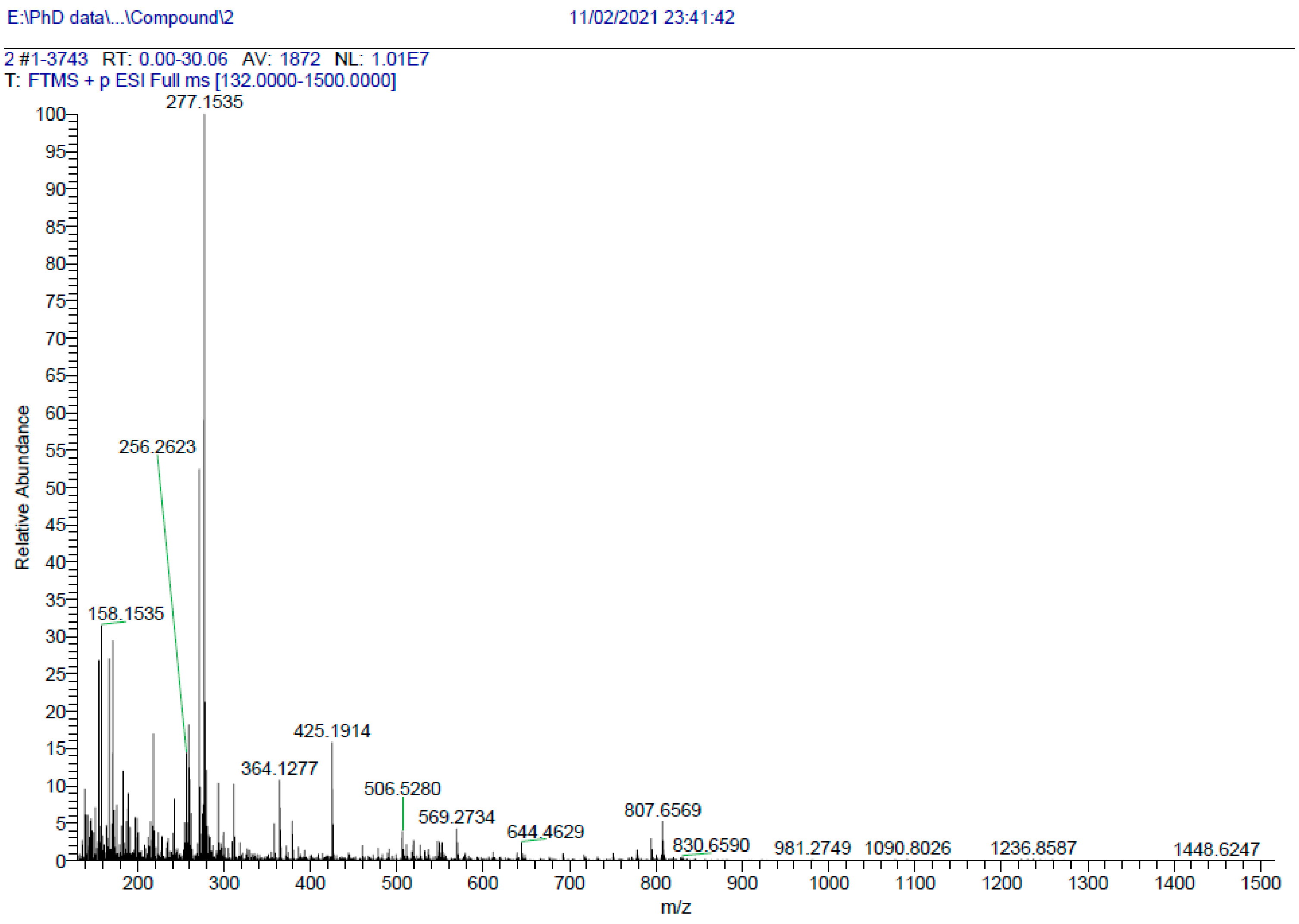

Figure S2. HRESI(+)MS of **1**

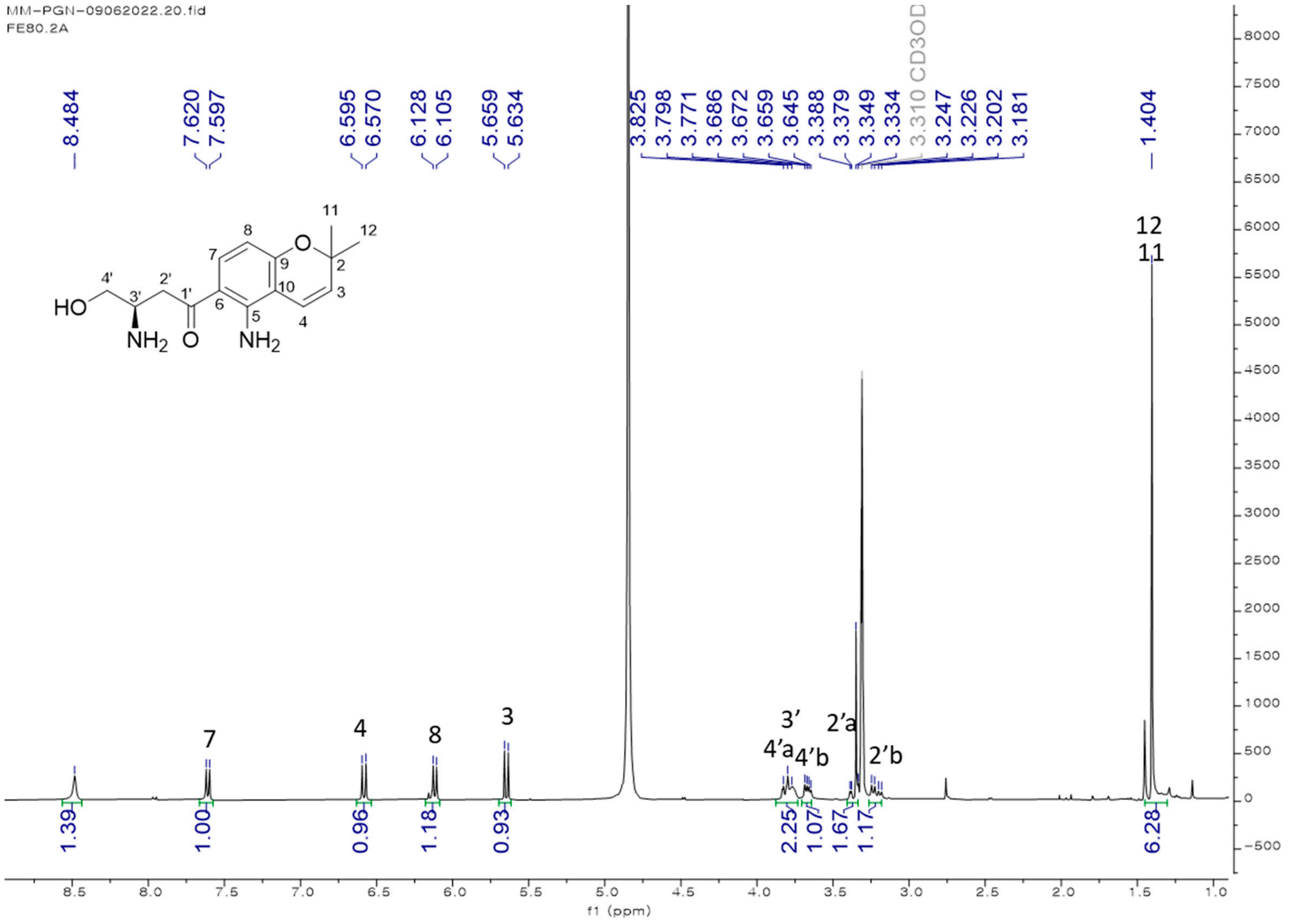

Figure S3. ^{1}H NMR (400 MHz) spectrum of **1** in CD_3OD

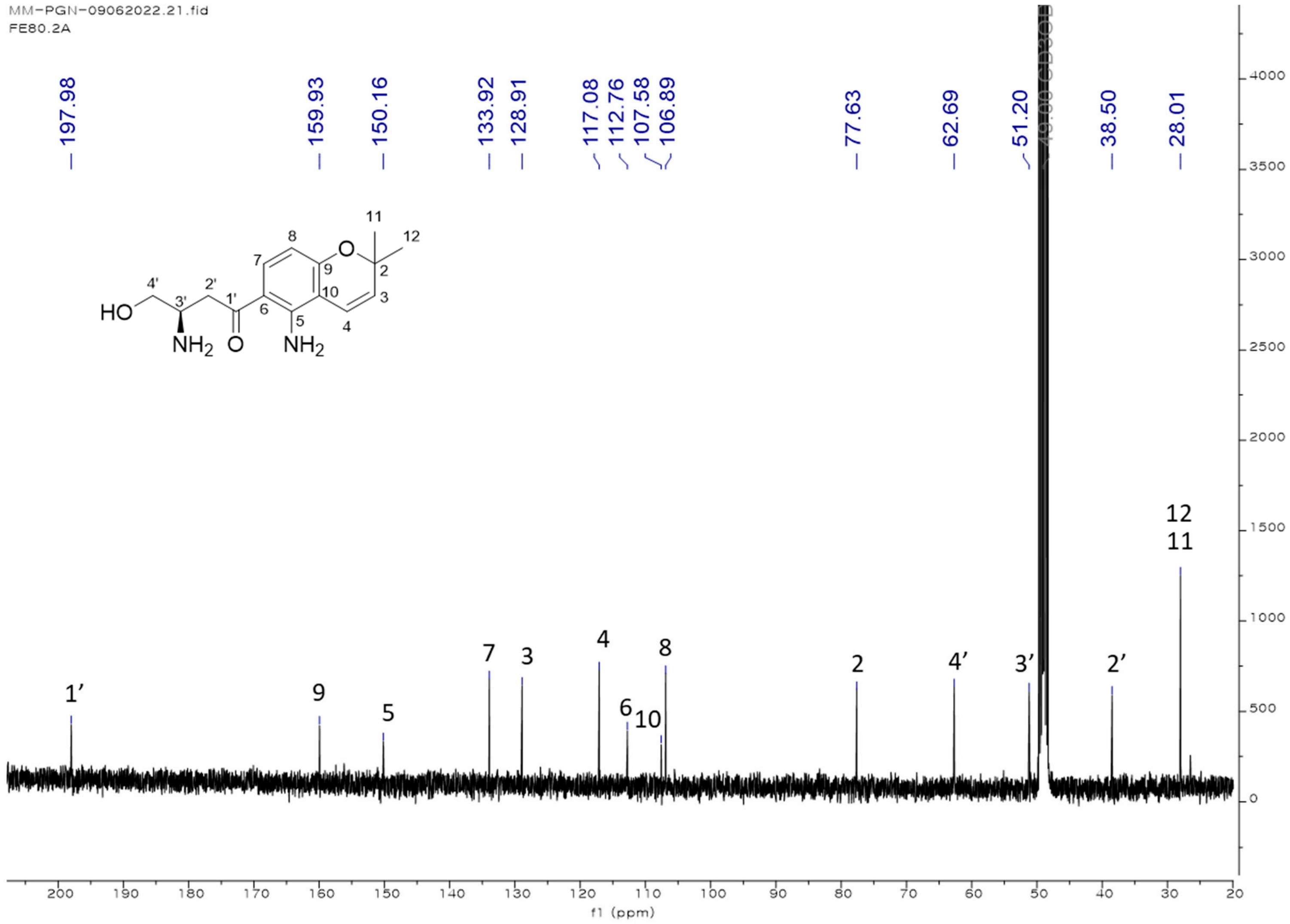

Figure S4. ^{13}C NMR (100 MHz) spectrum of **1** in CD_3OD

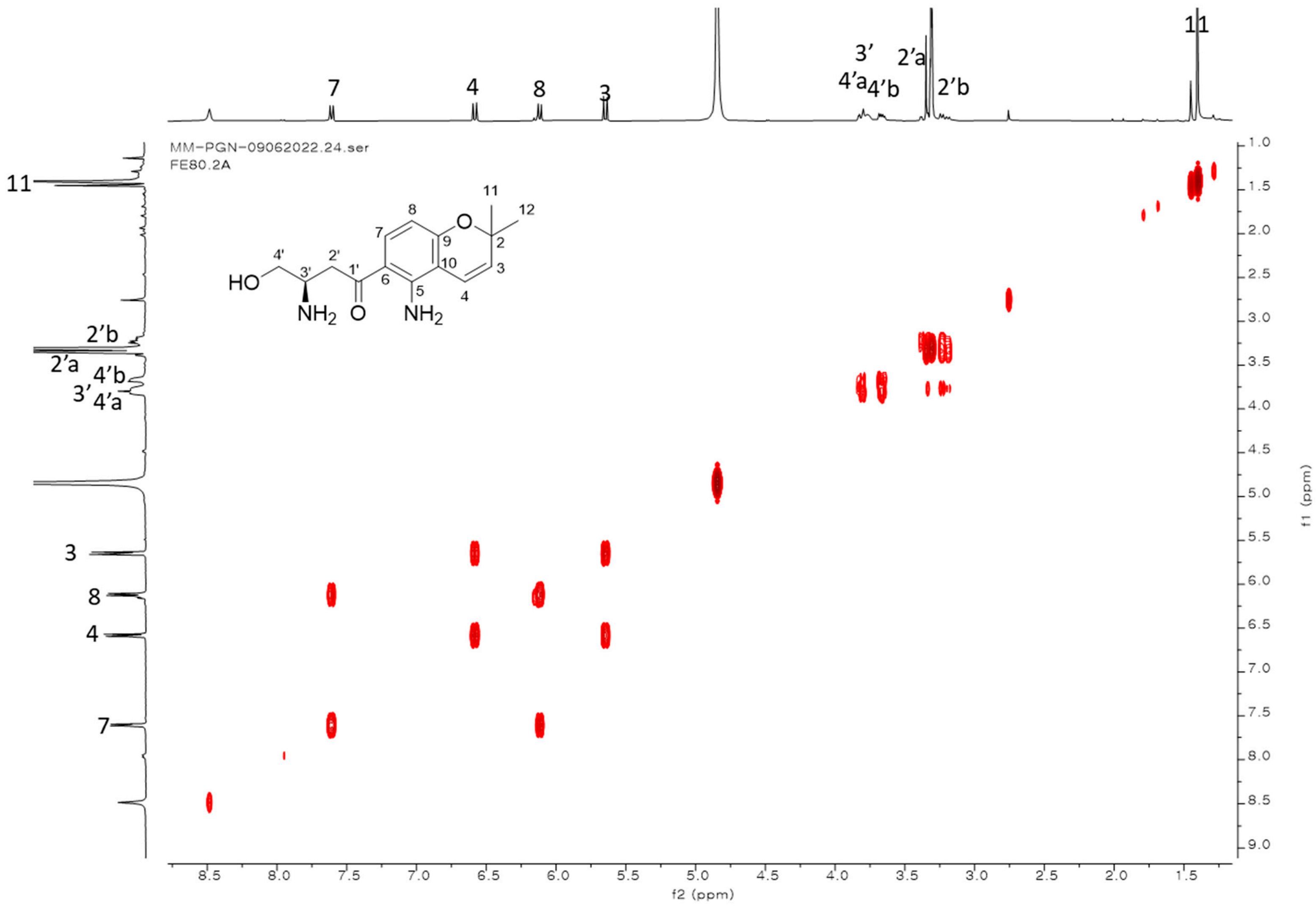

Figure S5. ^{1}H-^{1}H COSY spectrum of **1** in CD_3OD

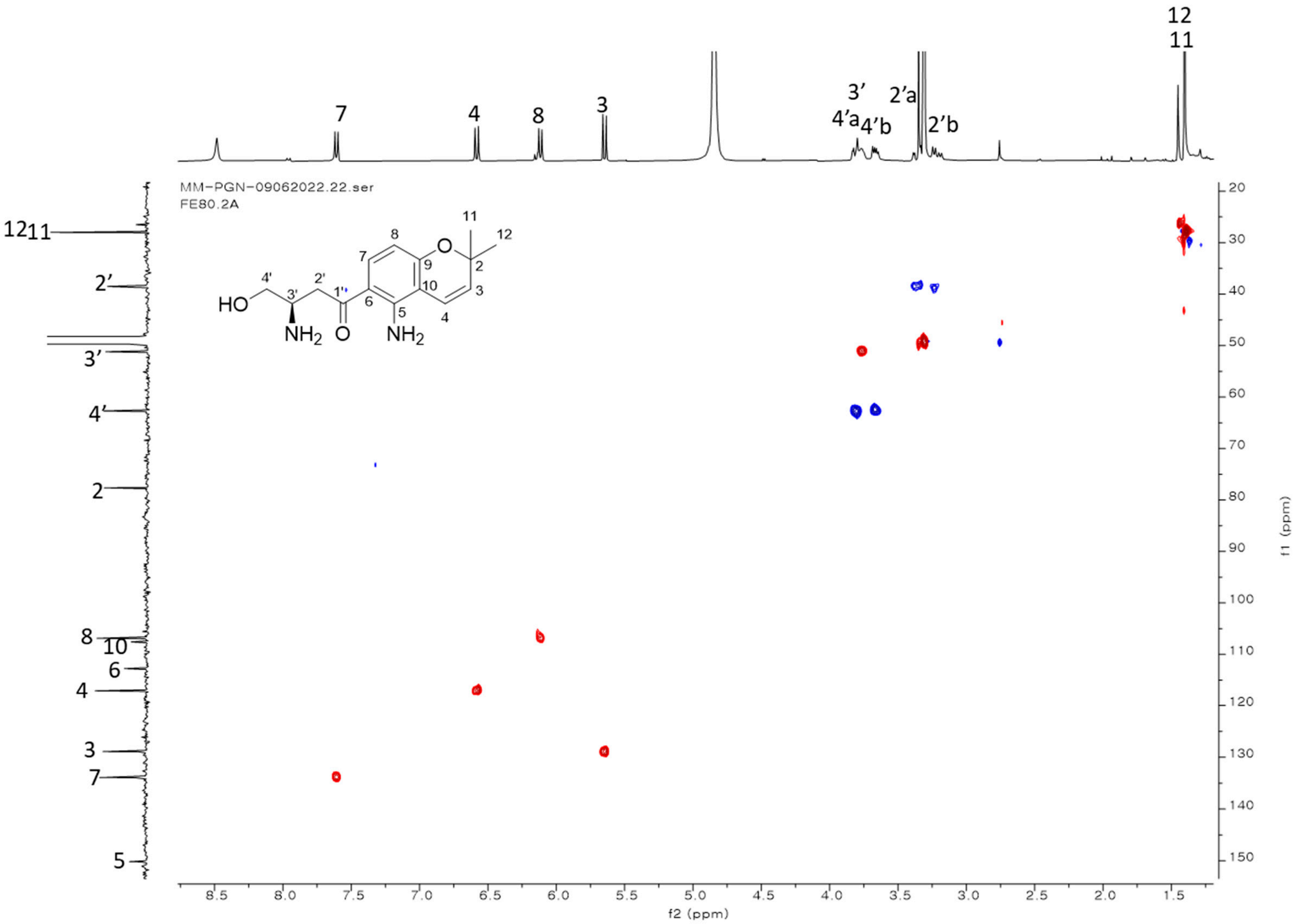

Figure S6. ^{1}H-^{13}C HSQC spectrum of **1** in CD_3OD

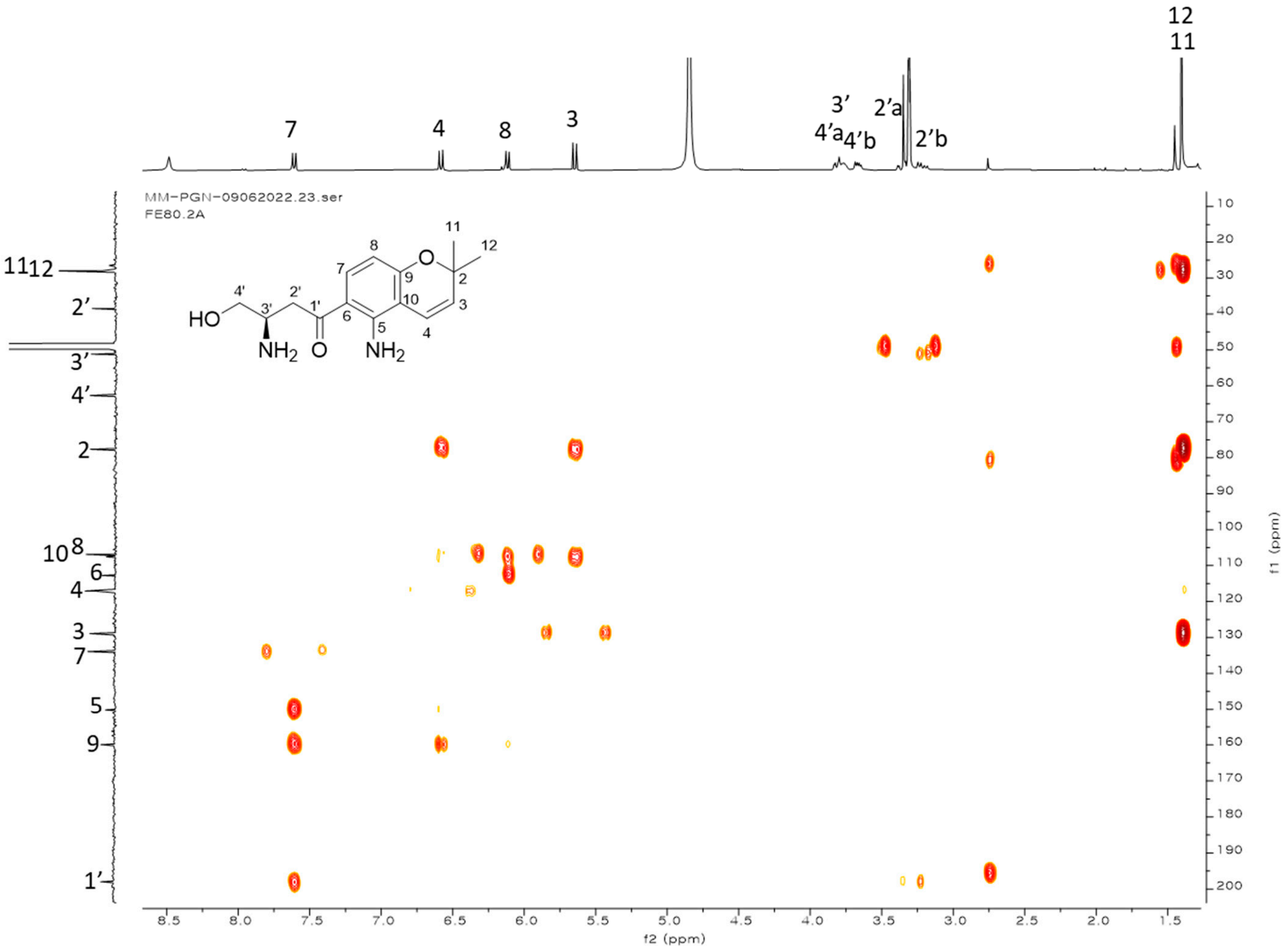

Figure S7. ^{1}H-^{13}C HMBC spectrum of **1** in CD_3OD

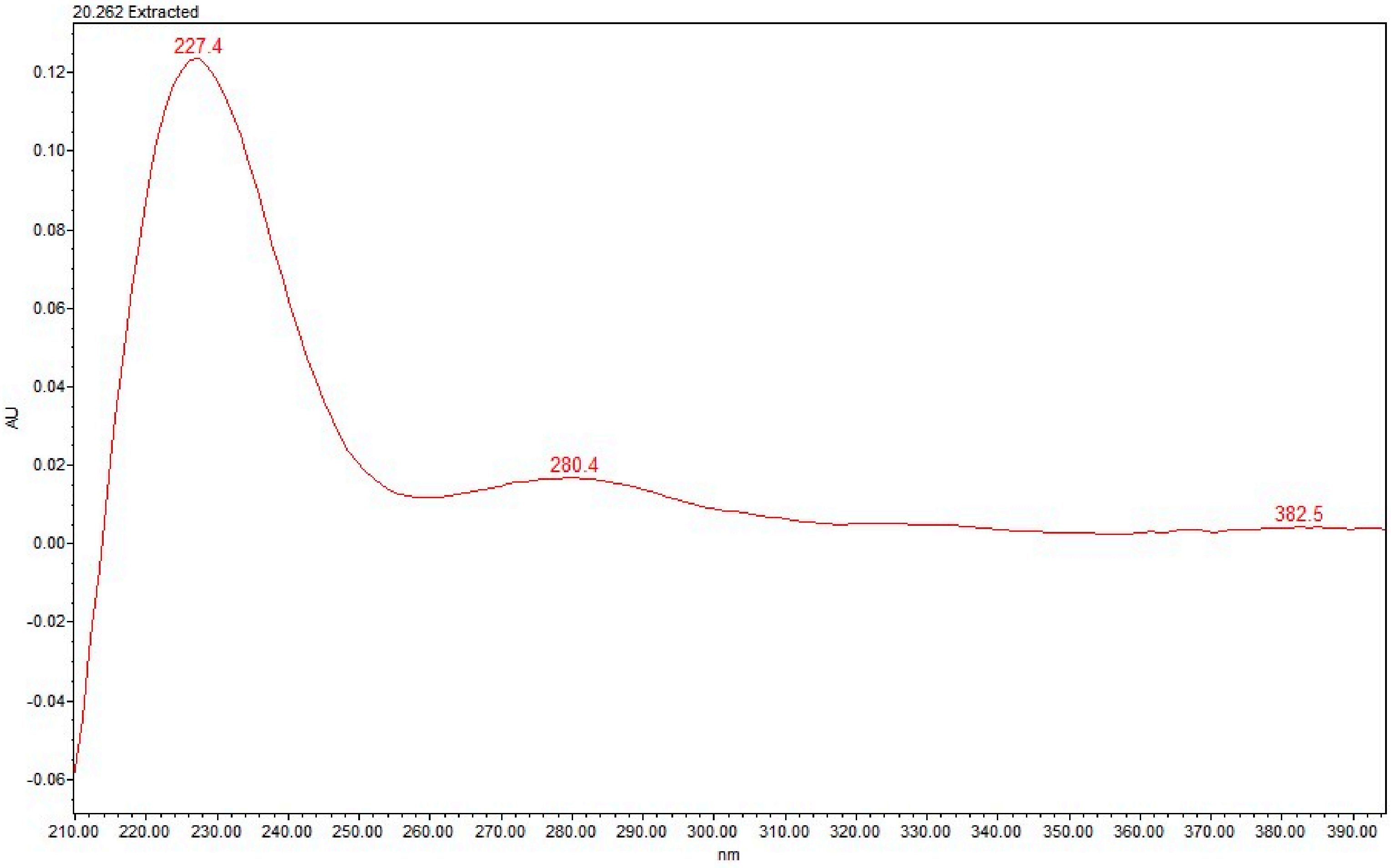

Figure S8. UV spectra of **2**

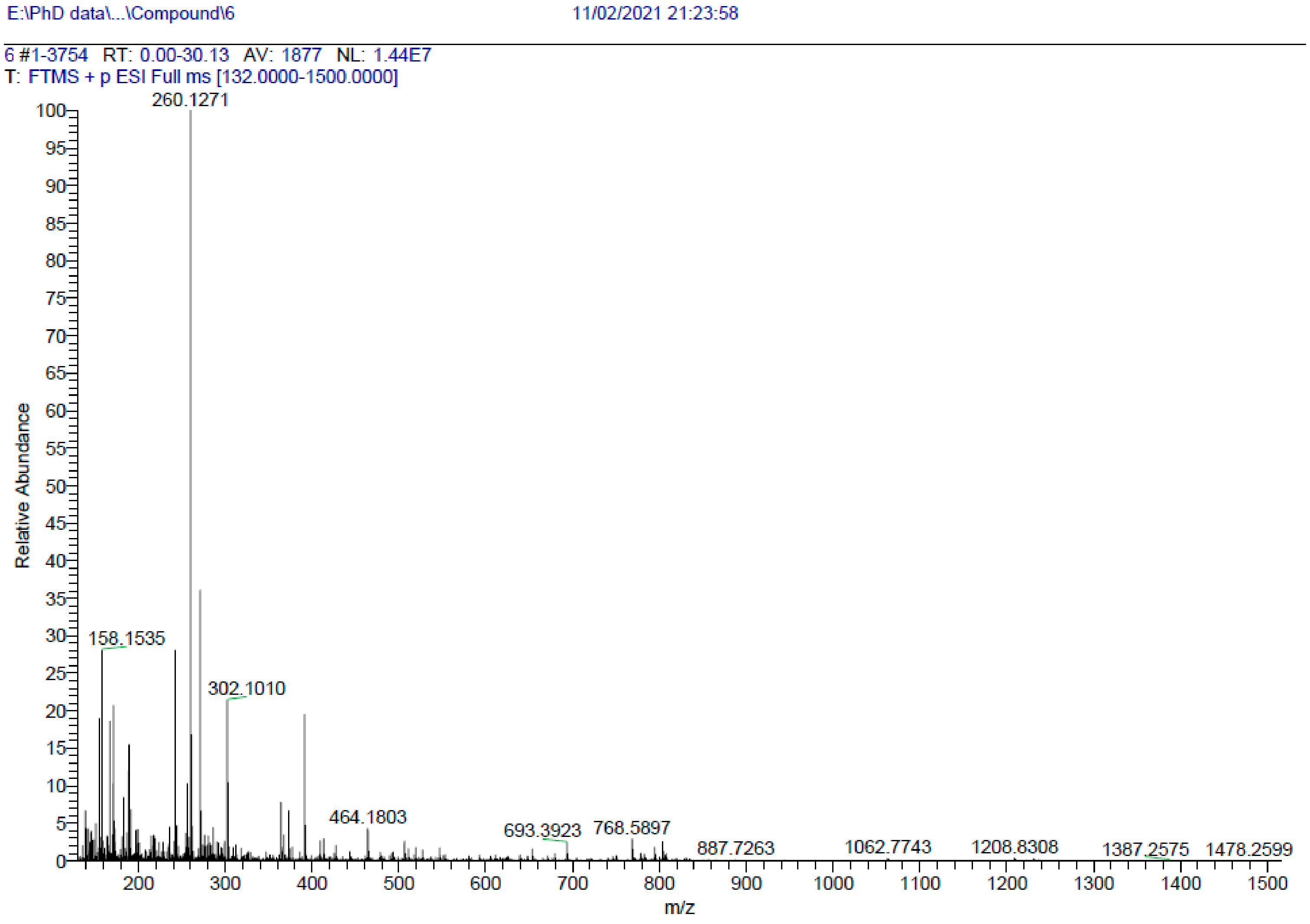

Figure S9. HRESI(+)MS of **2**

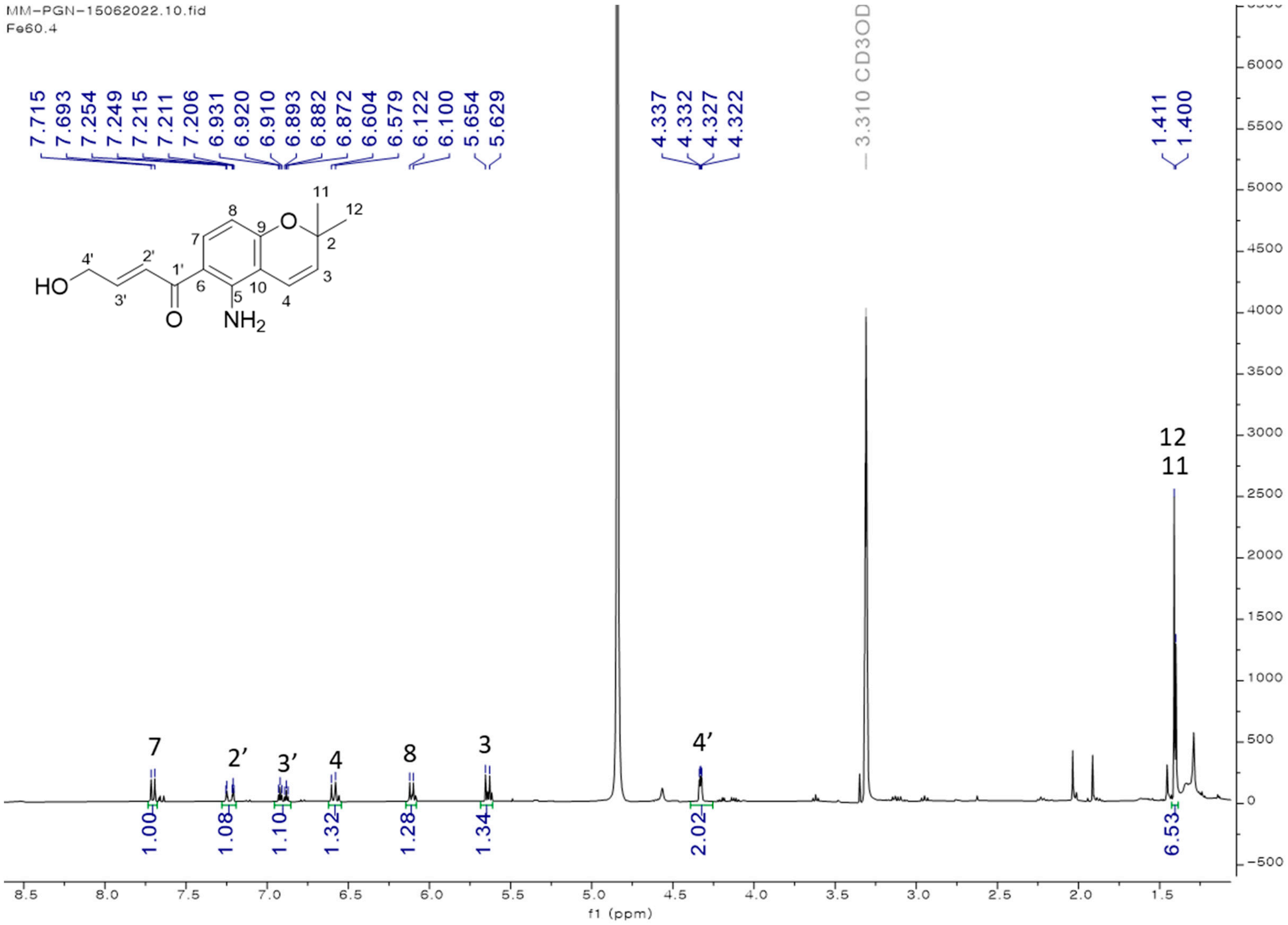

Figure S10. ^{1}H NMR (400 MHz) spectrum of **2** in CD_3OD

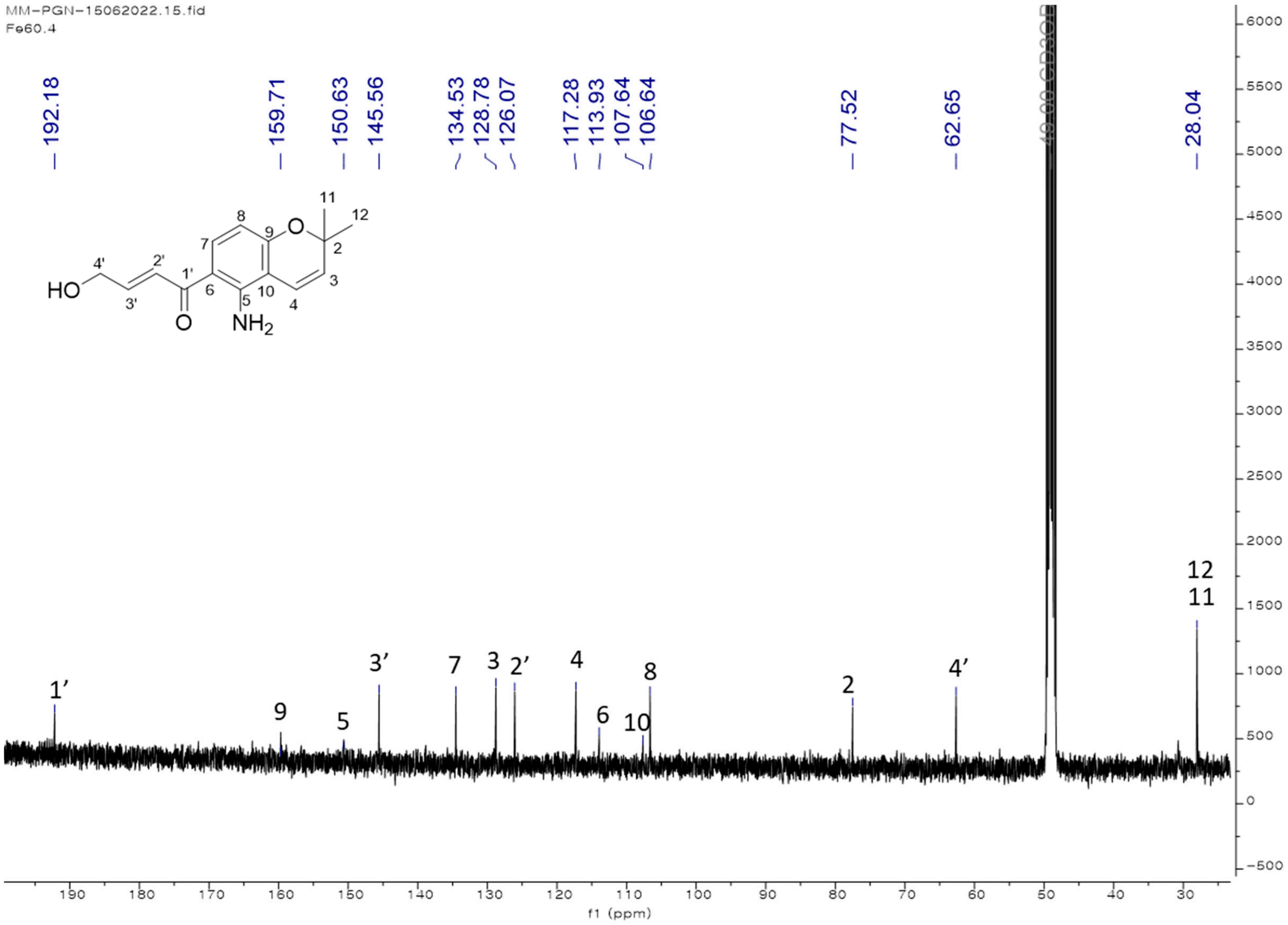

Figure S11. ^{13}C NMR (100 MHz) spectrum of **2** in CD_3OD

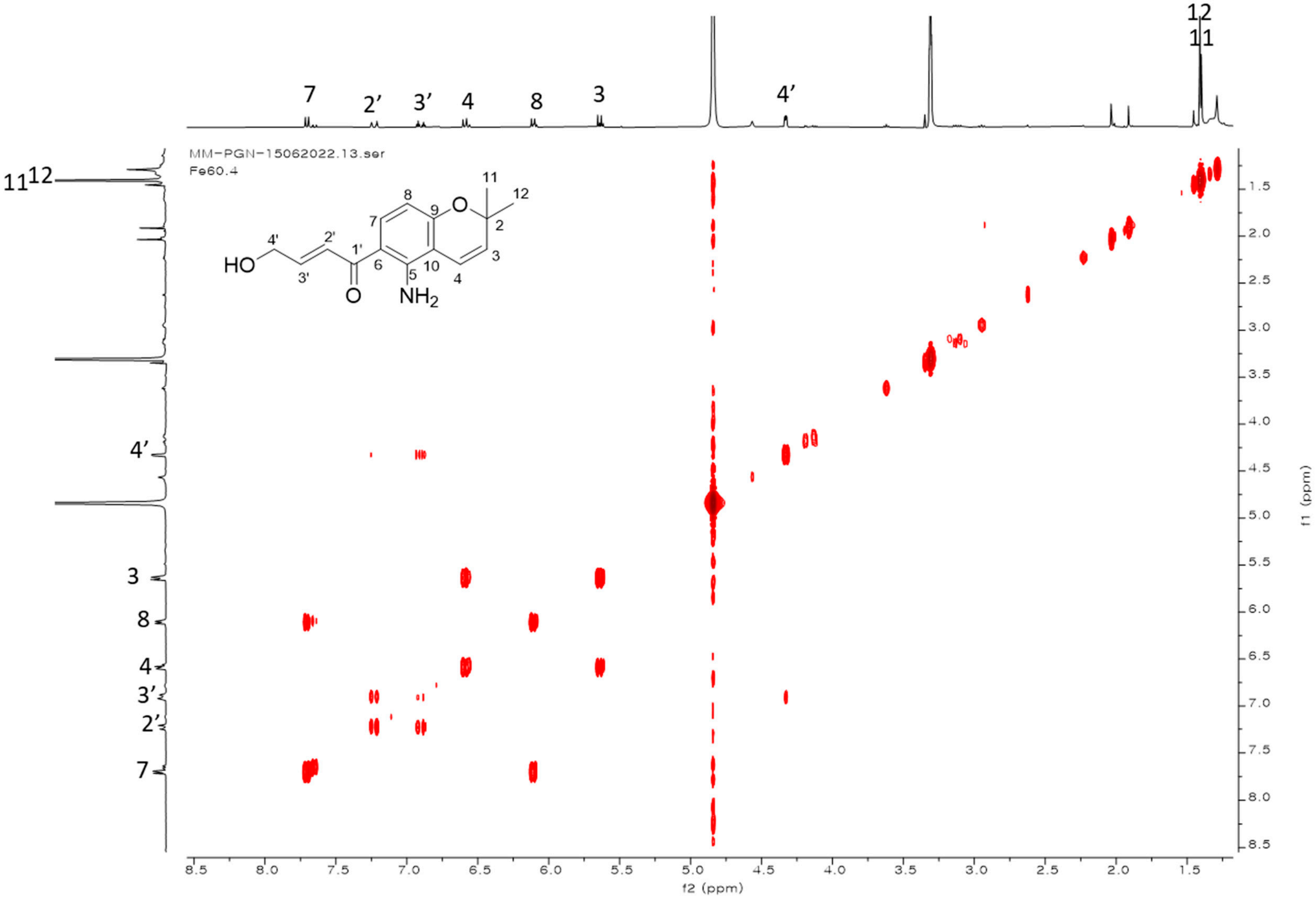

Figure S12. ^{1}H-^{1}H COSY spectrum of **2** in CD_3OD

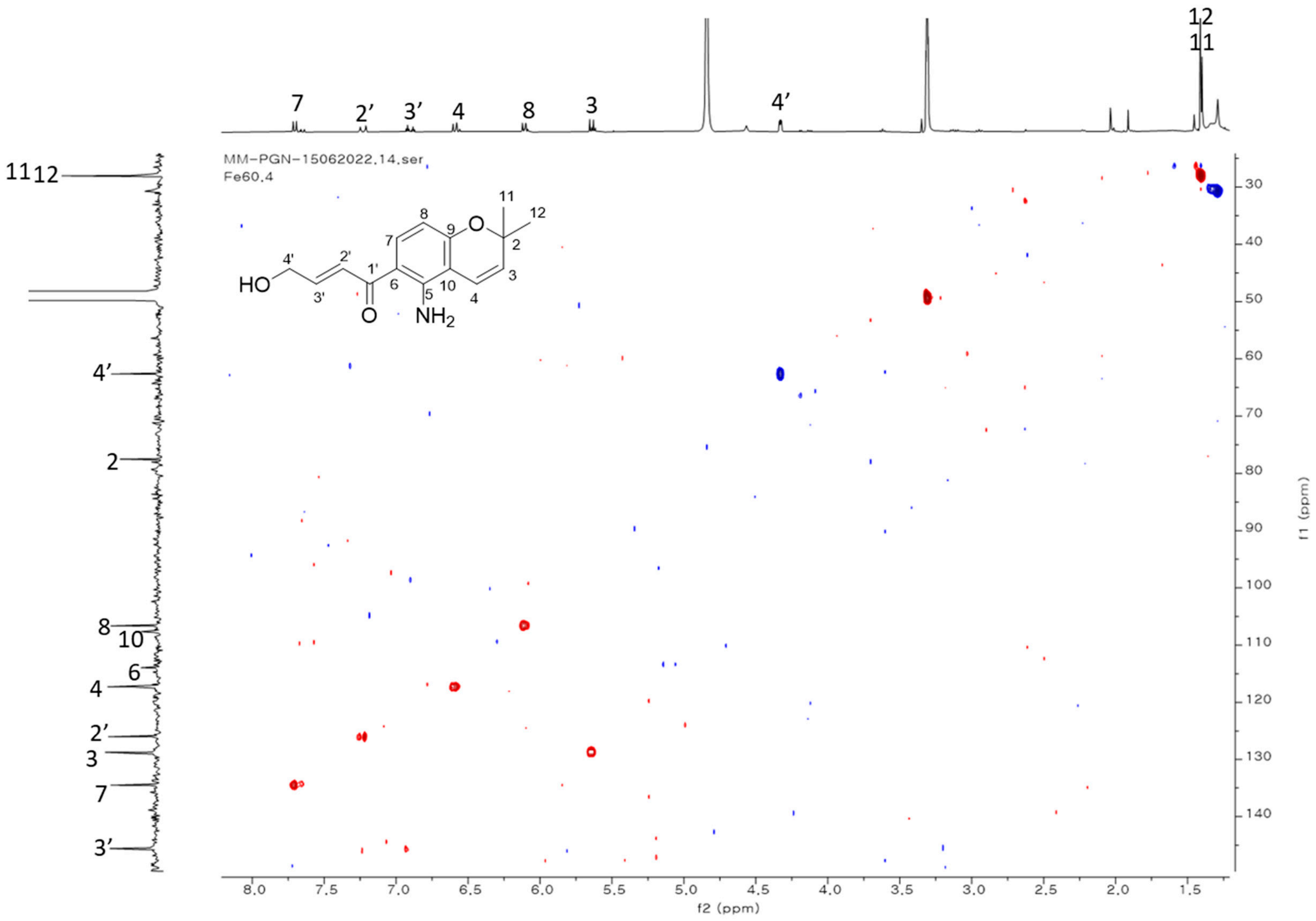

Figure S13. ^1H-^{13}C HSQC spectrum of **2** in CD_3OD

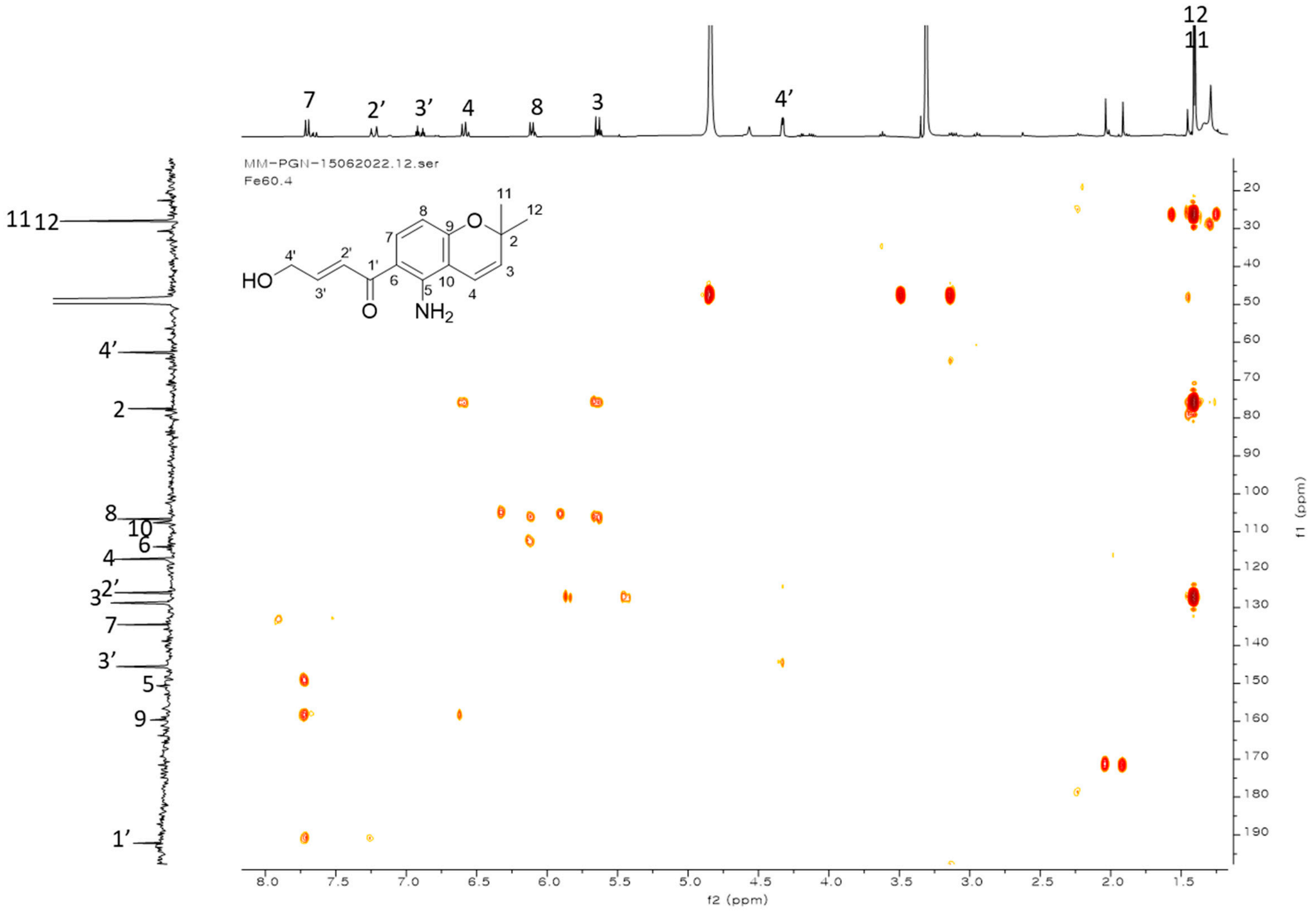

Figure S14. ^{1}H-^{13}C HMBC spectrum of **2** in CD_3OD

Compound **3** (fusarochromanone TDP-2): Yellow gum. $[\alpha]_D^{20}$ -86.0 (*c* 0.1, MeOH). Molecular formula: $C_{17}H_{22}N_2O_5$. ^{1}H NMR (400 MHz, CD_3OD) δ_H 8.02 (1H, d, *J* = 9.0 Hz, H-7), 6.10 (1H, d, *J* = 9.0 Hz, H-8), 4.37 (1H, m, H-3′), 3.61 (1H, m, H-4′), 3.12 (1H, dd, *J* = 15.8, 6.1 Hz, H-2′a), 3.06 (1H, dd, *J* = 15.8, 7.2 Hz, H-2′b), 2.73 (1H, s, H-3), 1.91 (1H, s, H-8′), 1.44 (2H, s, H-11, H-12). ^{13}C NMR (100 MHz, CD_3OD) δ_C 199.6 (C-1′), 195.6 (C-4), 173.1 (C-7′), 167.4 (C-9), 155.8 (C-5), 141.9 (C-7), 112.8 (C-6), 105.5 (C-8), 105.0 (C-10), 80.6 (C-2), 64.4 (C-4′), 50.3 (C-3′), 49.6 (C-3), 40.9 (C-2′), 26.5 (C-11, C-12), 22.7 (C-8′). HRESIMS *m/z* 335.1585 [M + H]+ (calcd for $C_{17}H_{23}N_2O_5^+$ 335.1601).
Compound **4** (fusarochromene): Yellow gum. $[\alpha]_D^{20}$ -14.0 (*c* 0.1, MeOH). Molecular formula: $C_{17}H_{22}N_2O_4$. ^{1}H NMR (400 MHz, CD_3OD) δ_H 7.69 (1H, d, *J* = 9.0 Hz, H-7), 6.57 (1H, d, *J* = 10.0 Hz, H-4), 6.09 (1H, d, *J* = 9.0 Hz, H-8), 5.62 (1H, d, *J* = 10.0 Hz, H-3), 4.39 (1H, tt, *J* = 5.80 Hz, H-3′), 3.60 (1H, m, H-4′), 3.12 (1H, m, H-2′a), 3.06 (1H, m, H-2′b), 1.91 (1H, s, H-8′), 1.40 (2H, s, H-11, H-12). ^{13}C NMR (100 MHz, CD_3OD) δ_C 200.1 (C-1′), 173.0 (-CONH-), 159.5 (C-9), 149.9 (C-5), 134.5 (C-7), 128.7 (C-3), 117.3 (C-4), 113.6 (C-6), 107.6 (C-10), 106.5 (C-8), 77.4 (C-2), 64.4 (C-4′), 50.4 (C-3′), 41.0 (C-2′), 28.0 (C-11, C-12), 22.7 (CH_3CO-). HRESIMS *m/z* 319.1637 $[M + H]^+$ (calcd for $C_{17}H_{23}N_2O_4$+ 319.1652).
Compound **5** (2,2-dimethyl-5-amino-6-(2′*E*-ene-4′-hydroxylbutyryl)-4-chromone): Yellow gum. Molecular formula: $C_{15}H_{17}NO_4$. ^{1}H NMR (400 MHz, CD_3OD) δ_H 8.05 (1H, d, *J* = 9.0 Hz, H-7), 7.22 (1H, d, *J* = 15.2 Hz, H-2′), 6.94 (1H, dt, *J* = 15.2; 4.0 Hz, H-3′), 6.12 (1H, d, *J* = 9.0 Hz, H-8), 4.33 (1H, dd, *J* = 3.9; 2.0 Hz, H-4′), 2.75 (1H, s, H-3), 1.45 (2H, s, H-11, H-12). ^{13}C NMR (100 MHz, CD_3OD) δ_C 195.6 (C-1′), 191.5 (C-4), 167.5 (C-9), 156.5 (C-5), 146.2 (C-3′), 141.8 (C-7), 125.4 (C-2′), 113.1 (C-6), 105.5 (C-10), 105.2 (C-8), 80.6 (C-2), 62.6 (C-4′), 49.6 (C-3), 26.6 (C-11, C-12). HRESIMS *m/z* 276.1221 $[M + H]^+$ (calcd for $C_{15}H_{18}NO_4^+$ 276.1230).
Compound **6** (fusarochromanone): Yellow gum. $[\alpha]_D^{20}$ -212.0 (*c* 0.1, MeOH). Molecular formula: $C_{15}H_{20}N_2O_4$. ^{1}H NMR (400 MHz, CD_3OD) δ_H 7.96 (1H, d, *J* = 9.0 Hz, H-7), 6.14 (1H, d, *J* = 9.0 Hz, H-8), 3.80 (1H, m, H-4′a), 3.77 (1H, m, H-3′), 3.67 (1H, dd, *J* = 10.4, 5.2 Hz, H-4′b), 3.36 (1H, m, H-2′a), 3.22 (1H, dd, *J* = 17.9, 8.0 Hz, H-2′b), 2.76 (1H, s, H-3), 1.45 (2H, s, H-11, H-12). ^{13}C NMR (100 MHz, CD_3OD) δ_C 197.8 (C-1′), 195.6 (C-4), 167.9 (C-9), 155.8 (C-5), 141.4 (C-7), 112.1 (C-6), 105.5 (C-8), 105.4 (C-10), 80.8 (C-2), 62.7 (C-4′), 50.9 (C-3′), 49.6 (C-3), 38.6 (C-2′), 26.5 (C-11, C-12). HRESIMS *m/z* 293.1493 $[M + H]^+$ (calcd for $C_{15}H_{21}N_2O_4^+$ 293.1496).
Compound **7** ((-)-chrysogine): Amorphous powders. $[\alpha]_D^{20}$ -20.0 (*c* 0.1, MeOH). Molecular formula: $C_{10}H_{10}N_2O_2$. ^{1}H NMR (400 MHz, CD_3OD) δ_H 8.20 (1H, dd, *J* = 8.0, 1.5 Hz, H-6), 7.82 (1H, ddd, *J* = 8.5, 7.1, 1.5 Hz, H-5), 7.69 (1H, dd, *J* = 8.2, 1.2 Hz, H-3), 7.52 (1H, dd, *J* = 8.2, 7.1, 1.2 Hz, H-4), 4.74 (1H, q, *J* = 6.7 Hz, H-9), 1.55 (1H, d, *J* = 6.7 Hz, H-10). ^{13}C NMR (100 MHz, CD_3OD) δ_C 135.9, 127.8, 127.2, 68.6, 22.4. HRESIMS *m/z* 191.0809 $[M + H]^+$ (calcd for $C_{10}H_{11}N_2O_2^+$ 191.0815).
Compound **8** (equisetin): Brown powders. $[\alpha]_D^{20}$ – 256.0 (*c* 0.1, $CHCl_3$). Molecular formula: $C_{22}H_{31}NO_4$. ^{1}H NMR (400 MHz, $CDCl_3$) δ_H 5.41 (1H, br s, H-5), 5.40 (1H, br s, H-4), 5.25 (1H, m, H-14), 5.17 (1H, m, H-13), 4.03 (1H, dd, *J* = 11.5; 3.9 Hz, H-6′a), 3.88 (1H, m, H-6′b), 3.64 (1H, br s, H-5′), 3.35 (1H, m, H-3), 3.05 (1H, s, H-7′), 1.96 (1H, m, H-10a), 1.84 (1H, m, H-6), 1.81 (1H, m, H-7a), 1.76 (1H, m, H-9), 1.66 (1H, m, H-11), 1.53 (1H, m, H-15), 1.48 (1H, m, H-8), 1.46 (1H, br s, H-12), 1.11 (1H, m, H-9b), 1.04 (1H, m, H-10b), 0.92 (1H, d, *J* = 6.5 Hz, H-16), 0.90 (1H, m, H-7b). ^{13}C NMR (100 MHz, $CDCl_3$) δ_C 190.4 (C-1), 177.1 (C-2′), 131.1 (C-5), 130.2 (C-13), 127.2 (C-4), 126.8 (C-14), 66.7 (C-5′), 60.6 (C-6′), 45.9 (C-2), 45.2 (C-3), 42.4 (C-7), 40.1 (C-6), 38.8 (C-11), 35.8 (C-9), 33.7 (C-8), 28.4 (C-10), 27.5 (C-7′), 22.6 (C-16), 18.1 (C-15), 14.2 (C-12). HRESIMS *m/z* 374.2308 $[M + H]^+$ (calcd for $C_{22}H_{32}NO_4^+$ 374.2326).

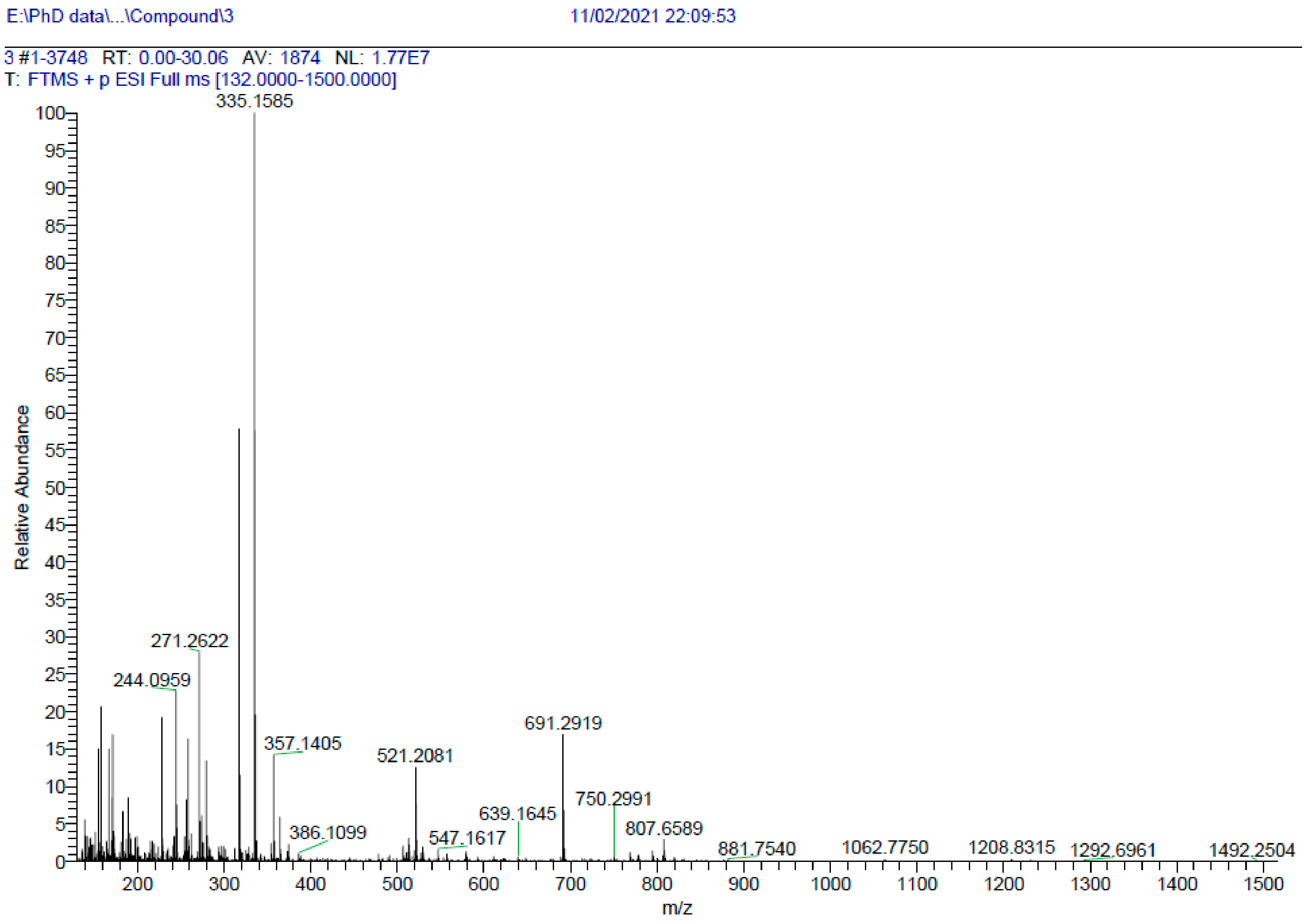

Figure S15. HRESI(+)MS of **3**

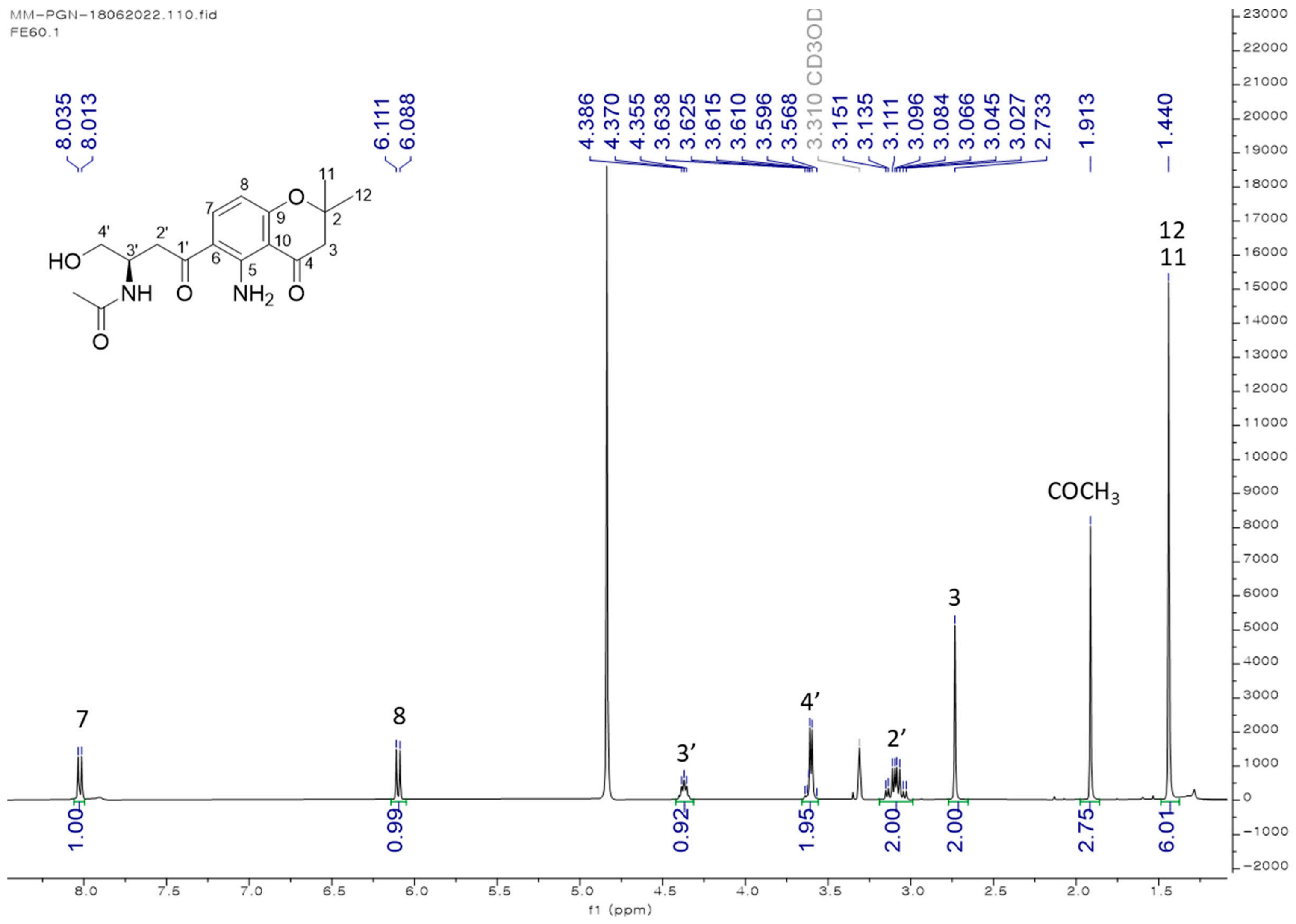

Figure S16. ^{1}H NMR (400 MHz) spectrum of **3** in CD_3OD

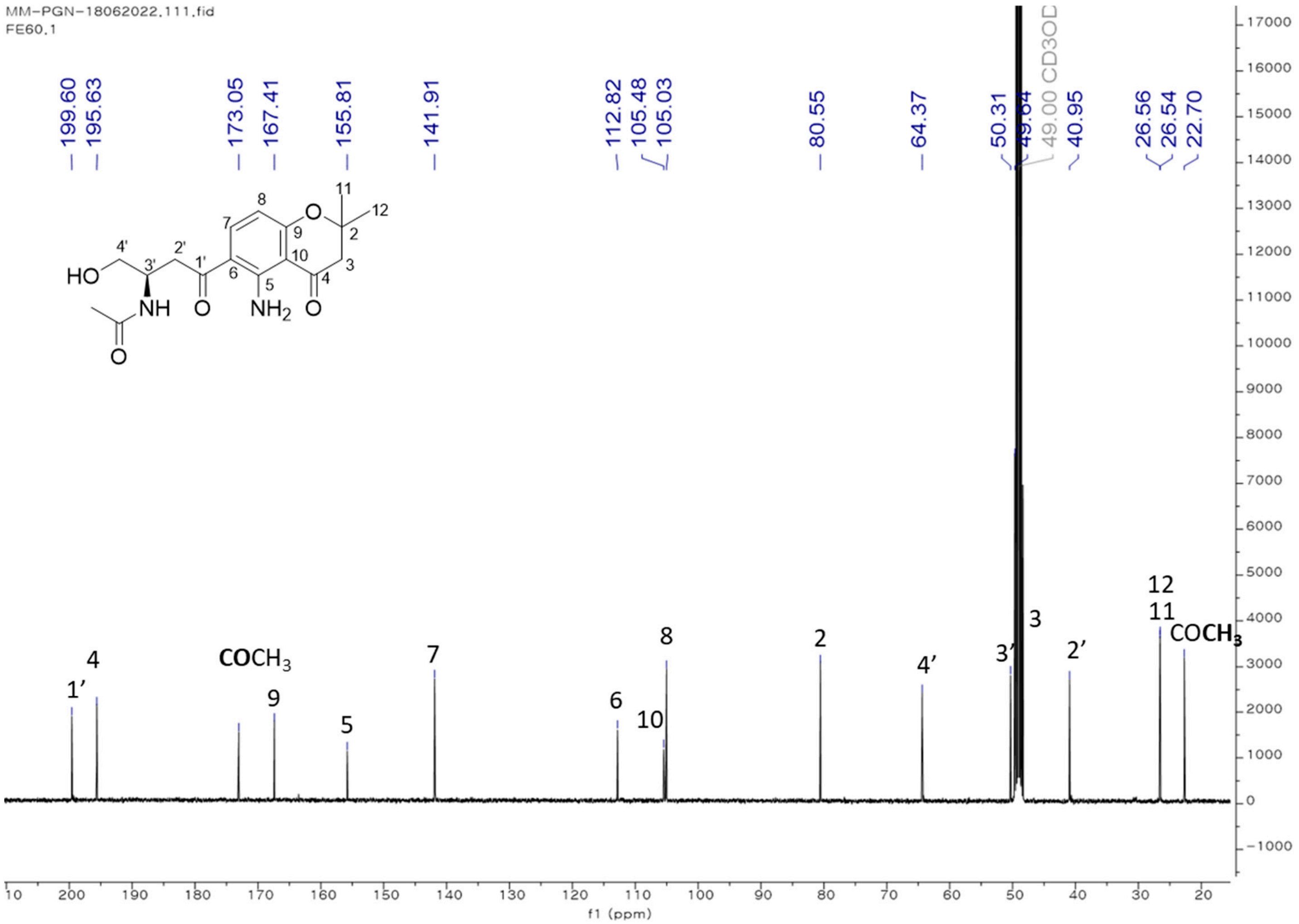

Figure S17. ^{13}C NMR (100 MHz) spectrum of **3** in CD_3OD

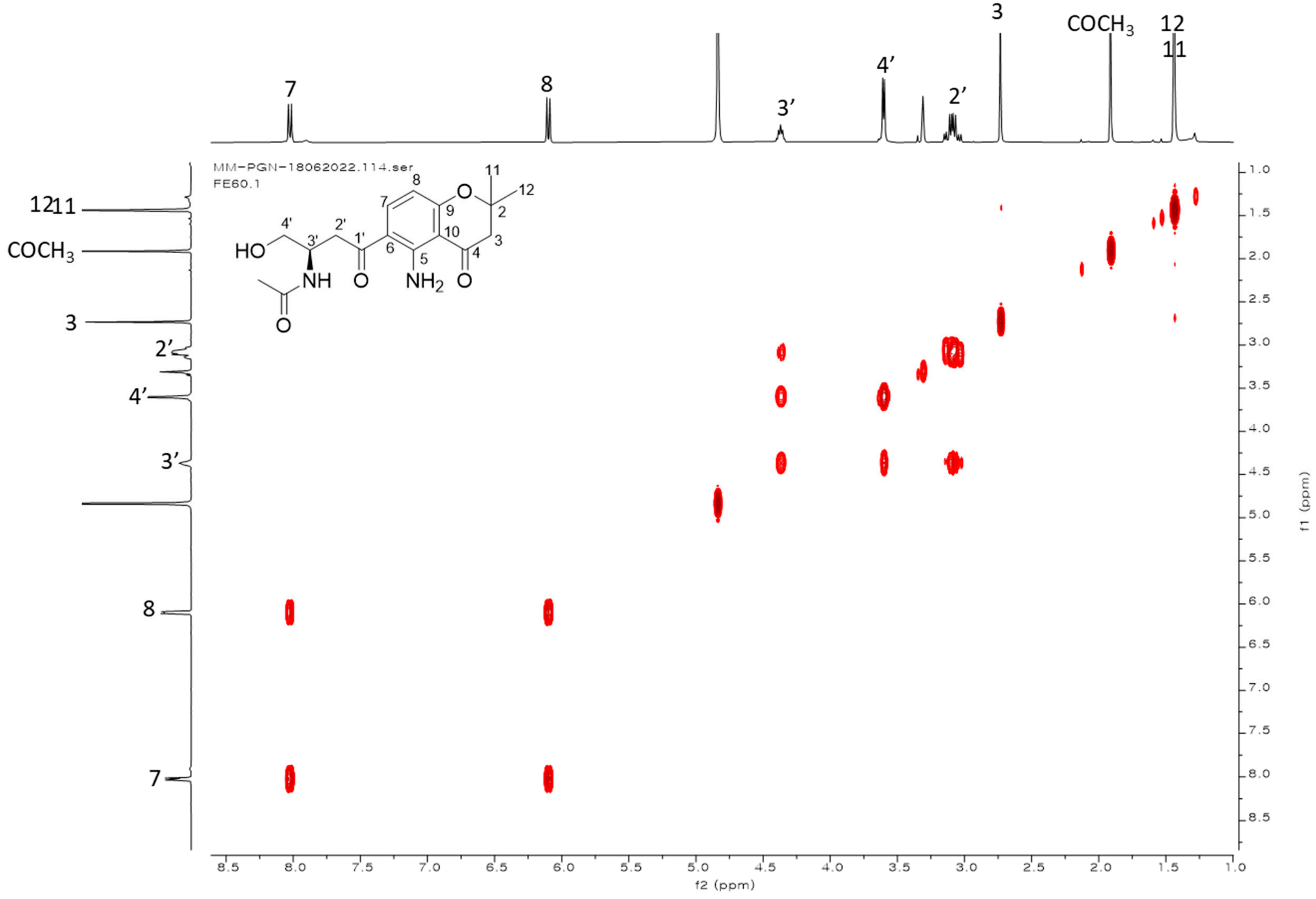

Figure S18. ^{1}H-^{1}H COSY spectrum of **3** in CD_3OD

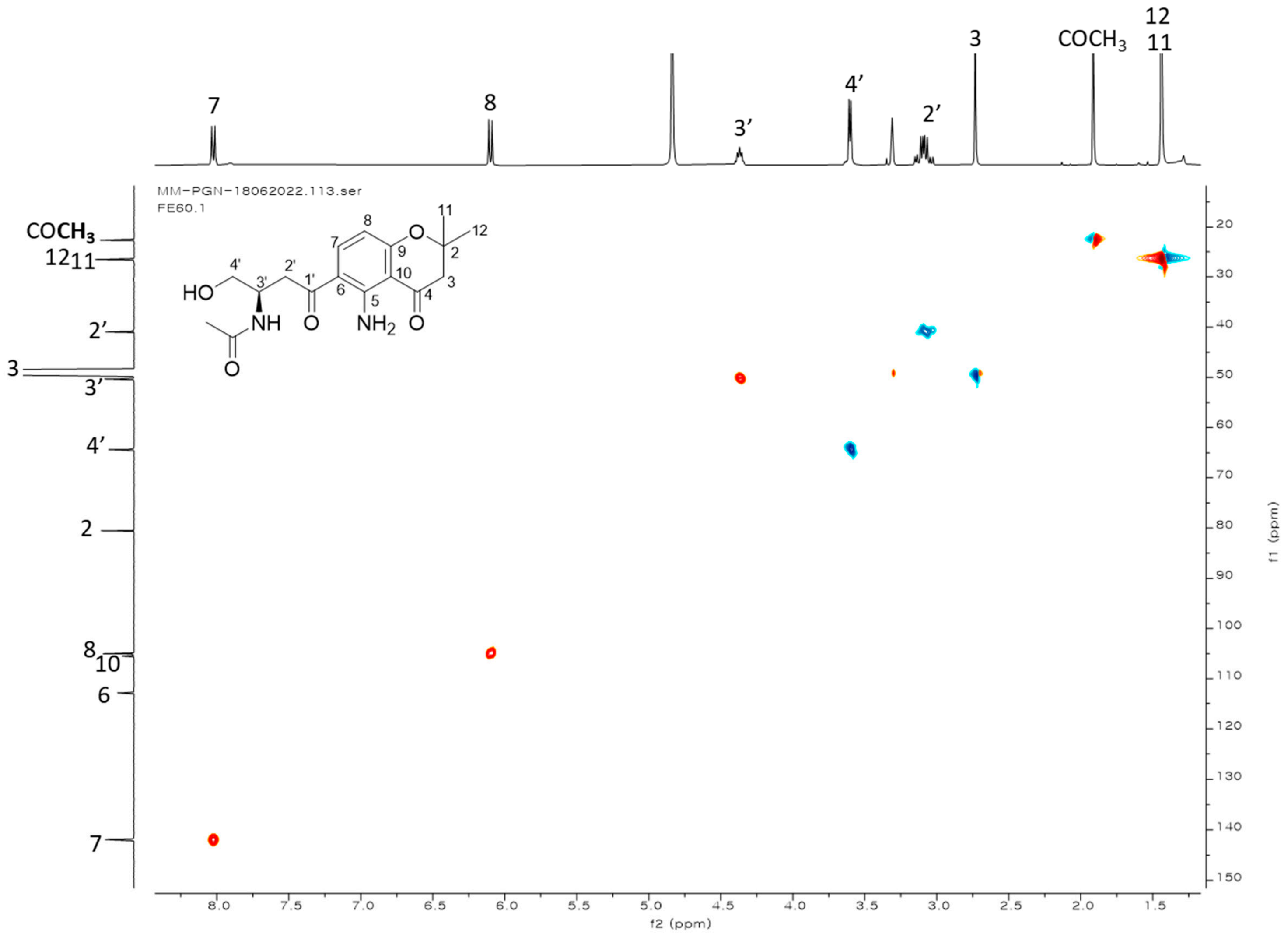

Figure S19. ^{1}H-^{13}C HSQC spectrum of **3** in CD_3OD

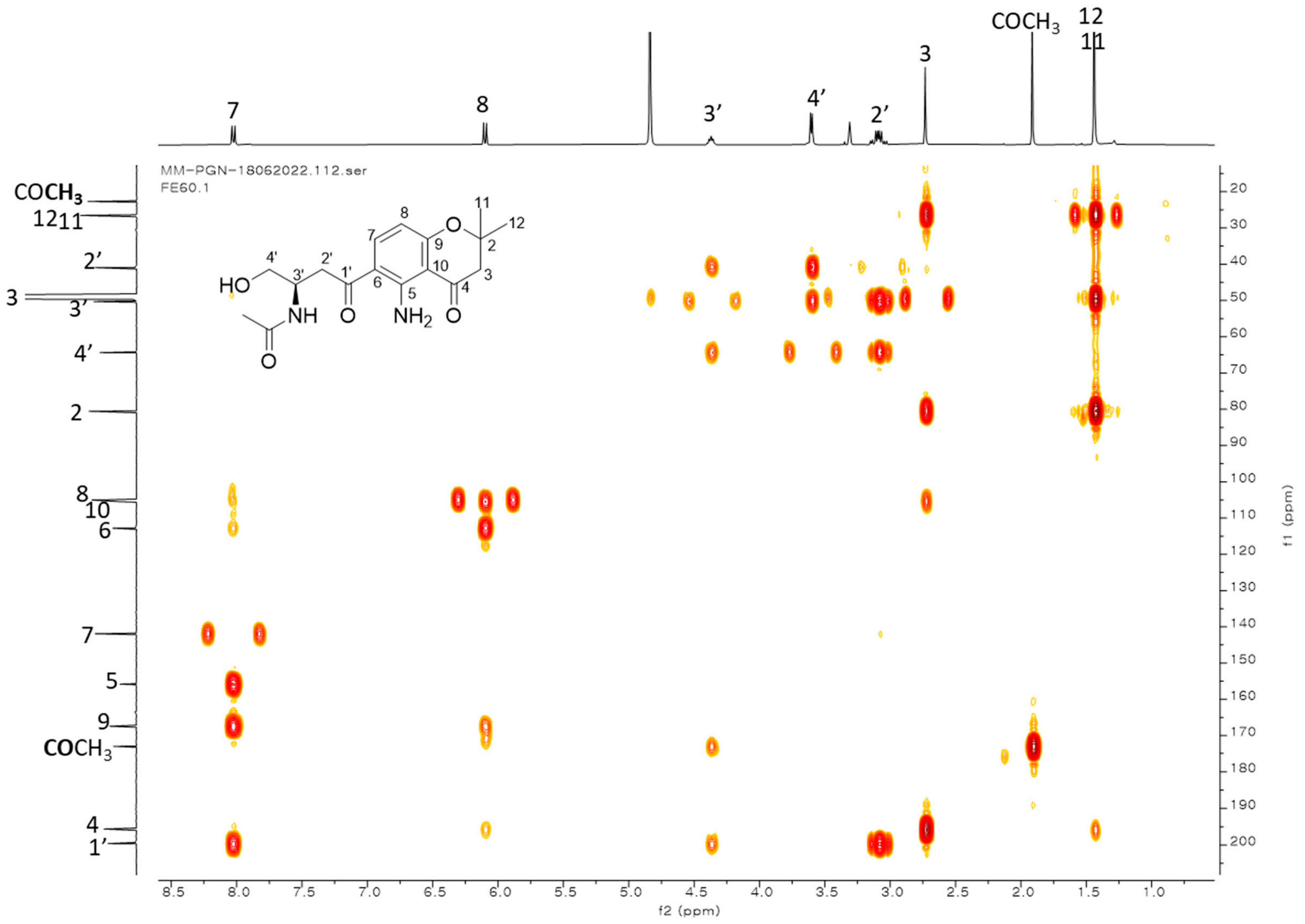

Figure S20. ^{1}H-^{13}C HMBC spectrum of **3** in CD_3OD

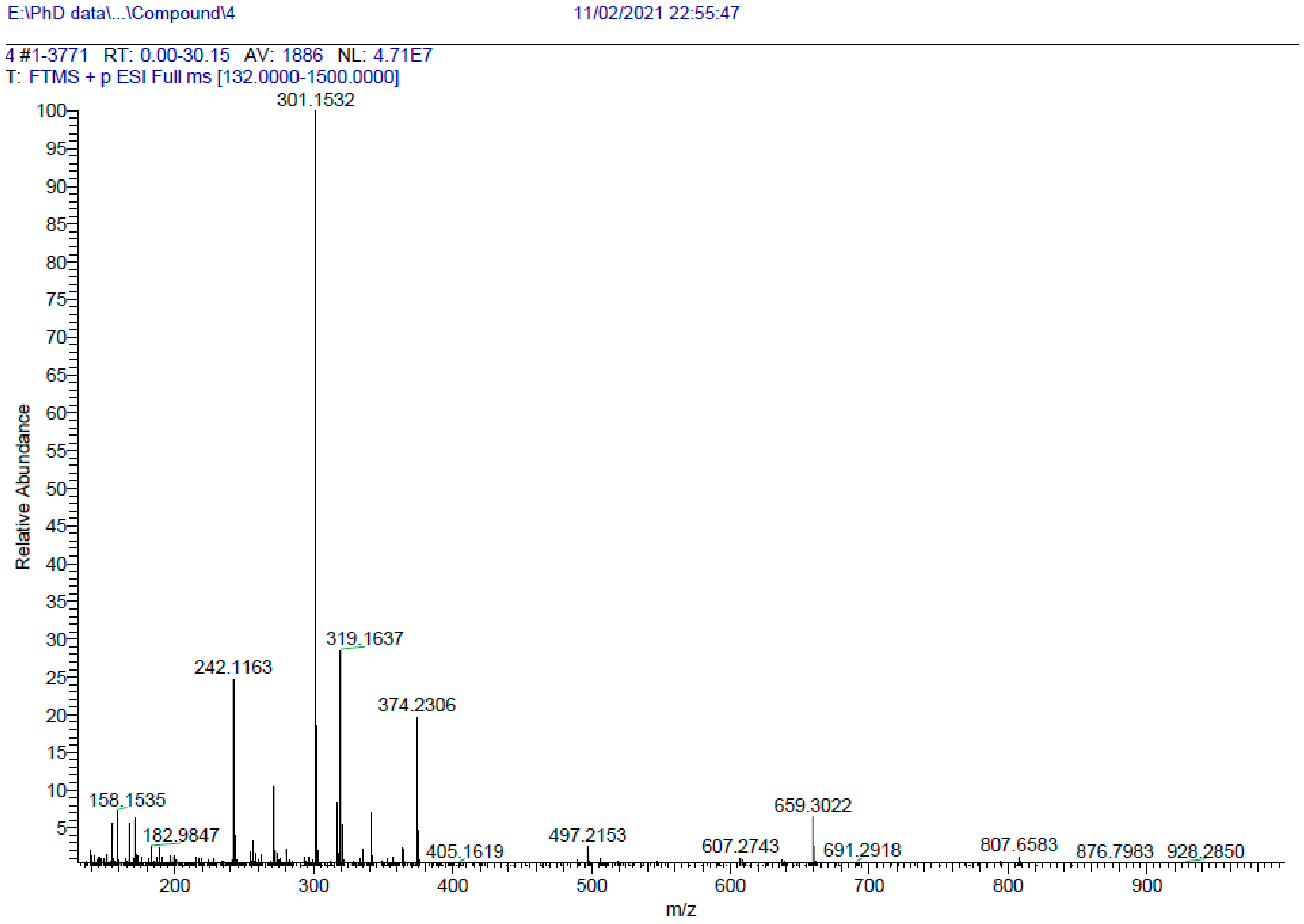

Figure S21. HRESI(+)MS of **4**

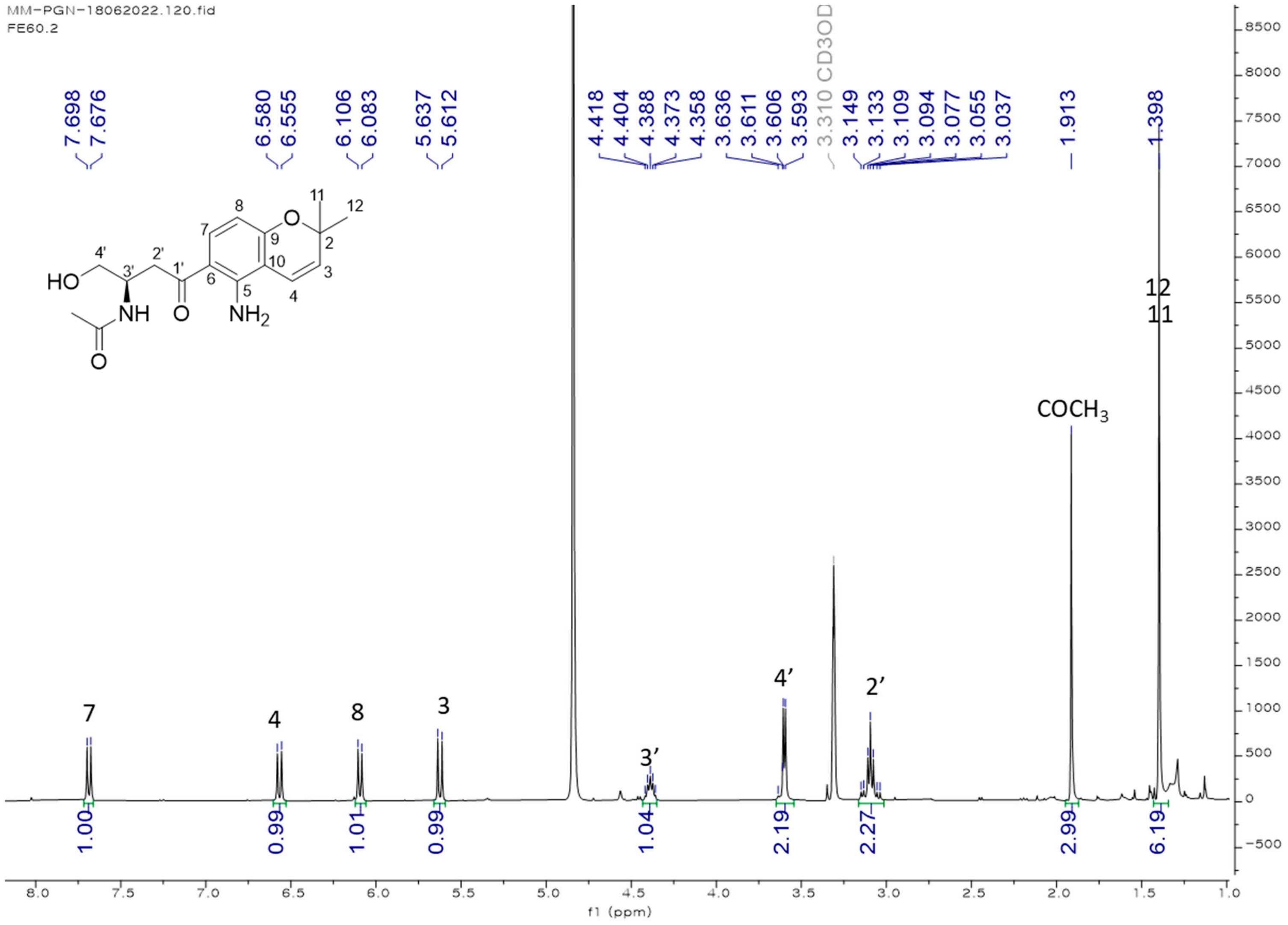

Figure S22. ^{1}H NMR (400 MHz) spectrum of **4** in CD_3OD

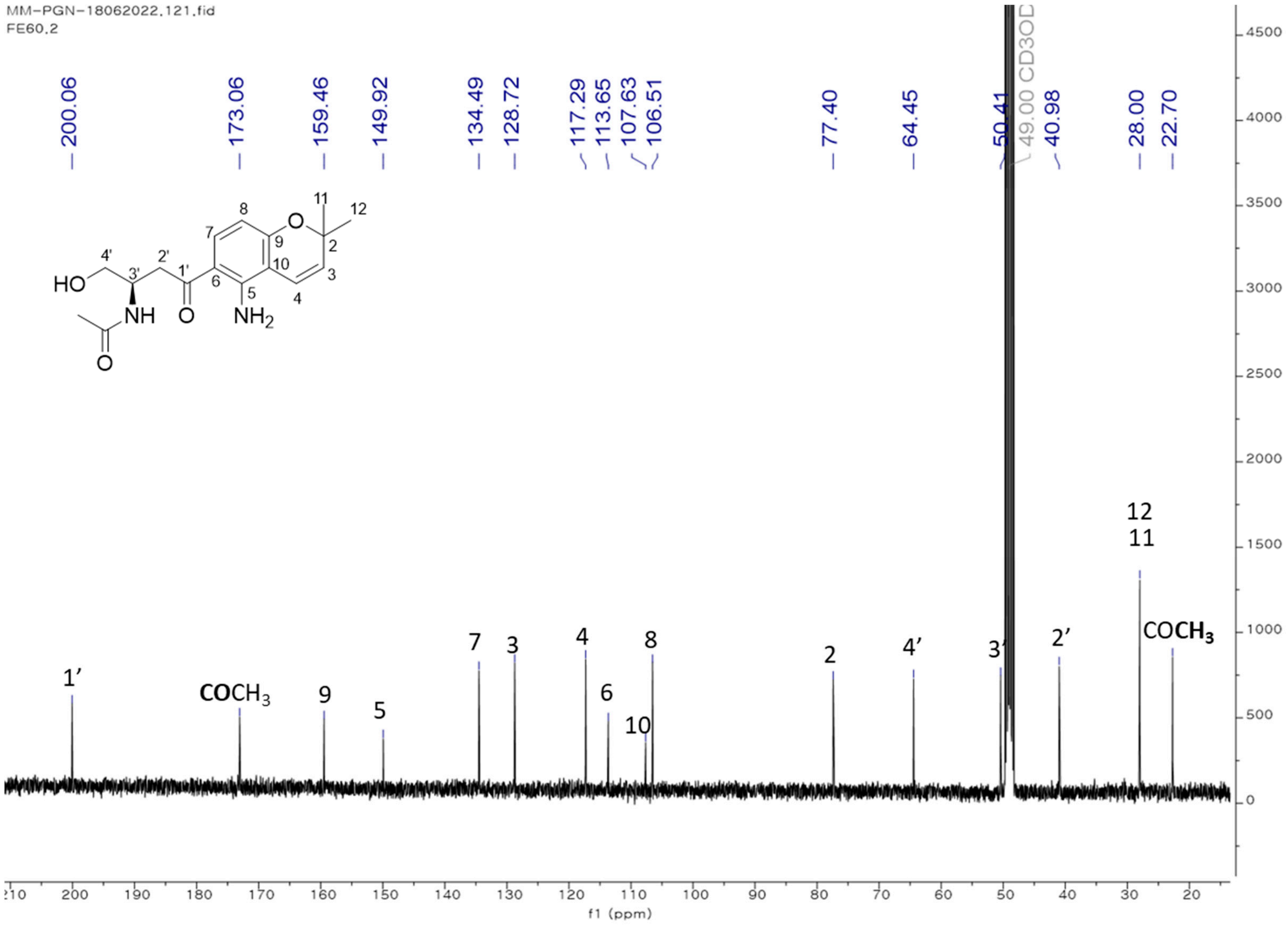

Figure S23. ^{13}C NMR (100 MHz) spectrum of **4** in CD_3OD

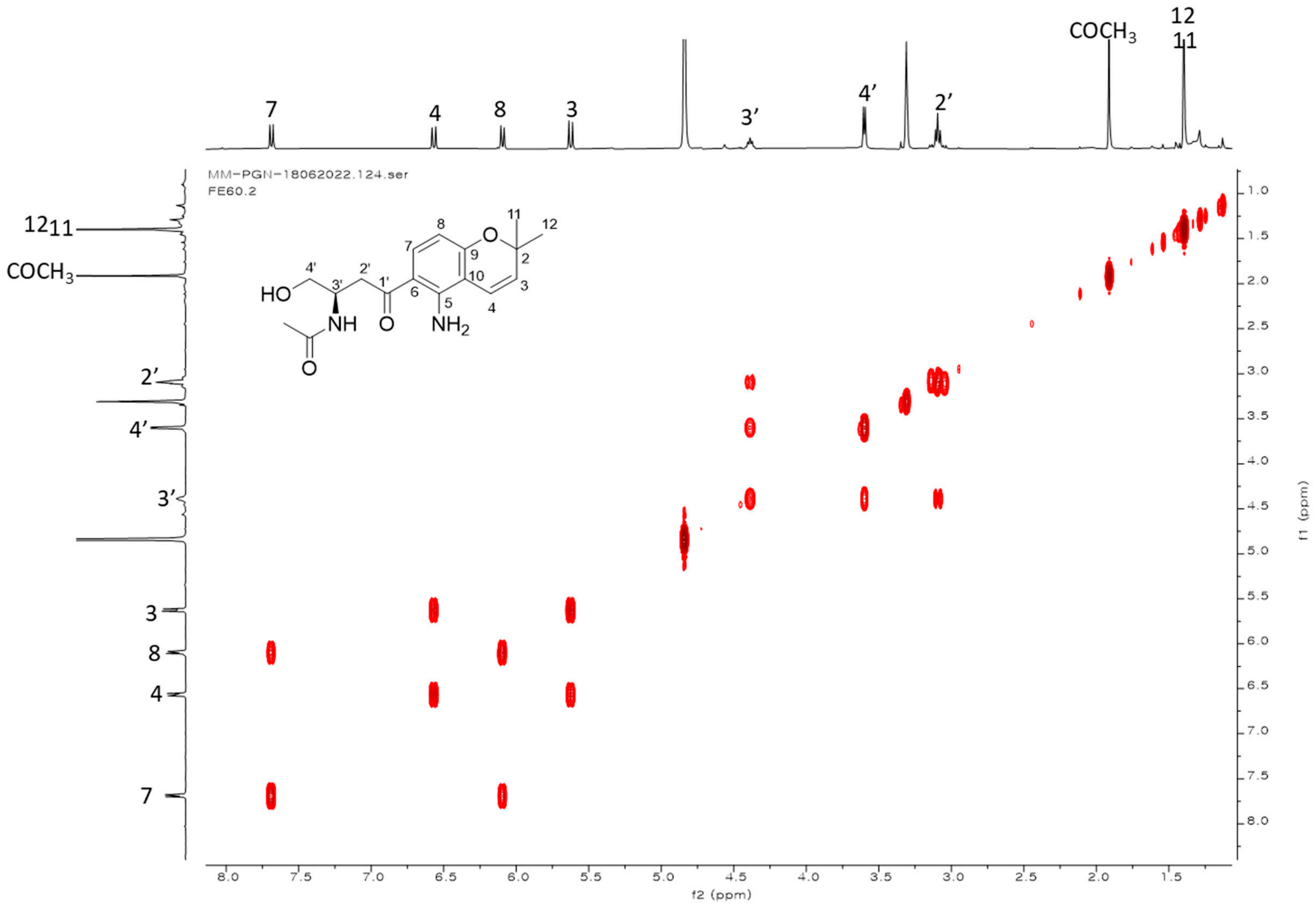

Figure S24. ^{1}H-^{1}H COSY spectrum of **4** in CD3OD

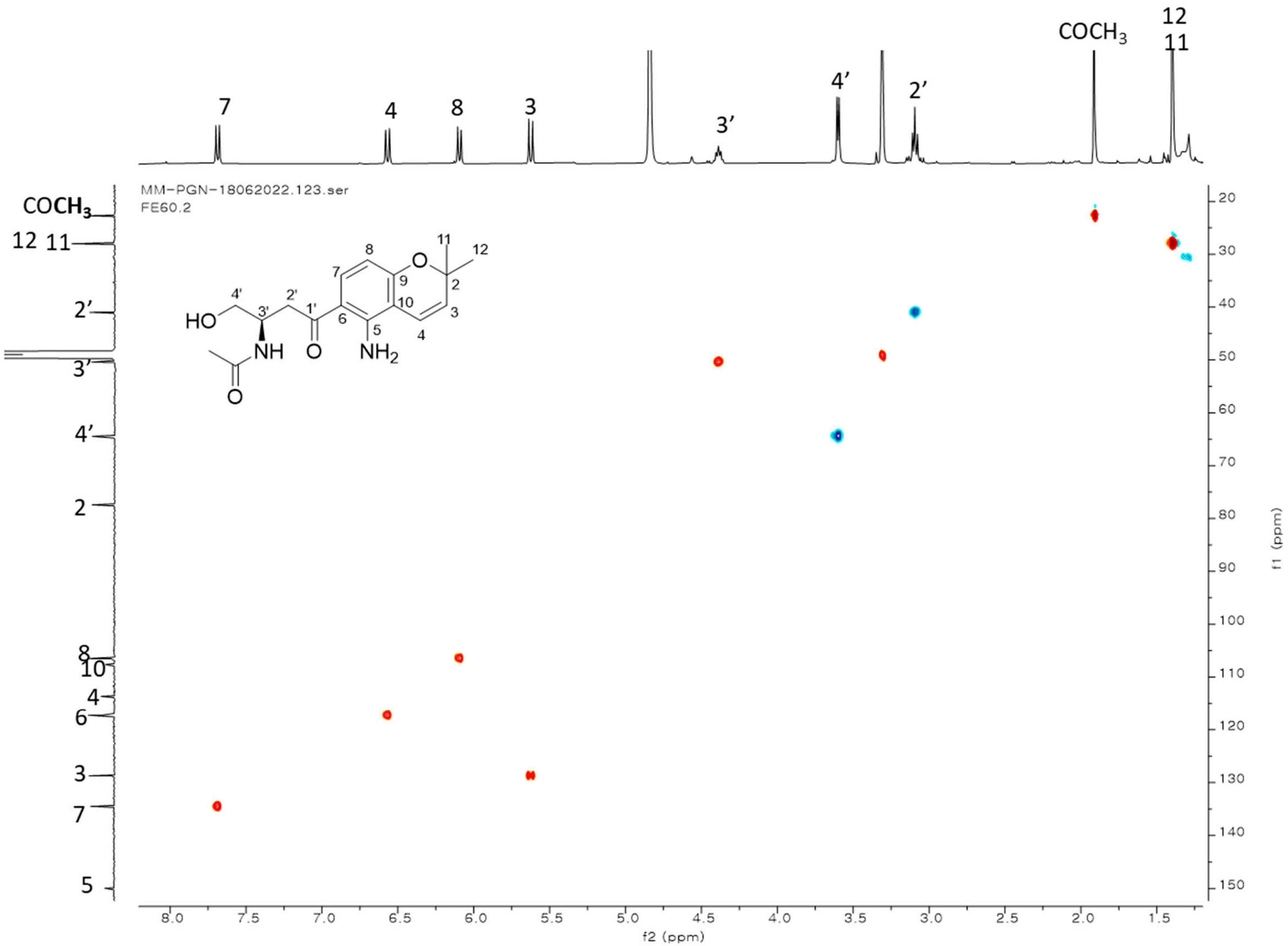

Figure S25. ^1H-^{13}C HSQC spectrum of **4** in CD_3OD

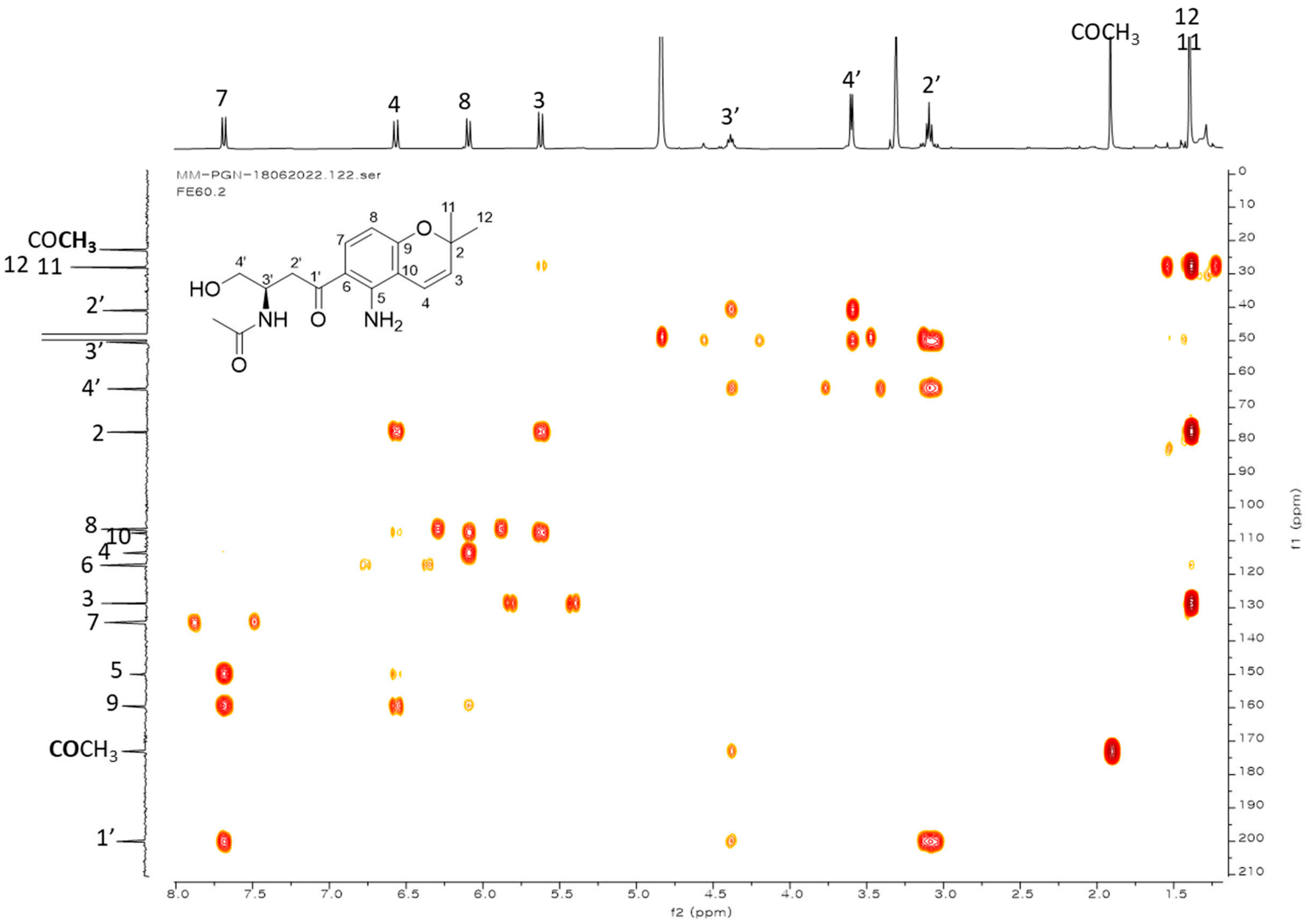

Figure S26. ^{1}H-^{13}C HMBC spectrum of **4** in CD_3OD

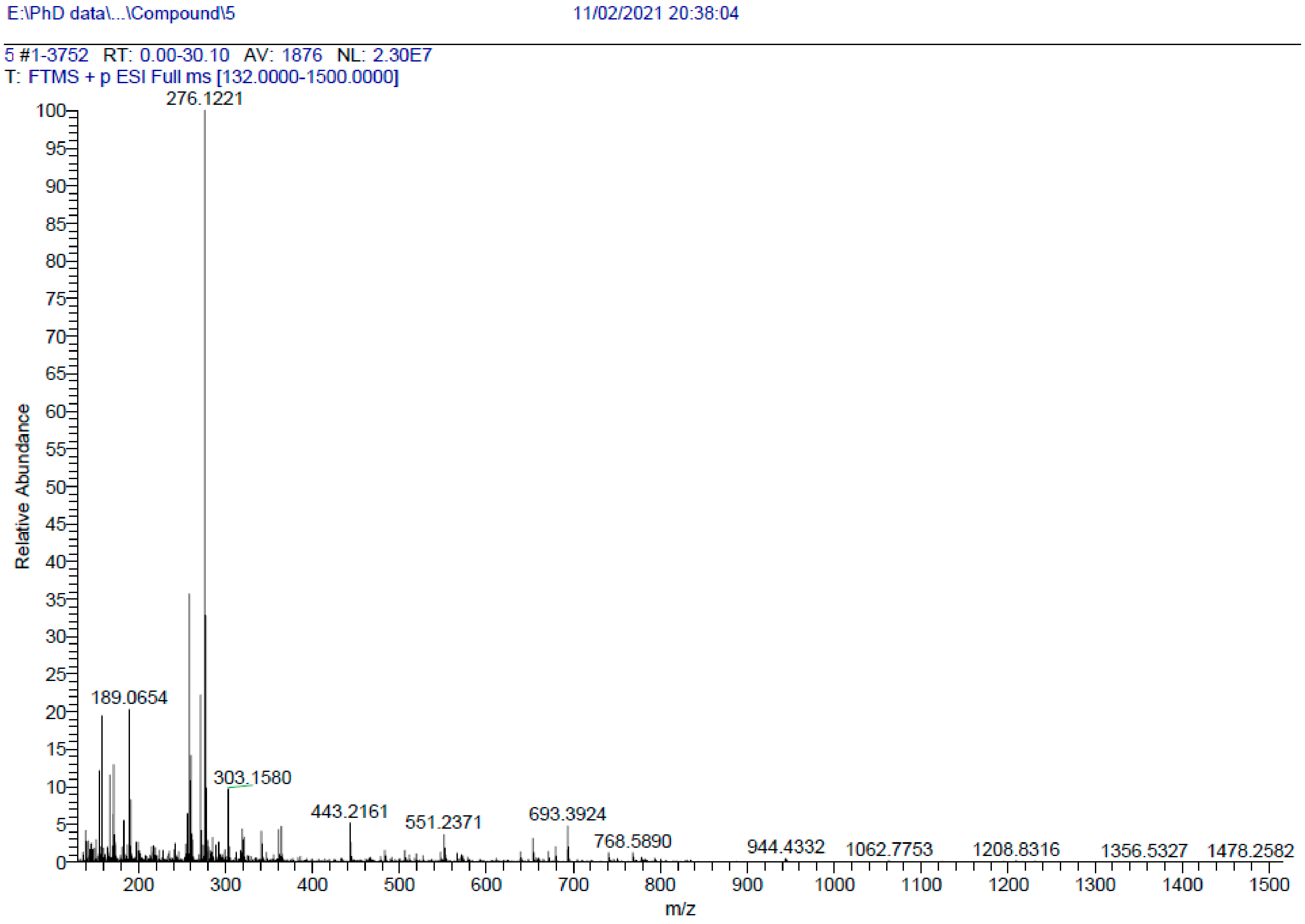

Figure S27. HRESI(+)MS of **5**

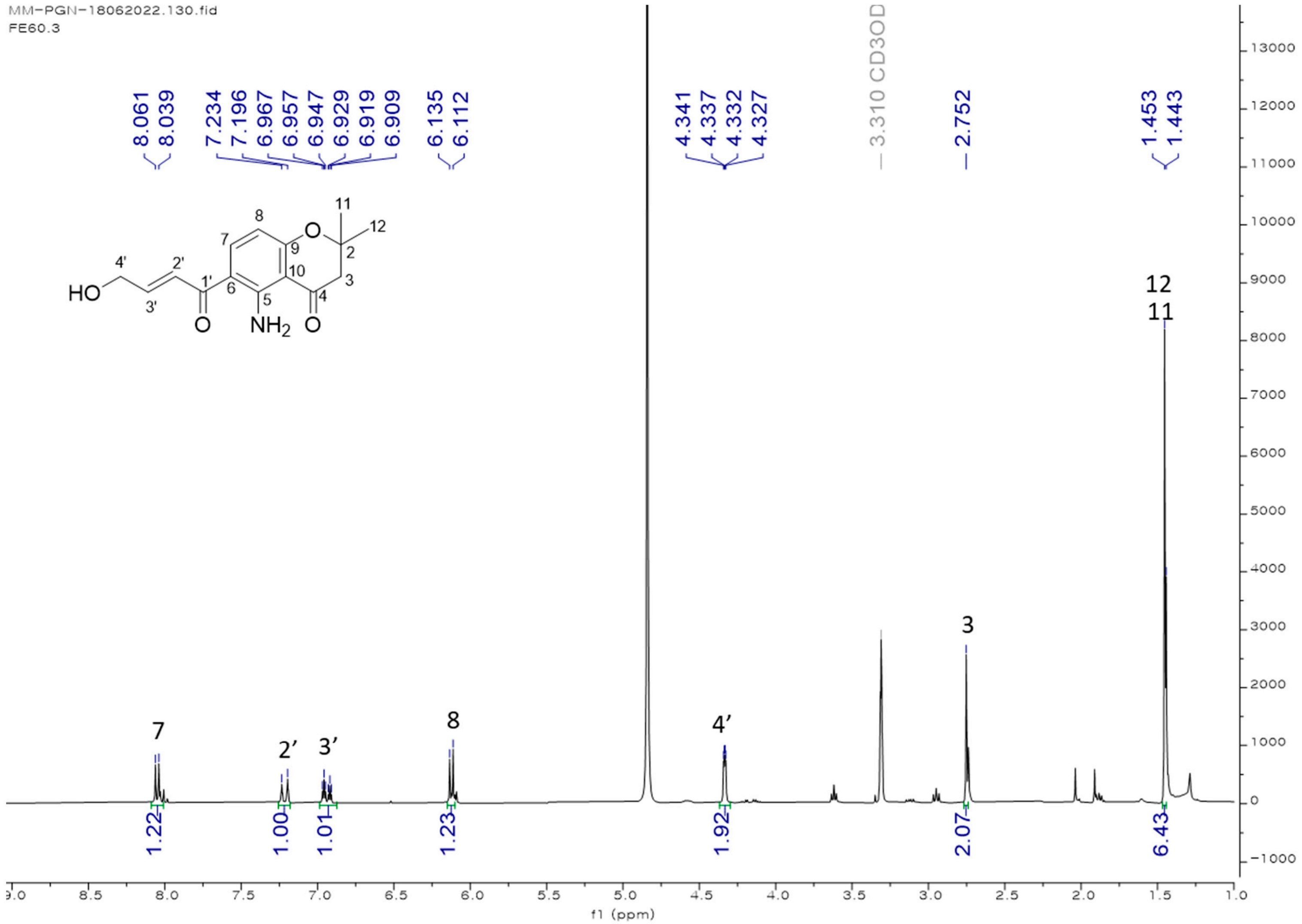

Figure S28. ^{1}H NMR (400 MHz) spectrum of **5** in CD_3OD

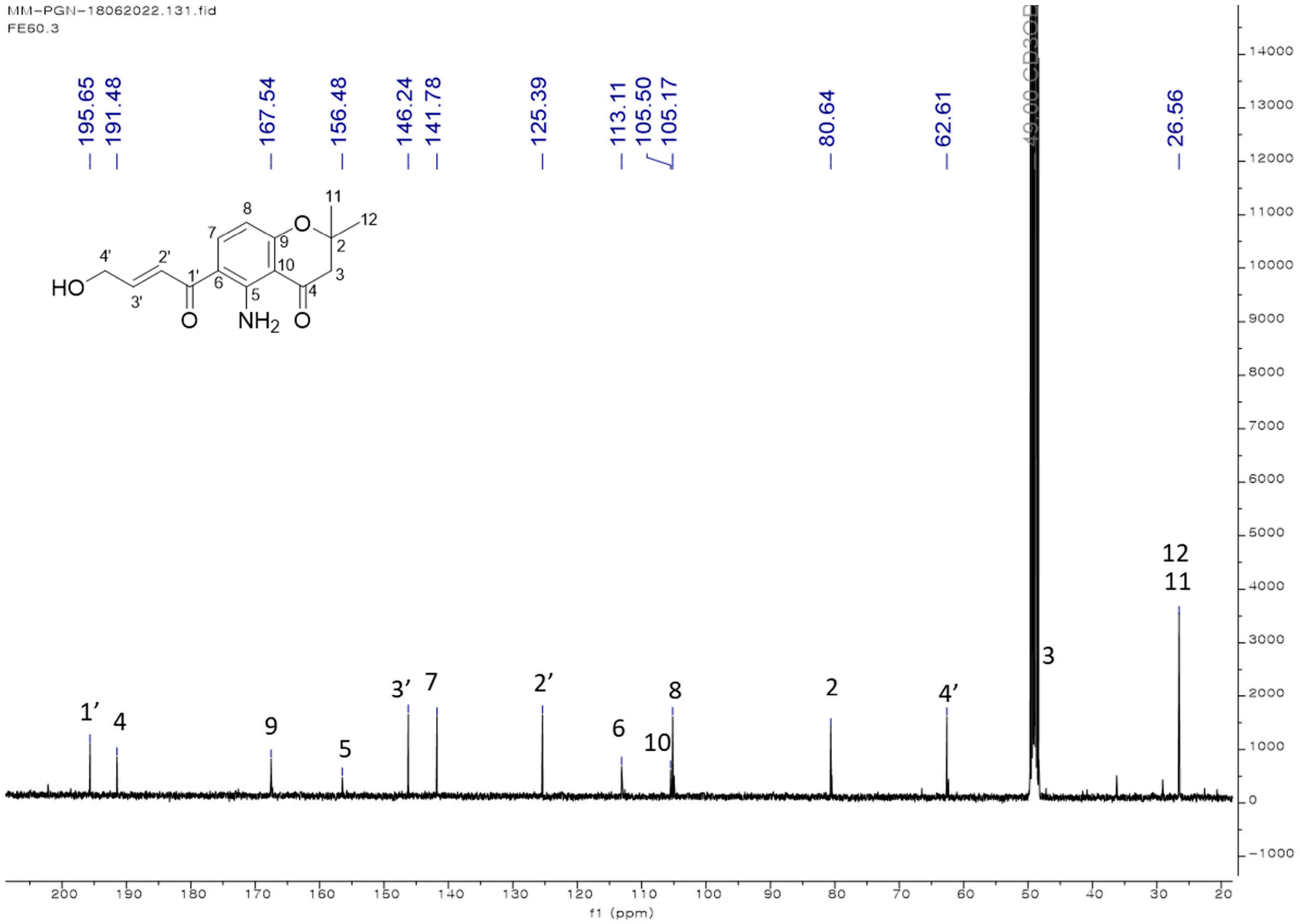

Figure S29. ^{13}C NMR (100 MHz) spectrum of **5** in CD_3OD

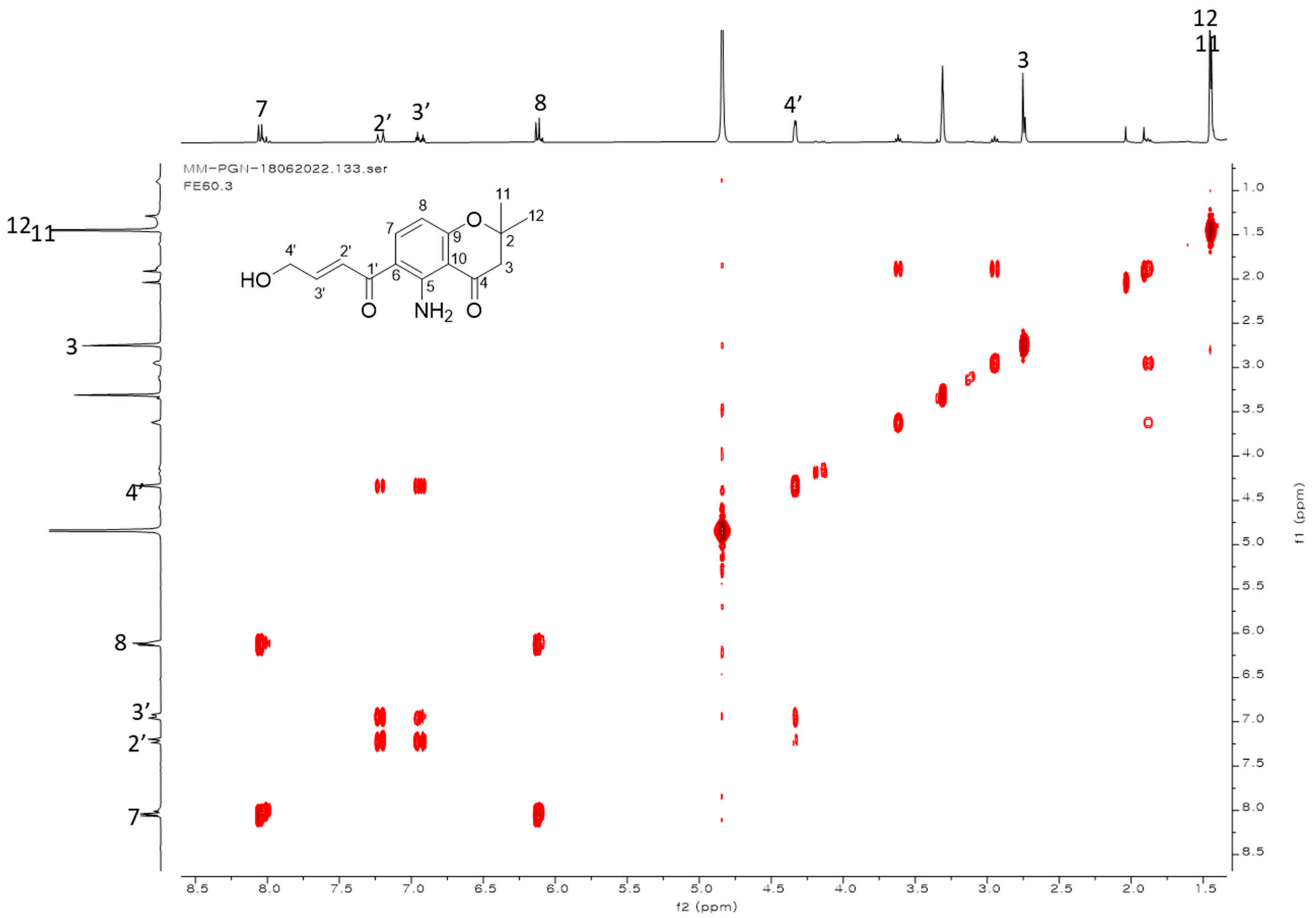

Figure S30. ^{1}H-^{1}H COSY spectrum of **5** in CD_3OD

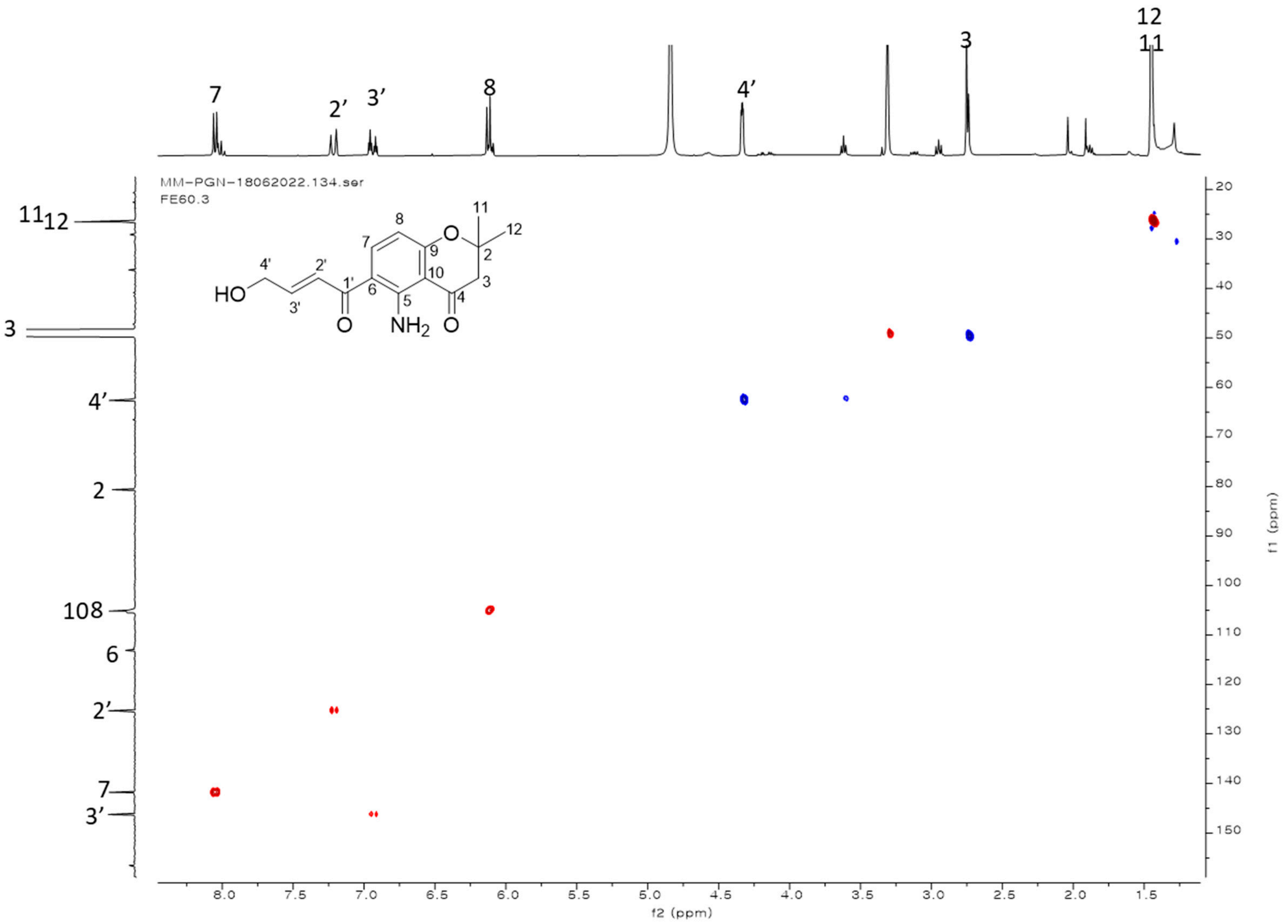

Figure S31. ^1H-^{13}C HSQC spectrum of **5** in CD_3OD

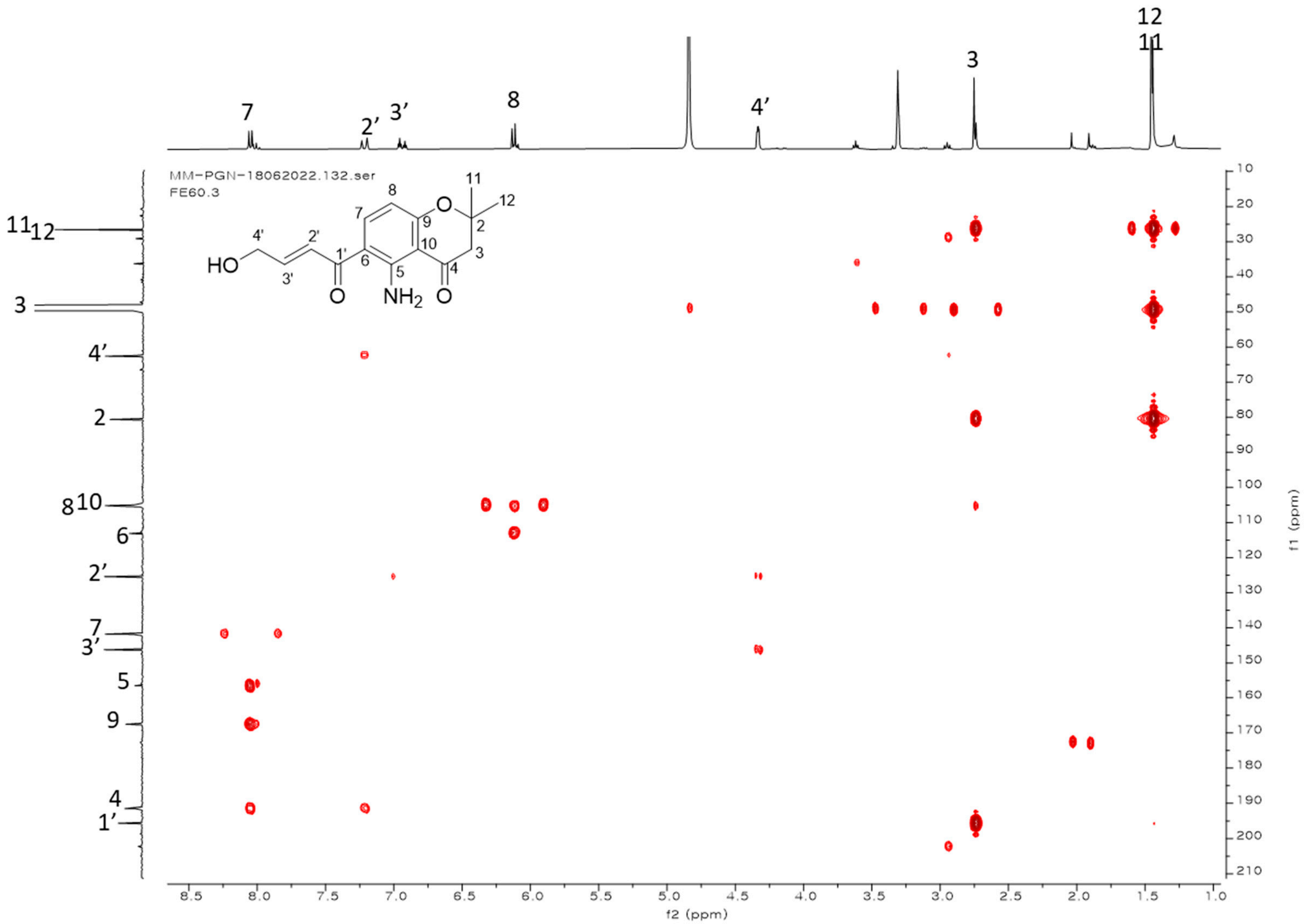

Figure S32. ^{1}H-^{13}C HMBC spectrum of **5** in CD_3OD

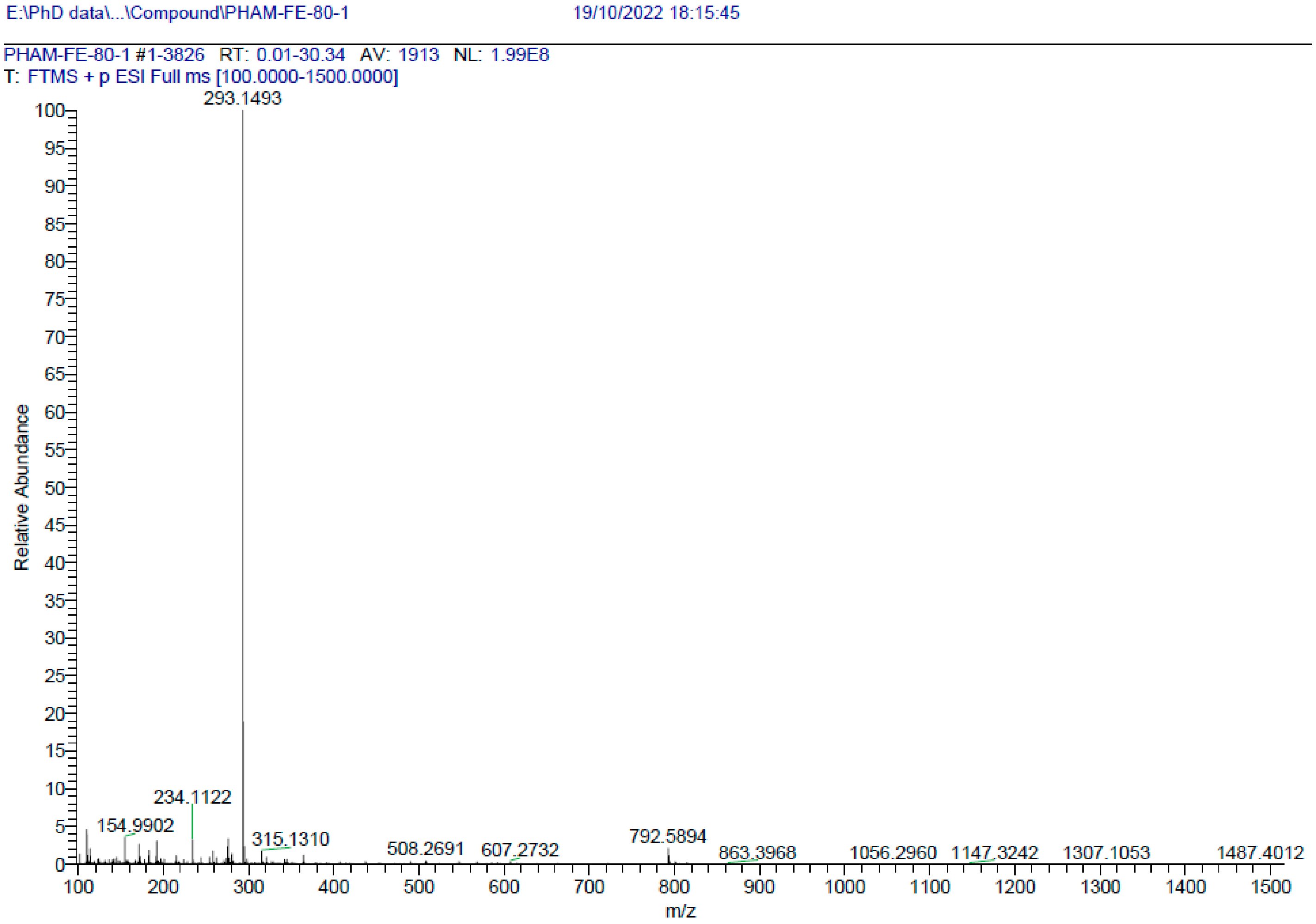

Figure S33. HRESI(+)MS of **6**

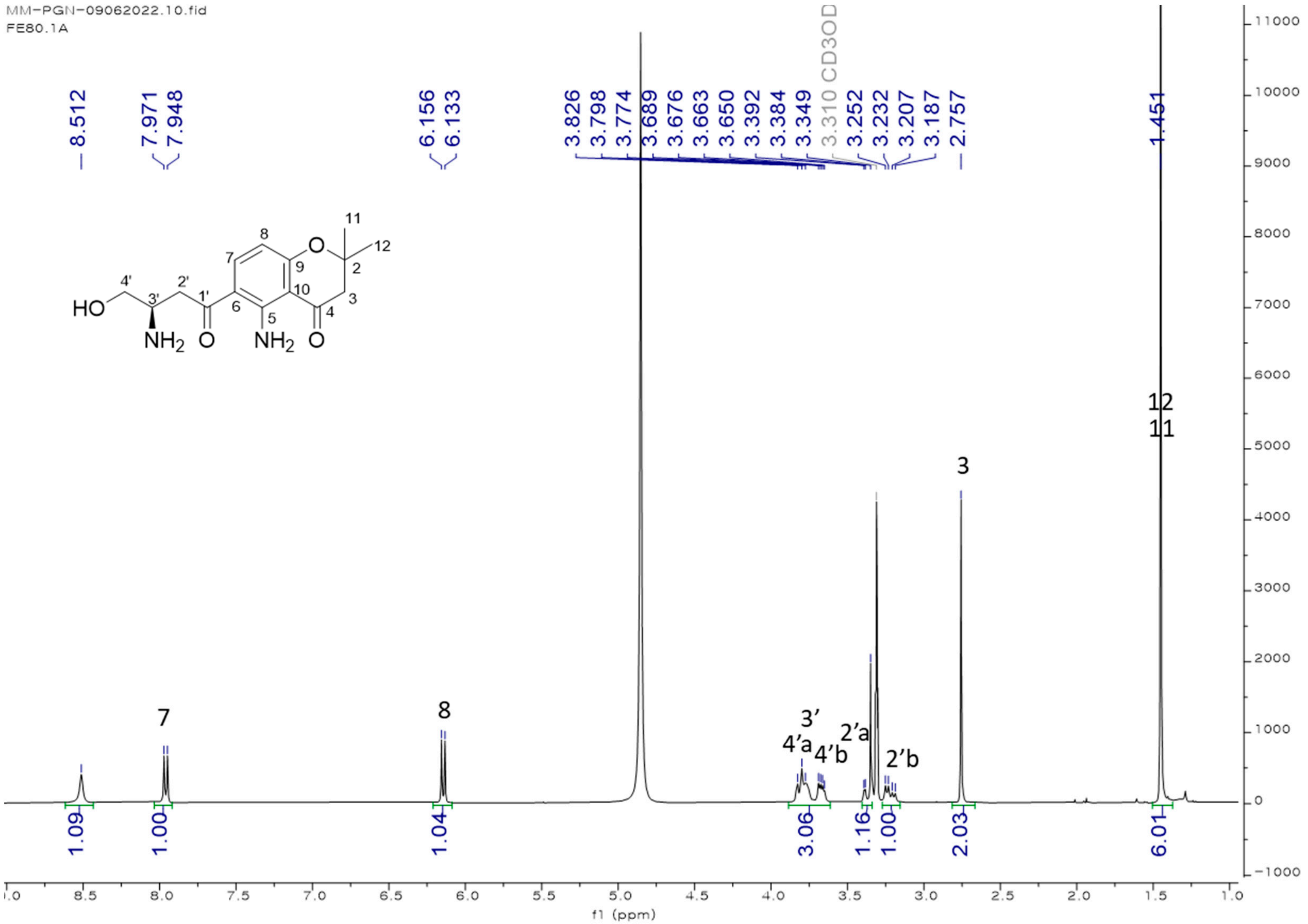

Figure S34. ^{1}H NMR (400 MHz) spectrum of **6** in CD_3OD

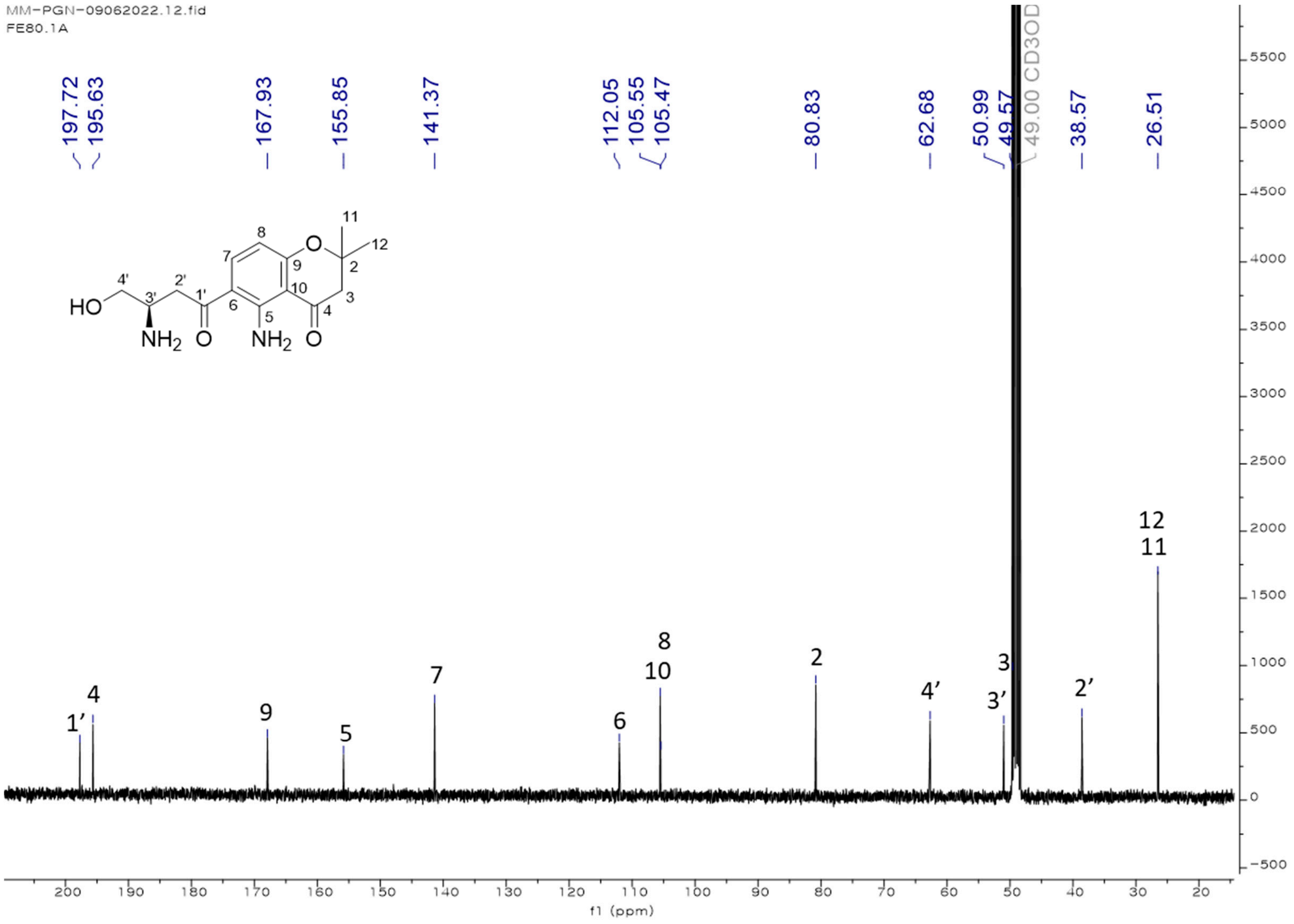

Figure S35. ^{13}C NMR (100 MHz) spectrum of **6** in CD_3OD

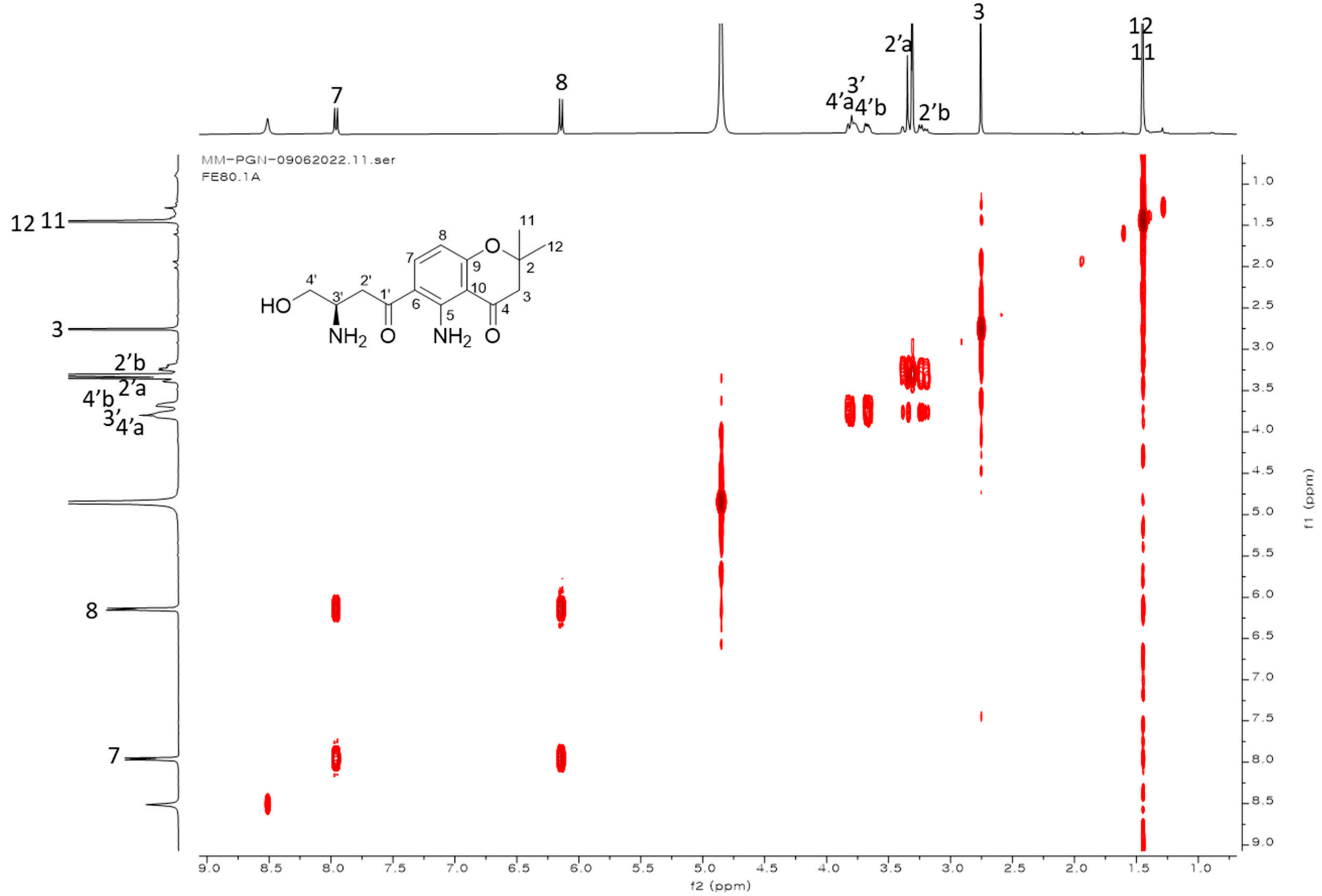

Figure S36. ^1H-^1H COSY spectrum of **6** in CD_3OD

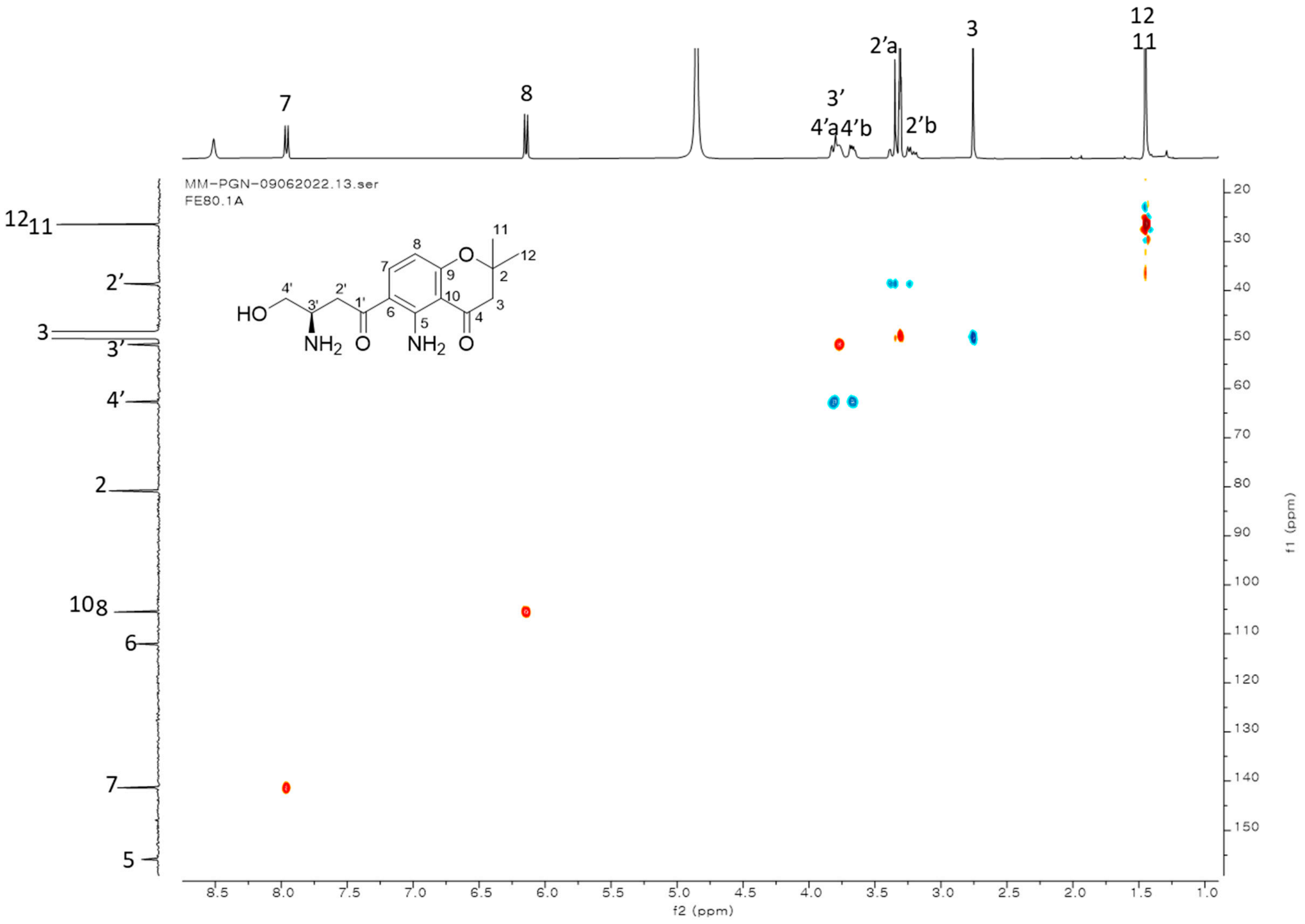

Figure S37. ^{1}H-^{13}C HSQC spectrum of **6** in CD_3OD

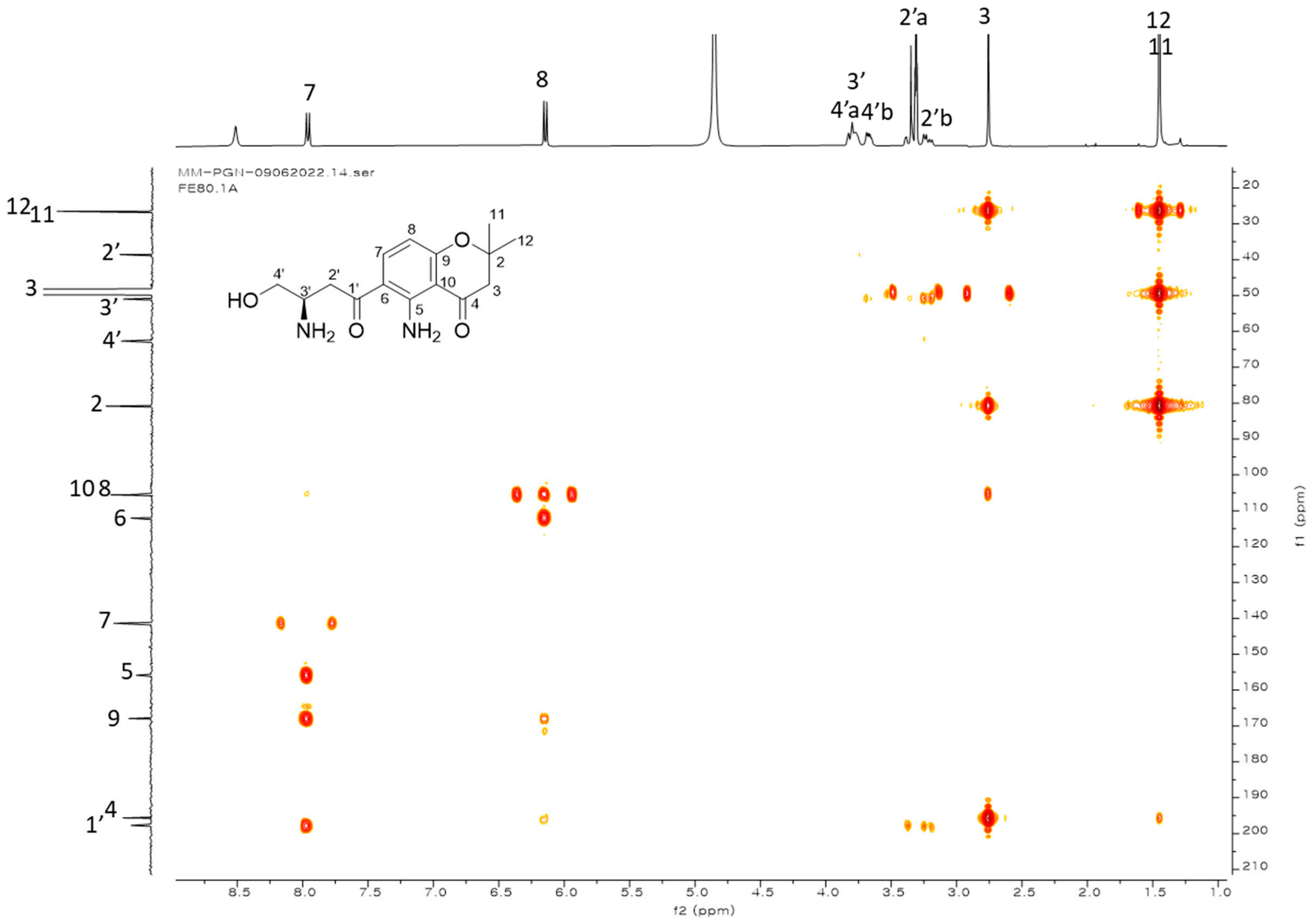

Figure S38. ^1H-^{13}C HMBC spectrum of **6** in CD_3OD

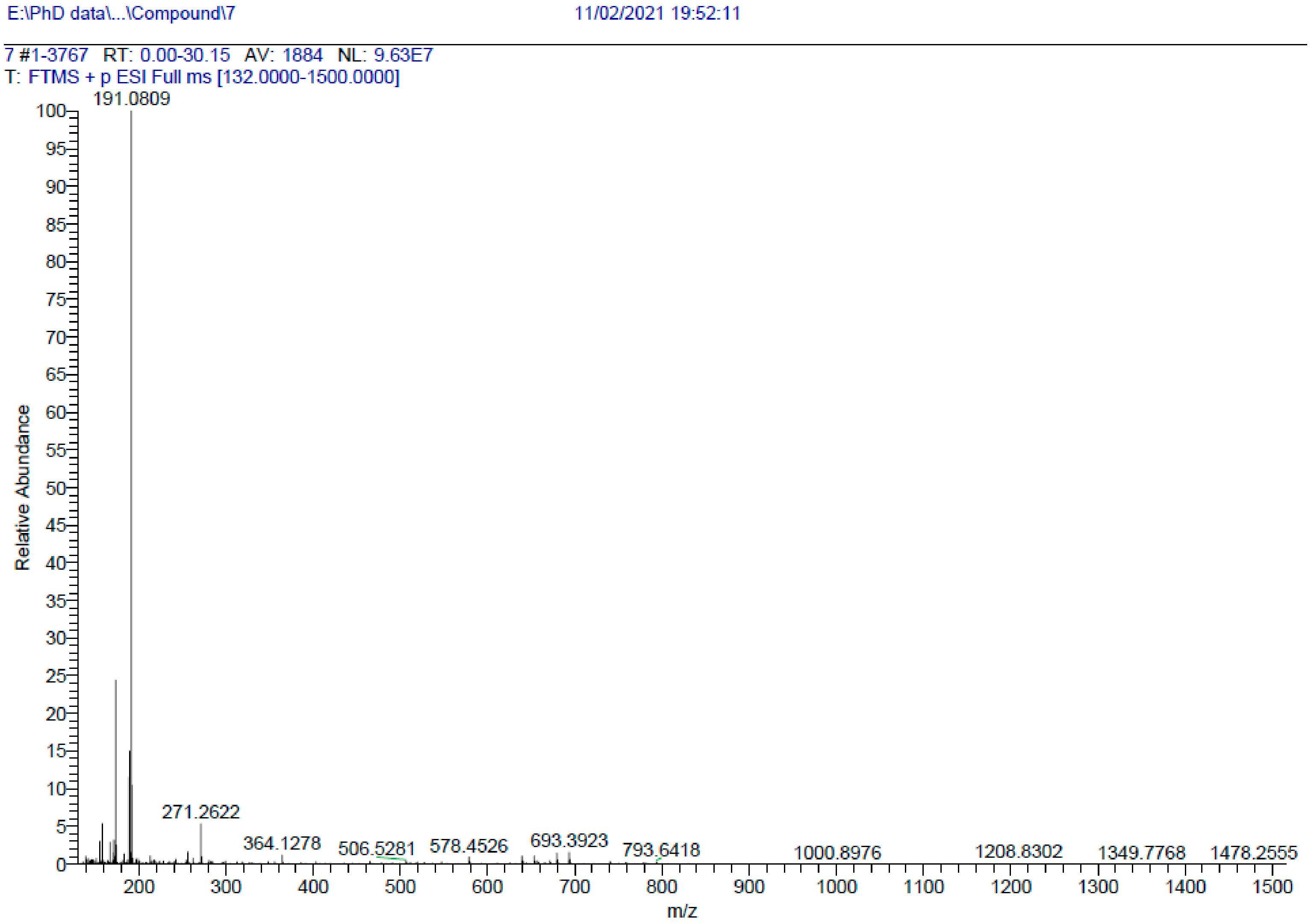

Figure S39. HRESI(+)MS of **7**

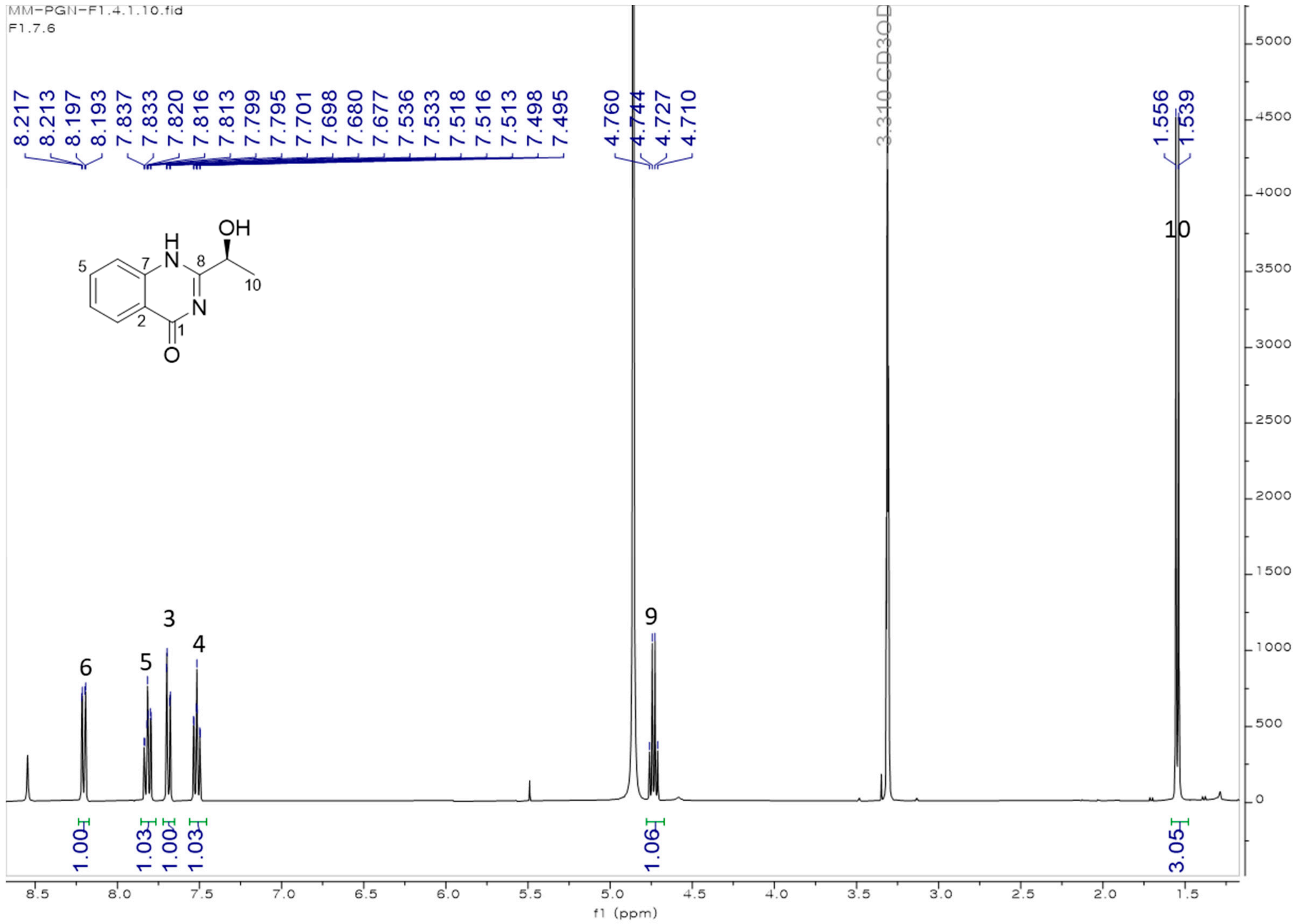

Figure S40. ^{1}H NMR (400 MHz) spectrum of **7** in CD_3OD

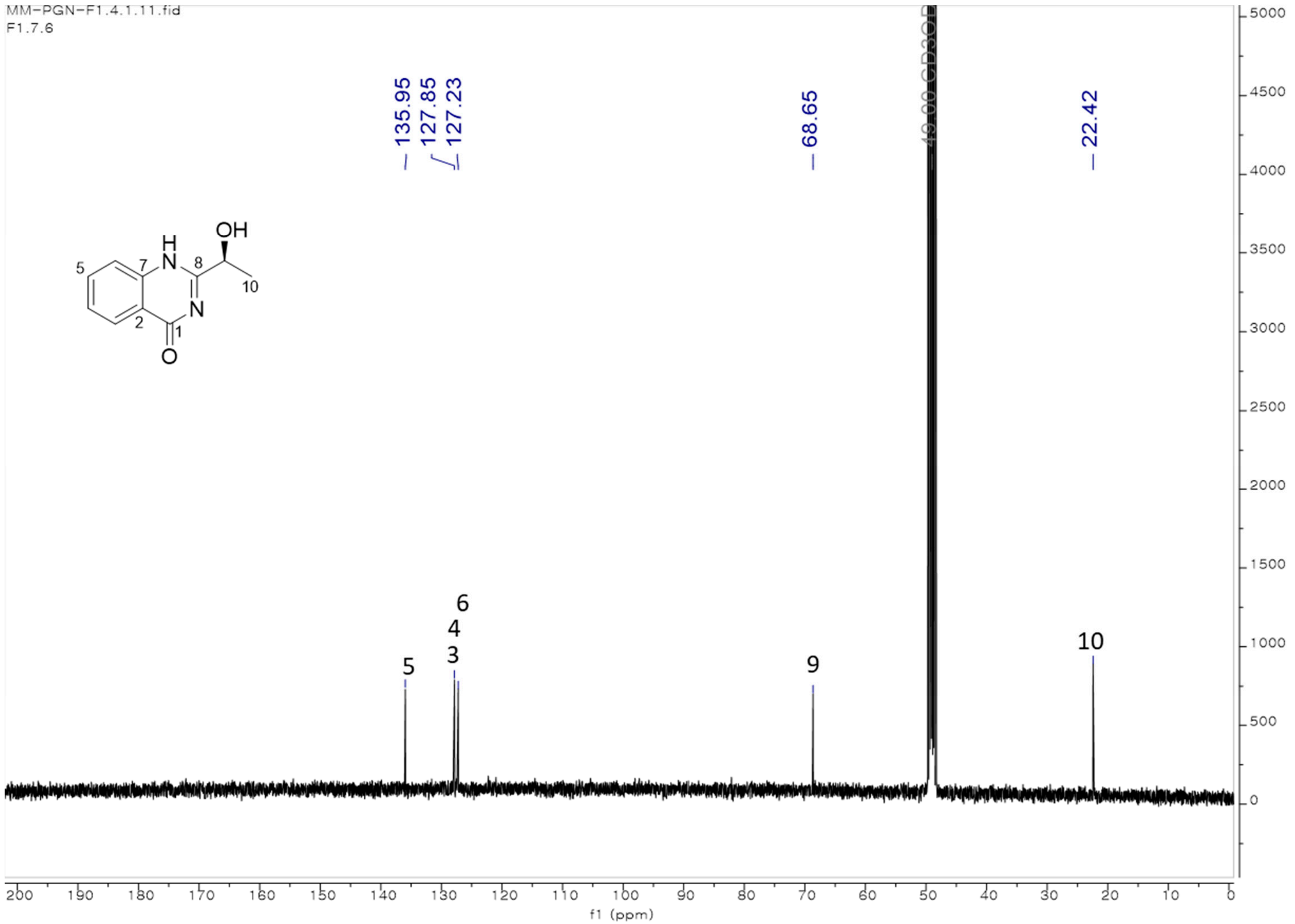

Figure S41. ^{13}C NMR (100 MHz) spectrum of **7** in CD_3OD

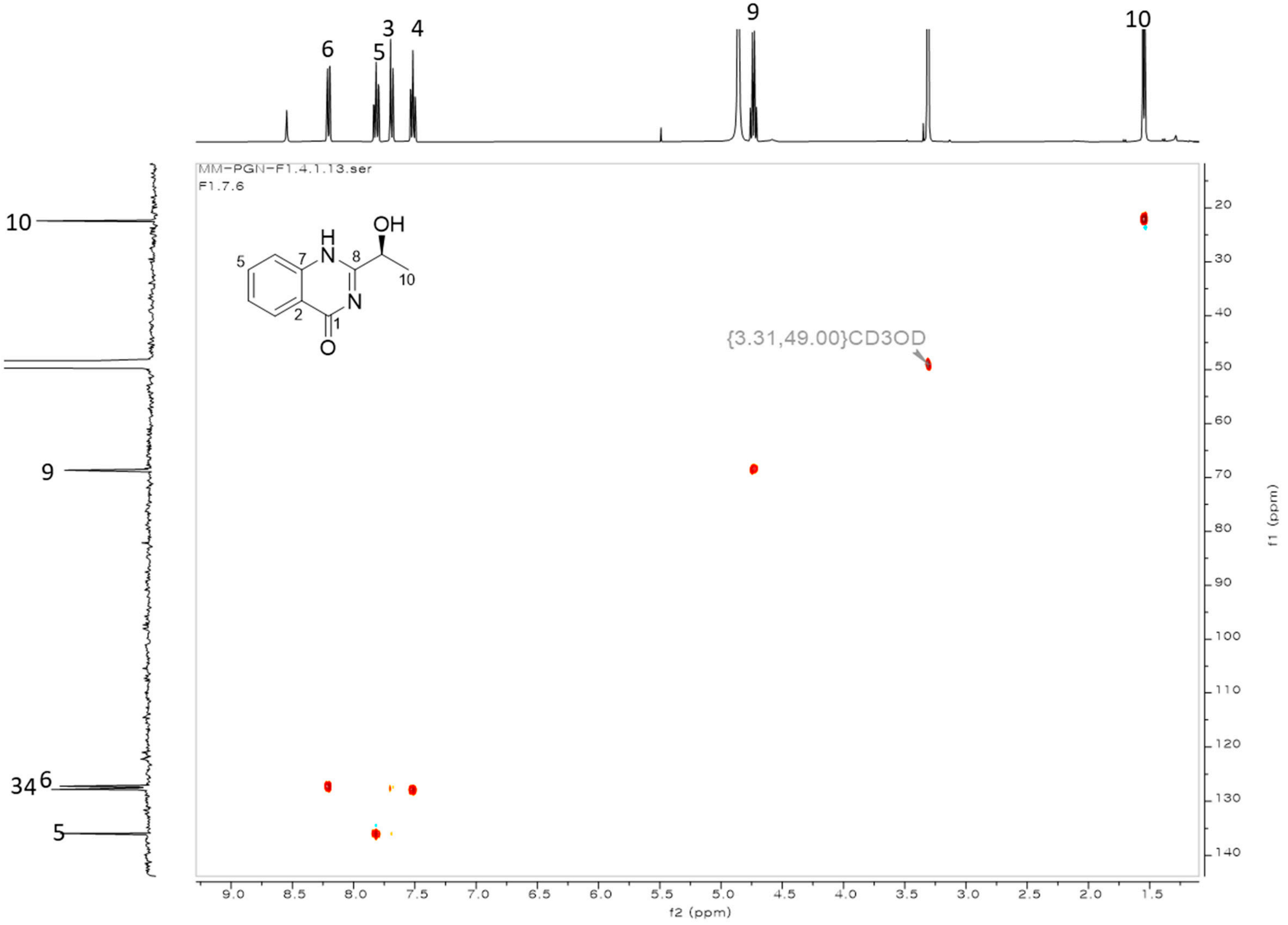

Figure S42. ^{1}H-^{13}C HSQC spectrum of **7** in CD_3OD

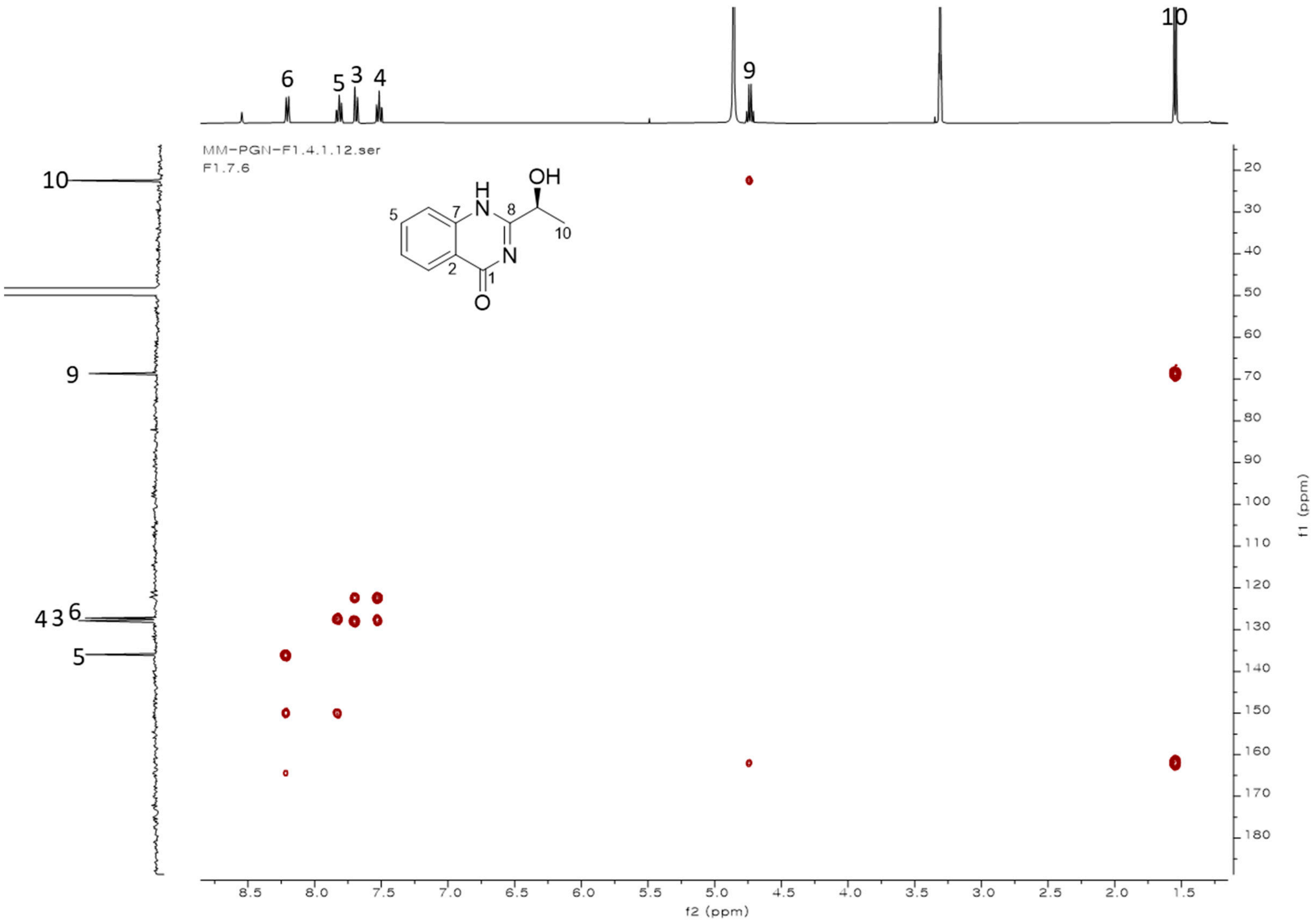

Figure S43. ^{1}H-^{13}C HMBC spectrum of **7** in CD_3OD

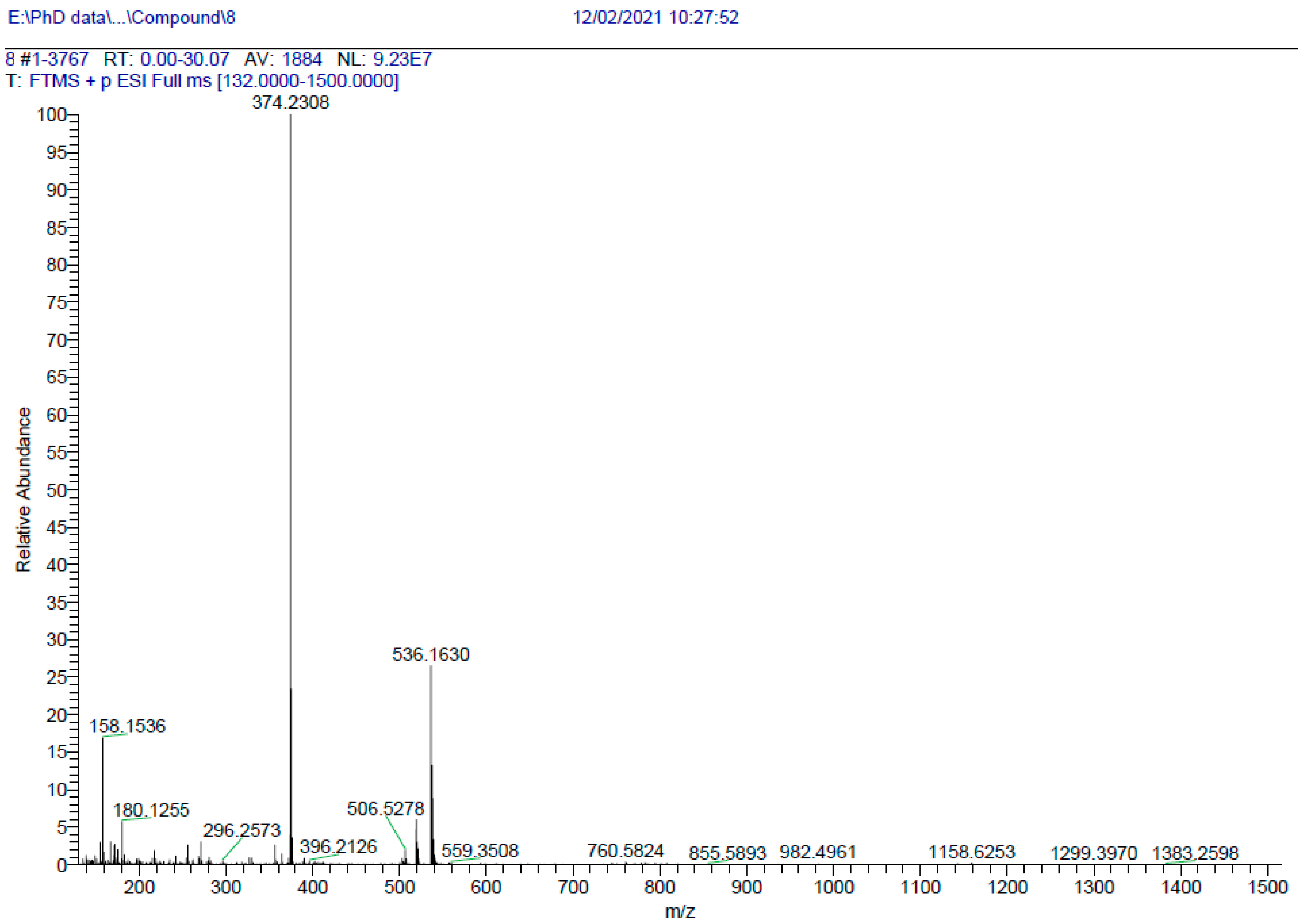

Figure S44. HRESI(+)MS of **8**

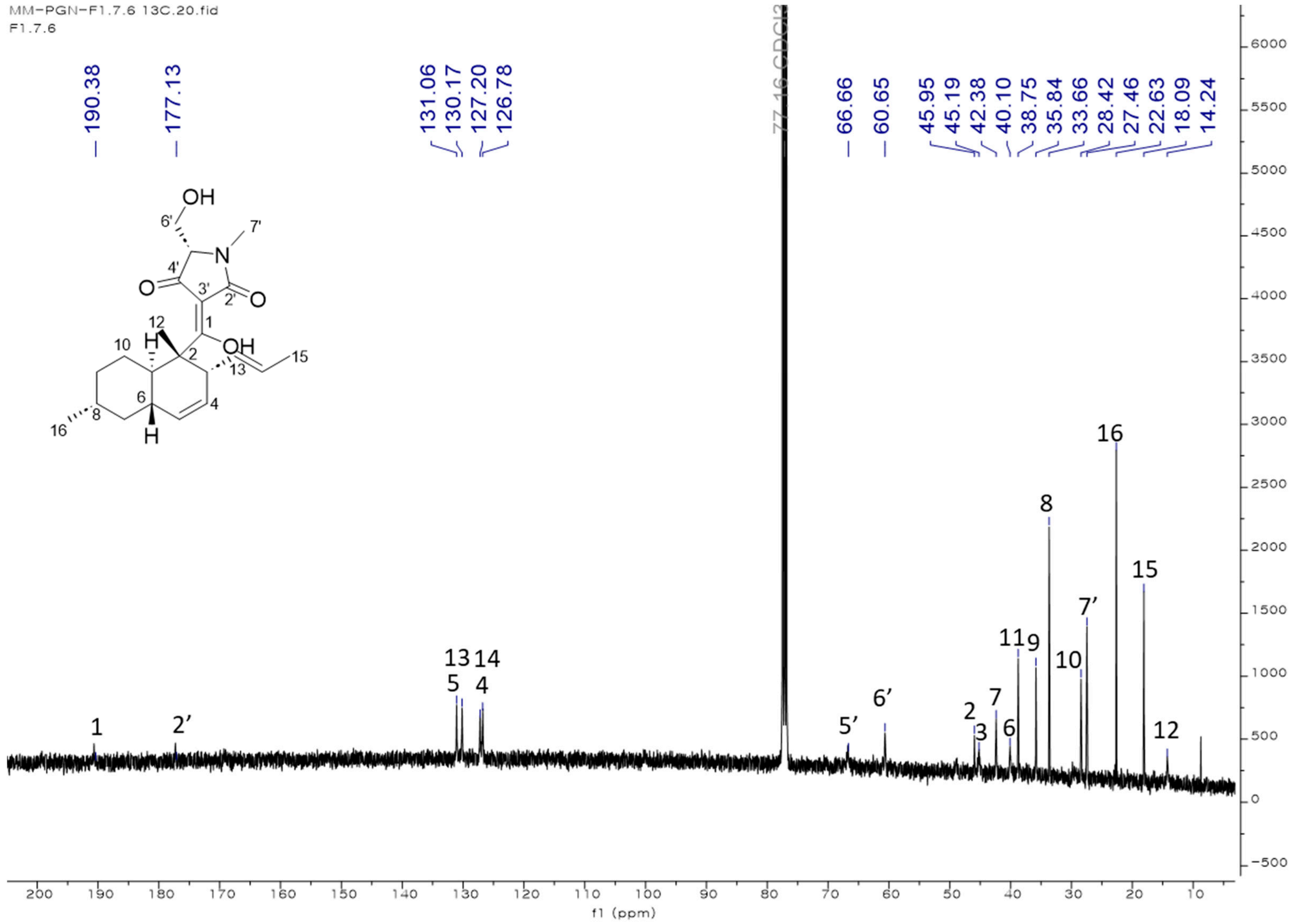

Figure S45. ^{1}H NMR (400 MHz) spectrum of **8** in $CDCl_3$

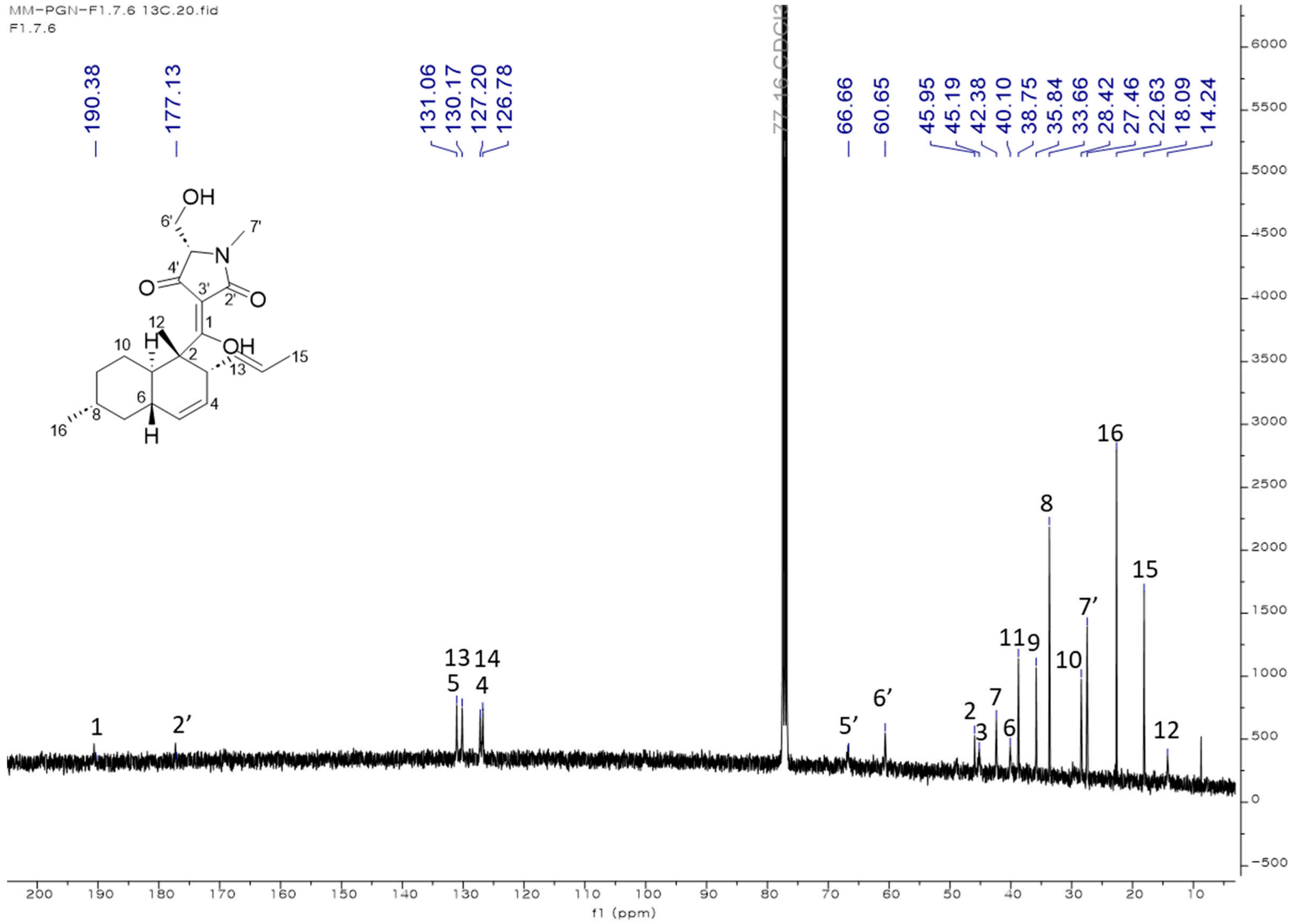

Figure S46. ^{13}C NMR (100 MHz) spectrum of **8** in $CDCl_3$

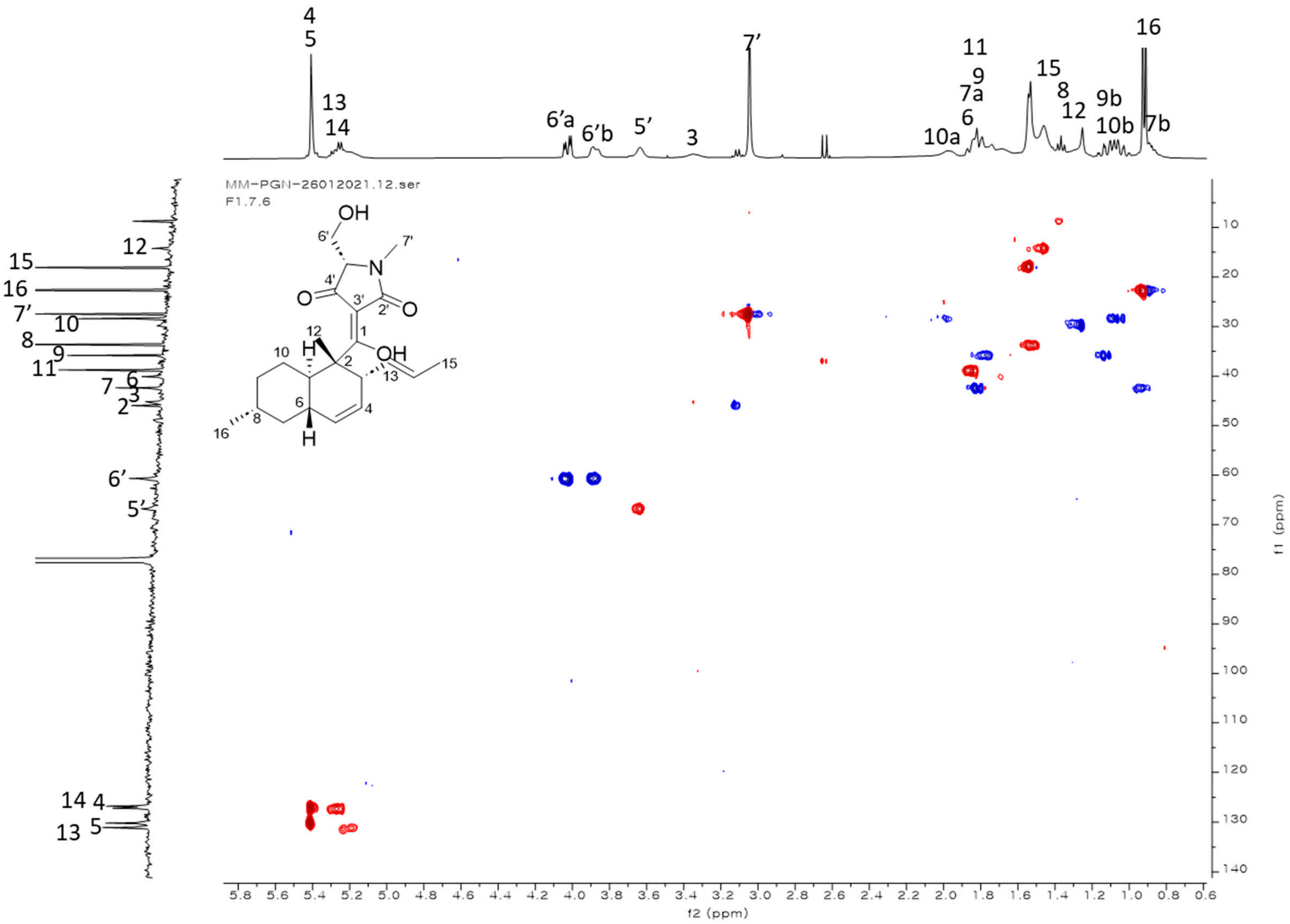

Figure S47. ^{1}H-^{13}C HSQC spectrum of **8** in $CDCl_3$

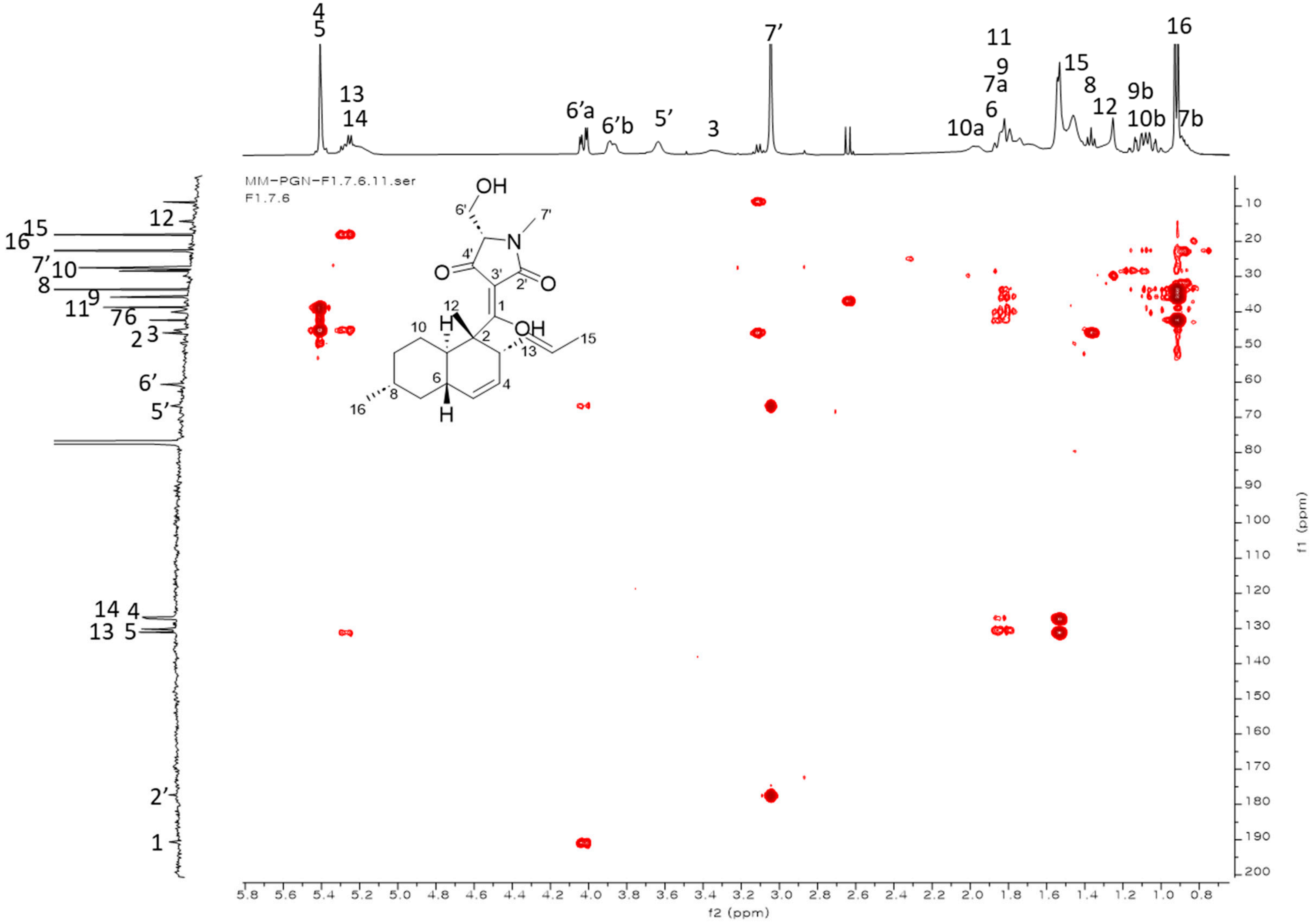

Figure S48. ^{1}H-^{13}C HMBC spectrum of **8** in $CDCl_3$

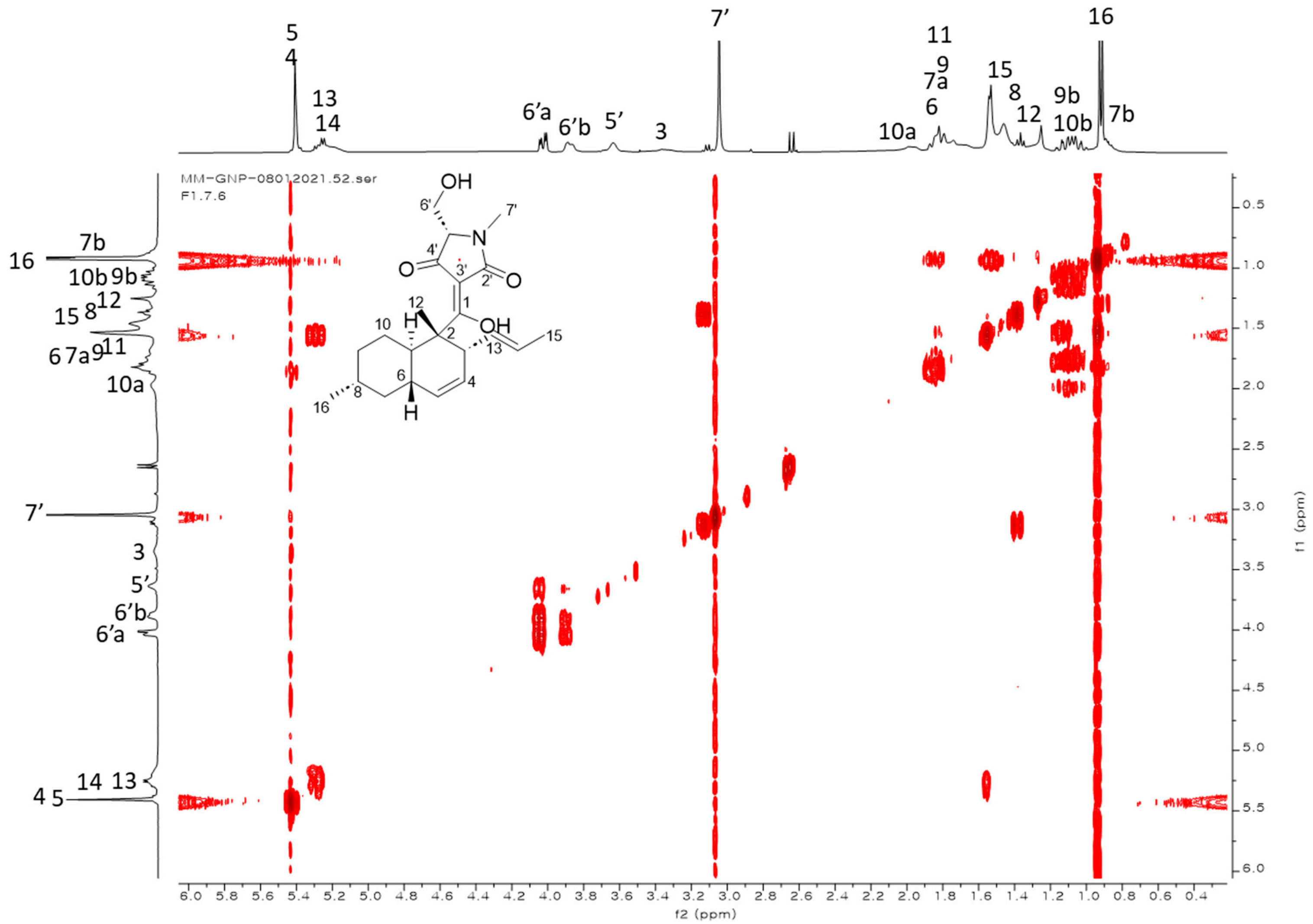

Figure S49. ^{1}H-^{1}H COSY spectrum of **8** in $CDCl_3$

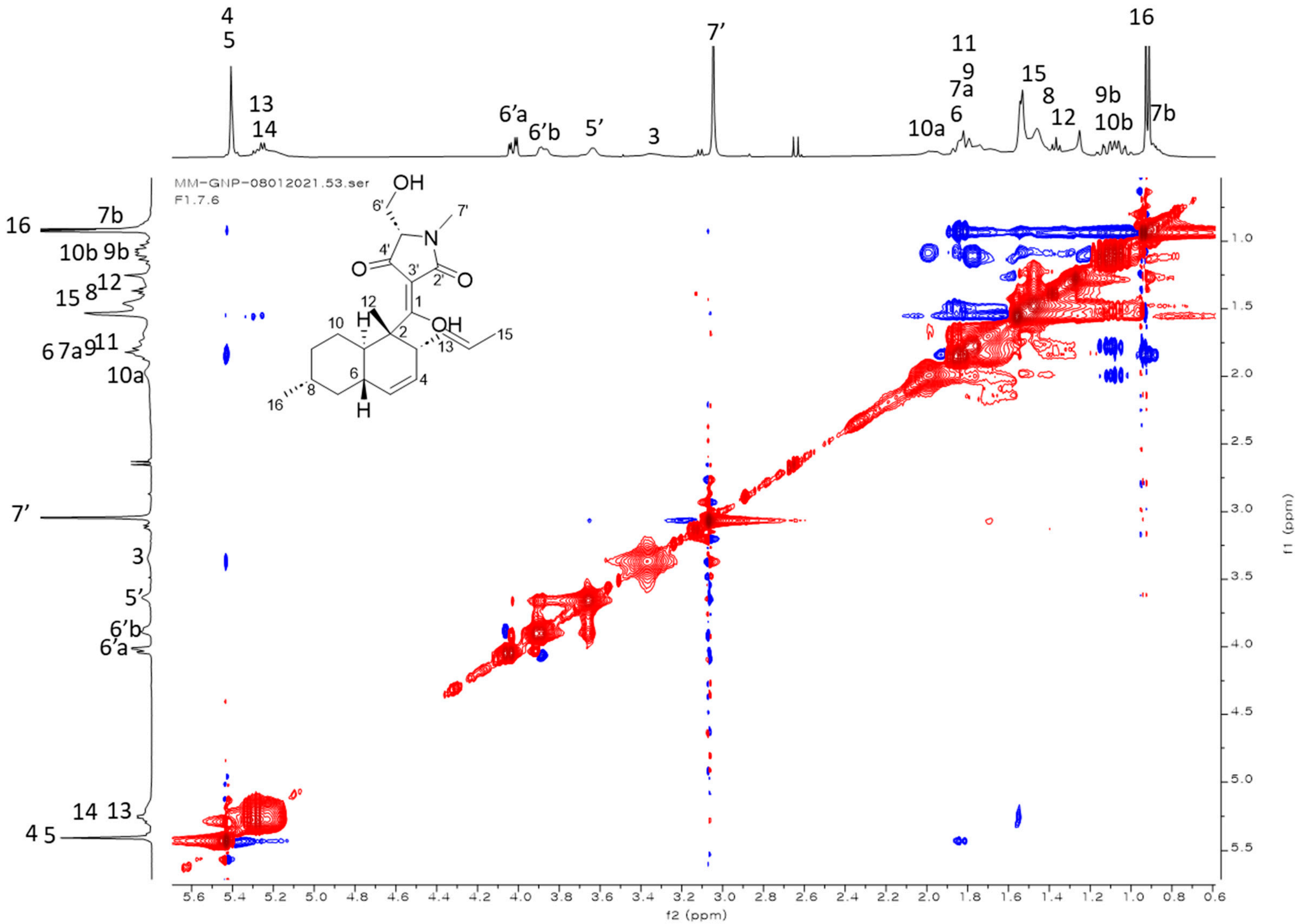

Figure S50. ^1H-^1H NOESY spectrum of **8** in $CDCl_3$

Table S1. Primary screening of compounds **1-8** against a set of 14 disease-related protein kinases. The % of remaining kinase activities are reported on the table.

Compound Id	Concentration	CDK5/p25	CDK9/CyclinT	HASPIN	*Rn*-DYRK1A*	AURKB	GSK3β	EGFR	ABL1**	JAK3**	EphB1**	VEGFR2 (KDR)	Pim1	CK1e	CLK1
**6**	**10 µM**	59	94	75	≥100	≥100	92	98	≥100	≥100	≥100	86	90	94	93
	**1 µM**	76	99	74	91	≥100	≥100	≥100	97	98	≥100	≥100	≥100	≥100	≥100
**1**	**10 µM**	89	97	67	71	≥100	≥100	≥100	≥100	99	≥100	≥100	67	≥100	≥100
	**1 µM**	≥100	≥100	67	76	≥100	≥100	≥100	≥100	72	≥100	≥100	80	≥100	≥100
**3**	**10 µM**	≥100	≥100	63	84	≥100	≥100	≥100	96	74	96	≥100	70	≥100	≥100
	**1 µM**	83	98	73	70	≥100	≥100	≥100	91	64	61	≥100	67	95	≥100
**4**	**10 µM**	96	96	65	86	≥100	≥100	≥100	64	55	62	≥100	73	97	≥100
	**1 µM**	≥100	98	72	76	≥100	92	≥100	85	75	≥100	75	78	98	≥100
**5**	**10 µM**	≥100	≥100	71	79	≥100	79	52	63	52	36	48	76	89	≥100
	**1 µM**	82	≥100	75	77	≥100	79	71	68	63	≥100	84	65	91	≥100
**2**	**10 µM**	≥100	≥100	75	75	97	82	48	45	30	59	72	78	99	≥100
	**1 µM**	99	≥100	88	70	≥100	84	89	63	65	95	53	77	≥100	≥100
**7**	**10 µM**	≥100	93	75	70	≥100	82	≥100	58	61	≥100	61	80	98	≥100
	**1 µM**	≥100	≥100	78	83	≥100	78	≥100	77	70	94	≥100	81	≥100	≥100
**8**	**10 µM**	≥100	≥100	84	92	≥100	≥100	≥100	81	78	≥100	≥100	78	≥100	≥100
	**1 µM**	98	≥100	89	80	≥100	90	89	78	74	69	70	62	≥100	≥100
**NB : ≥100 indicates that the compound cannot inhibit the enzymatic activity at the tested concentration**															

** Rn, Rattus norvegicus*
**** The kinases selected for the IC_{50} determination are highlighted in orange**

KISSf Id	Compound Id	ABL1	JAK3	EphB1
MaRC3-4/05	5	n.d.	25.16	1.42
MaRC3-4/06	2	23.83	25.48	n.d.

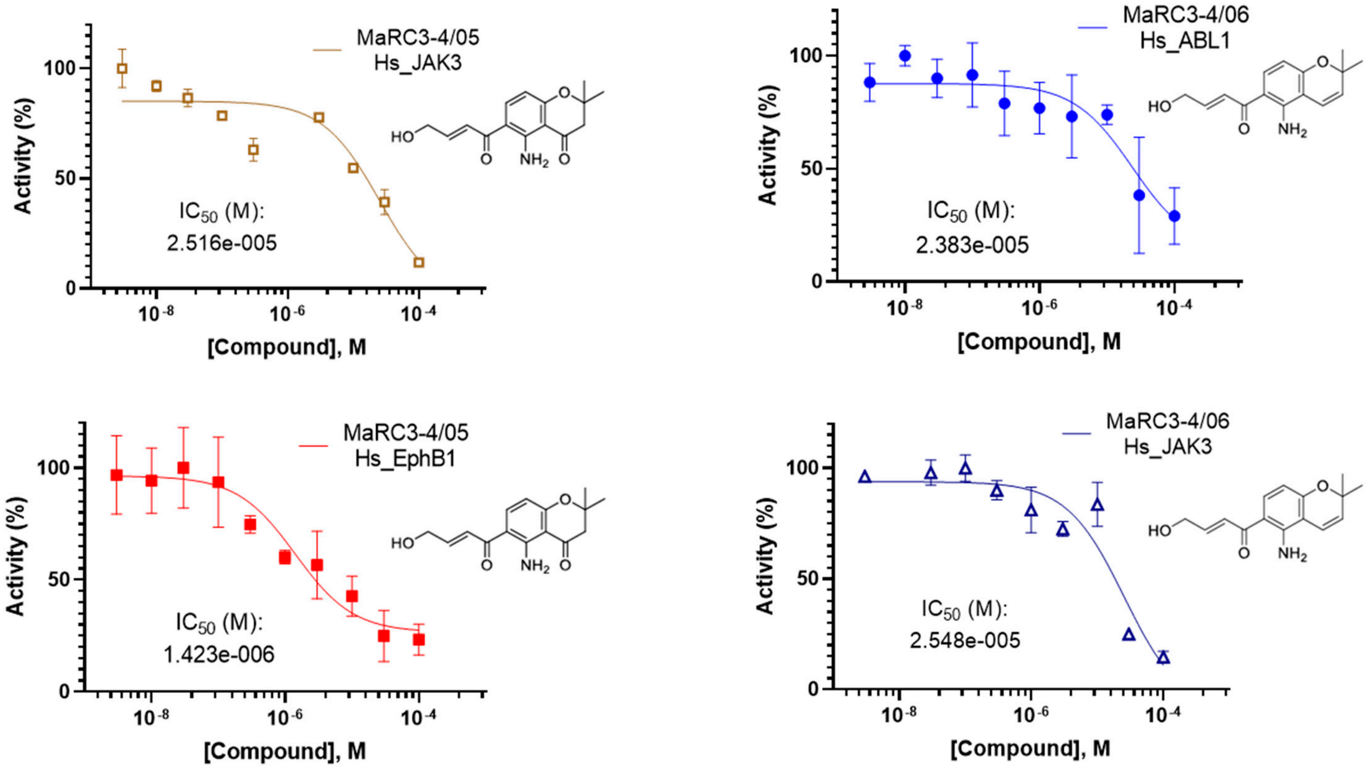

Figure S51. IC_{50} (μM) for compounds **2** and **5** against the selected protein kinases

Table S2. Primary screening of compounds **1-8** against RPE-1, HCT-116, and U2OS cell lines

Compound Id*	RPE-1		HCT-116		U2OS	
	**Mean**	**SD**	**Mean**	**SD**	**Mean**	**SD**
**1**	21.2	0.4	23.2	0.4	15.5	0.3
**2**	6.7	1.5	20.9	5.1	34.2	1.5
**3**	59.7	0.9	69.8	1.1	59.1	3.1
**4**	58.8	3.6	78.0	1.0	56.6	1.6
**5**	12.3	0.1	8.4	0.0	10.1	1.5
**6**	20.4	0.2	23.2	1.3	11.9	1.3
**7**	99.4	1.7	98.4	2.8	98.4	0.7
**8**	93.3	2.1	96.6	1.7	100.7	6.3

* Each compounds were tested at 25 μM in duplicate

The results are expressed as % of cell viability (100% of cell viability were measured with 0.25 % v/v DMSO)
